# PISOX Copolyesters—Bio- and CO_2_-Based Marine-Degradable High-Performance Polyesters

**DOI:** 10.1021/acssuschemeng.4c02266

**Published:** 2024-06-18

**Authors:** Kevin van der Maas, Yue Wang, Daniel H. Weinland, Robert-Jan van Putten, Bing Wang, Gert-Jan M. Gruter

**Affiliations:** † Van’t Hoff Institute of Molecular Sciences, University of Amsterdam, Science Park 904, 1098 XH Amsterdam, The Netherlands; ‡ Avantium Chemicals BV; Zekeringstraat 29, 1014 BV Amsterdam, The Netherlands

**Keywords:** PISOX, polyoxalate, isosorbide, oxalic acid, high *T*_g_, high-performance plastic, catalyst-free, biobased plastic, CO_2_ utilization (CCU), marine-degradable plastic, soil-degradable plastic, biodegradable packaging, home compostable plastic

## Abstract

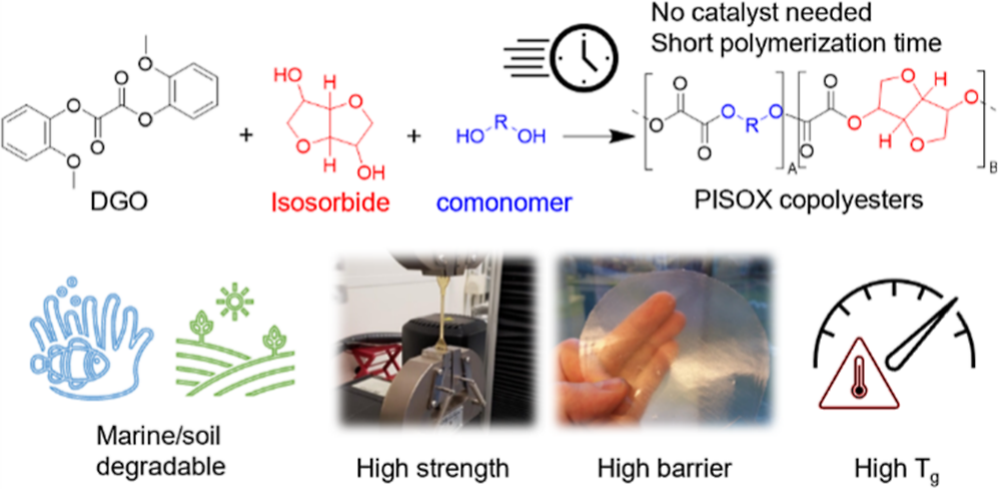

Oxalate esters and isosorbide serve as intriguing polymer building blocks, as they can be sourced from renewable resources, such as CO_2_ and glucose, and the resulting polyesters offer outstanding material properties. However, the low reactivity of the secondary hydroxyl groups makes it difficult to generate high-molecular-weight polymers from isosorbide. Combining diaryl oxalates with isosorbide appears to be a promising approach to produce high-molecular-weight isosorbide-based polyoxalates (PISOX). This strategy seems to be scalable, has a short polymerization time (<5 h), and uniquely, there is no need for a catalyst. PISOX demonstrates outstanding thermal, mechanical, and barrier properties; its barrier to oxygen is 35 times better than PLA, it possesses mechanical properties comparable to high-performance thermoplastics, and the glass transition temperature of 167 °C can be modified by comonomer incorporation. What makes this high-performance material truly exceptional is that it decomposes into CO_2_ and biomass in just a few months in soil under home-composting conditions and it hydrolyzes without enzymes present in less than a year in 20 °C water. This unique combination of properties has the potential to be utilized in a range of applications, such as biomedical uses, water-resistant coatings, compostable plastic bags for gardening and agriculture, and packaging plastics with diminished environmental impact.

## Abstract 2

PISOX: A home-compostable and marine-degradable high-performance plastic with remarkable properties, which can reduce microplastic pollution and fossil feedstock dependence.

## Introduction

At the moment, almost all of our plastics are produced from fossil-based resources. In order to move to a more sustainable future, there is a high interest in replacing fossil-based plastics with novel biobased plastics. To be able to fully replace them, similar or preferably better material properties are needed. Here, a new renewable material with unique properties has the benefit that it can compete on performance, while renewable drop-in plastics such as bio-PE and bio-PET can only compete on carbon footprint and price.^1^ Preferred or improved properties include high glass transition temperature, good mechanical strength, and good barrier properties.^2^ This current research focuses on novel CO_2_- and biobased plastics from oxalate esters and isosorbide. Both of these building blocks are available from renewable resources and are known to provide good material properties. From a cost (atom efficiency and energy investment) point of view, oxalic acid is the most attractive product obtainable from CO_2_.^1^ Oxalic acid production from CO_2_ through ambient temperature electrochemical reduction is currently under development and is an excellent example of carbon capture and utilization.^3^ Polyoxalates, such as polyethylene oxalate, are known to have good mechanical properties, undergo remarkably fast hydrolysis, and are, as a consequence, readily biodegradable.^4^ Oxalic acid is the simplest dicarboxylate: both carboxyl groups are directly connected to each other, which provides exceptional acidity, resulting in high reactivity and rigidity. Isosorbide is a biobased chemical that can be obtained from the dehydration of sorbitol and is produced on an industrial scale with a capacity of at least 20 kilotons per year.^5^ It is a rigid, chiral, and nontoxic molecule containing two secondary hydroxyl groups. Isosorbide already found its commercial use as a comonomer in PET and as a replacement for bisphenol A in polycarbonates. These and other isosorbide-based polymers are known for their high thermomechanical stability, good mechanical properties, and good barrier properties.^6^ However, it tends to be difficult to incorporate isosorbide into the polymer chain due to the low reactivity of the secondary hydroxyl groups. Incorporation of isosorbide in the polymer chain typically results in lower molecular weight polymers. In order to obtain sufficiently high molecular weights, the amount of isosorbide used is typically low.^5−7^ Strategies to obtain high proportions of isosorbide in the chain require long reaction times and high temperatures or hazardous solvents and reactants. Also, the use of oxalic acid in the synthesis of polyoxalates with high glass transition temperatures presents challenges. The higher temperatures required for melt polycondensation prevent oxalic acid from being used directly. Depending on the conditions, oxalic acid and oxalic acid end groups are prone to decomposition via decarboxylation at temperatures ranging from 127 to 157 °C.^8^ Oxalic acid also has a substantial vapor pressure at temperatures above 100 °C and quickly sublimes.^9^ Both processes distort the polymerization process, limiting the molecular weight. In addition to CO_2_, formate is another thermal decomposition product that can be generated. Formate only forms single ester bonds, capping the polymer chain and preventing further chain growth. To prevent this, the alkyl ester of oxalic acid can be used such as dimethyl- or diethyl oxalate, but also here the low reactivity of isosorbide in the required transesterification results in low molecular weight and low incorporation of isosorbide.^4,10^ Instead, the more reactive acyl chloride of oxalic acid is often used.^11^ However, on the larger (industrial) scale, this would come with significant drawbacks, such as the use of hazardous solvents, production of corrosive side products, and high operating costs. An alternative viable strategy, often seen in polycarbonate production, is the synthesis of high molecular weight isosorbide-based polycarbonates by the transesterification of diphenyl carbonate.^12,13^ This strategy already has shown to be economically viable and is steadily replacing the traditionally used phosgene-based processes.^14−16^ Also diphenyl oxalate is commercially available, as it is a precursor for the production of diphenyl carbonate and carbamates.^17,18^ Despite the availability of diphenyl oxalate, its use as a monomer for polyoxalate polyesters has been rather unexplored. Except for our own research group^19^ and the diphenyl oxalate producer UBE,^20^ no other literature was found utilizing this strategy. Since other strategies did not obtain sufficiently high molecular weight isosorbide-based polyoxalates, little is known about the physical and mechanical properties of PISOX and its copolymers where a second diol or a second diacid is involved.^7^

**Figure 1:**
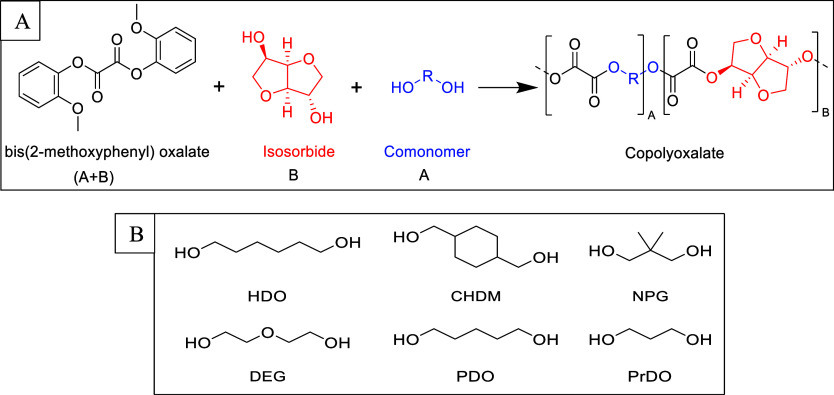
(A) Reaction scheme of the general polymerization of PISOX copolymers. (B) Different comonomers used in this research.

To address the toxicity of the leaving phenol, the use of the potentially nontoxic substituted phenol guaiacol (2-methoxyphenol) was explored. Since diguaiacyl oxalate (DGO) is not commercially available, two synthesis strategies were investigated: one via oxalyl chloride and the other via the transesterification of dimethyl oxalate.^17,21^ The reactivity of DGO was compared to those of other commonly found polyester building blocks: terephthalate and carbonate, by studying equilibrium reactions with isosorbide. To map the physical properties of PISOX copolymers, several commonly available diols were copolymerized in different ratios with isosorbide and DGO (Figure 1). The resulting polymers were analyzed for their molecular weight, thermal properties, barrier properties, mechanical properties, and biodegradability in soil and water. Lastly, a PISOX copolymer with diethylene glycol as the comonomer was produced on a kilogram scale from DGO in a 2 L autoclave and was further processed into a filament for 3D printing.

## Results and Discussion

### Reactivity of DGO Compared to Other Aryl Esters

**Figure 2:**
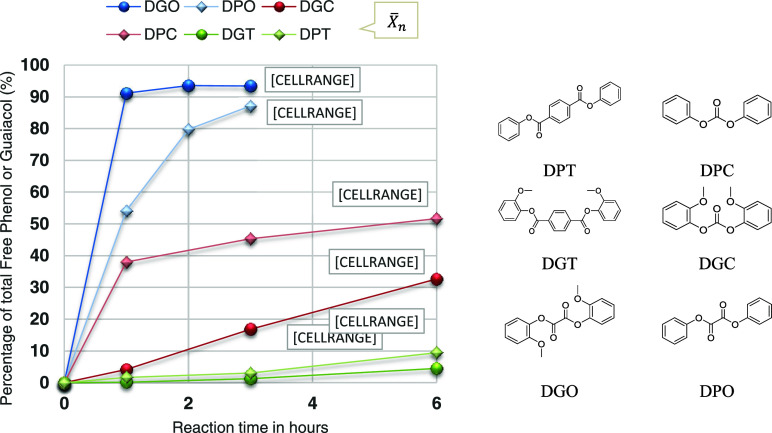
Reaction of diphenyl oxalate (DPO), diguaiacyl oxalate (DGO), diphenyl carbonate (DPC), diguaiacyl carbonate (DGC), diphenyl terephthalate (DPT), and diguaiacyl terephthalate (DGT) with equimolar isosorbide in a closed system at 190 °C. The release of guaiacol and phenol is shown as a percentage of the total phenol (ester + free). The data labels show the degree of polymerization (), see eqs S1 and S2 in the Supporting Information for the calculation. See Table S4 and Figures S30–S35 for more detailed experimental results.

To explore the reactivity of aryl oxalate esters, their reactivity was compared to other commonly used polyester building blocks; terephthalate and carbonate. The phenyl and guaiacyl esters of the dicarboxylates were reacted in an equimolar ratio with isosorbide. The transesterification reaction was carried out in a closed system with no added catalyst at a set temperature of 190 °C. The reactions were sampled over time and analyzed by ^1^H NMR. The progress of the reaction was followed by the amount of guaiacol, or phenol released (Figure 2).

Looking at the reactivity of the different esters, oxalate clearly shows the highest reactivity, followed by carbonate and then terephthalate. The high degree of polymerization observed for oxalate indicates that there are already short polymer chains formed within a couple of hours. After just 2 h, DGO has an average molecular weight of 1.6 kDa (eq S3), which is remarkably high considering that no condensation product is removed. In contrast, for carbonate and terephthalate, there are still mainly monomers present after 6 h, which is an indication that a catalyst and/or higher temperatures are needed to accelerate the reaction, assuming that there is no large difference in the rate of the reverse reactions. The high reactivity of oxalate can probably primarily be attributed to the vicinal carboxyl groups of oxalate, which generate an inductive effect on each other, making the carboxylic acids more acidic and thereby further promoting the esterification process. This inductive effect is evident in the remarkably low p*K*_a_ value of oxalic acid (1.19), which is considerably lower than that of other carboxylic acids.^22^ Furthermore, the reactivity among the various esters is influenced by the accessibility of the ester bonds, particularly the acyl portion of the ester.^23^ This might explain the experimentally observed difference in reactivity between the carbonate and terephthalate esters, as the terephthalate has a more “bulky” structure. When the reactivity of guaiacyl esters is compared with that of phenyl esters, it is evident that the phenyl esters of carbonate and terephthalate exhibit faster reactivity than the guaiacyl esters. The reduced reactivity of guaiacyl esters is likely due to the steric hindrance caused by the methoxy group, resulting in a higher activation energy required for transesterification. However, this same steric hindrance also decelerates the reverse reaction of guaiacol with the polymer chain. This could explain why in the case of the oxalate the guaiacyl ester reactivity exceeds the phenyl esters, as the reverse reaction plays a more dominant role when approaching the equilibrium state (at which the polymer and free phenol/guaiacol concentration is highest). Another possible explanation is that the oxalate reaction follows an alternative reaction pathway that is less affected by steric hindrance. Considering the more difficult reaction of guaiacol with the polymer chain, it is likely that for all guaiacyl esters, an equilibrium state at a higher degree of polymerization can be reached than for the phenyl esters. In our experiments (Figure 2), only DGO reaches its equilibrium state with a high k-value of ∼200 and DPO seems to approach equilibrium after 3 h (eq S1 in Supporting·Information). All other esters still seem far away from equilibrium, even after 6 h reaction at 190 °C. The equilibrium state found for DGO is considerably higher compared to the reaction equilibrium values mentioned (0.1 to 1.0) for typical nonphenolic alcohols used in polyester synthesis, such as ethylene glycol or 1,4-butanediol with terephthalic acid to produce PET or PBT.^24^ In similar carbonate polymerization chemistry, advantages in reactivity were also observed for ortho-substituted phenyl groups: in the work of Kamps et al., bis(methylsalicyl) carbonate showed reactivity benefits over diphenyl carbonate.^25,26^ The reactivity benefits of DGO can be used to make the polymerization catalyst-free and/or milder and shorter, resulting in better color and fewer side reactions. It also opens up the possibility of polymerizing with less thermally stable diols or performing polymerization in solution. Guaiacol also has several practical benefits as a leaving group; its melting temperature is close to room temperature, which makes polycondensation on a larger scale easier, as it does not require trace-heated distillation pathways. Guaiacol is nontoxic, making it safer to work with than phenol.

### DGO Synthesis and Recycling

DGO can be synthesized via oxalyl chloride, which is an easy and fast method for smaller (laboratory) scale production. However, for larger scales, this is not a feasible route considering the use of hazardous chloride components and high amounts of solvent. Therefore, we also explored and demonstrated the production of DGO via the transesterification of dimethyl oxalate with guaiacol in a 2 L autoclave (see Supporting Information for the protocol). This is also useful for the recycling process. The guaiacol, which is collected as a condensate during the polymerization, can be purified by distillation and reused for the production of DGO via dimethyl oxalate. Alternatively, guaiacol itself can be used for various applications, including as a flavoring agent in food and beverages, a fragrance component in perfumes and cosmetics, a precursor in the synthesis of pharmaceuticals, and as an indicator in chemical tests.^27^

### Structural Characterization

Different PISOX (co)polymers were synthesized via diguaiacyl oxalate on about a 25 g scale using the following primary diols in combination with isosorbide and DGO: 1,6-hexanediol (HDO), 1,5-propanediol (PDO), 1,3-propanediol (PrDO), neopentyl glycol (NPG), diethylene glycol (DEG), and cyclohexane-1,4-dimethanol (CHDM). The resulting polymers were analyzed for their molecular weight, molar composition, and thermal properties (Table 1).

**Table 1:** Molecular Composition, Molecular Weight, and Thermal Properties of the PISOX Copolymers^a^

analysis method	feed	NMR	NMR	GPC	GPC	GPC	DSC^b^	TGA^b^^,^^c^	TGA^b^^,^^d^
type of diol	molar content (%)	molar content (%)	*M*_n_ (kg/mol)	*M*_n_ (kg/mol)	*M*_w_ (kg/mol)	*D̵* (*M*_w_/*M*_n_)	*T*_g_ (°C)	*T*_d-5%_ (°C)	*T*_d-max_ (°C)
CHDM 50%	49.9	50.0	54.5	48.8	89.7	1.84	101	329	355
HDO 37.5%	37.6	37.3	32.9	54.4	105.0	1.93	76	327	353
HDO 25%	25.0	24.7	23.6	44.0	81.7	1.85	107	327	352
PDO 37.5%	37.6	36.4	36.7	38.6	69.7	1.81	85	326	351
PDO 25%	25.2	24.5	59.4	28.3	49.4	1.75	110	330	352
NPG 50%	50.0	50.0	31.7	35.6	63.3	1.78	83	337	361
NPG 37.5%	37.6	37.0	77.9	68.5	133.0	1.95	102	337	359
PrDO 50%	49.5	49.0	33.1	37.1	56.5	1.52	85	n.d	n.d
DEG 37.5%	37.5	37.5	18.9	29.9	48.2	1.61	88	329	353
PISOX 100%	0	0	40.0	39.6	78.3	1.98	167	331	356

a The molar content in the Table corresponds to the amount of co-diol relative to the total diol amount, for example, HDO25% contains 25% HDO, 75% isosorbide, and 100% Oxalate. See Supporting Information (“Calculations molecular weight by ^1^H NMR”) for the calculations. For the NMR spectra see Figures S17–S27 and for the GPC data see Table S9 and Figures S62–S64.

b Temperatures are obtained under a nitrogen atmosphere.

c *T*_d-5%_ is the temperature at which 5% of the initial mass was lost.

d *T*_d-max_ is the temperature where the thermal degradation rate is the highest.

Based on the GPC measurements, weight-average molecular weights (*M*_w_) range from 48 to 133 kg/mol, and number-average molecular weights (*M*_n_) from 28 to 69 kg/mol with a *D̵* (*M*_w_/*M*_n_) close to 2 for all polyesters. NMR analysis confirms the high molecular weights, with *M*_n_ values ranging from 19 to 78 kg/mol. These values are significantly higher than the reported molecular weights for the synthesis of PISOX from oxalic acid or diethyl oxalate, which resulted in low-molecular weight (1.7 kg/mol) tar-like polymers.^11,19^ The strategy used in this research, where a highly reactive aryl ester of oxalic acid is used, solves the problems encountered with the low reactivity of isosorbide and the thermal lability of oxalic acid. The increased reactivity of the aryl group makes polymerization conditions relatively mild, and the reaction times are relatively short. The complete polymerization can be performed in several hours, even when no catalyst is added. Additionally, because of the high reactivity of DGO and the reflux effect of the unbound guaiacol, there is no need to use excess diol to compensate for diol losses. Even the relatively volatile codiol PrDO is almost fully incorporated, as can be seen from the comparison between the content in the feed and the NMR analysis results. As far as we know, this is currently the only strategy that can produce polyoxalates from isosorbide with high molecular weights, also applicable for larger-scale production. Overall this work demonstrates the reactive nature of aryl oxalates and its potential for the production of high *T*_g_ polyesters without catalysts and high-molecular weight, which is not possible with any other known method.

From the perspective of sustainability and toxicity, the ability to synthesize high molecular weight polymers without the need for catalysts is highly intriguing. Should the polymer find its fate in nature, where it is subsequently composted, there will be no release of metal salts such as Sn, Sb, and Ge which are often used as catalysts in polyester synthesis. Catalyst-free polyesters also have advantages for food and medical applications, where leaching of the catalyst could be a concern.^28−30^ The presence of small quantities of metal catalyst utilized in polyester synthesis still presents a significant challenge, as recovery of these metal catalysts from discarded plastics is very complex and thus not feasible, ultimately leading to the depletion of essential raw materials. This issue is already prominent with antimony, the favored metal catalyst in PET production.^31,32^

### Thermal Properties

**Figure 3:**
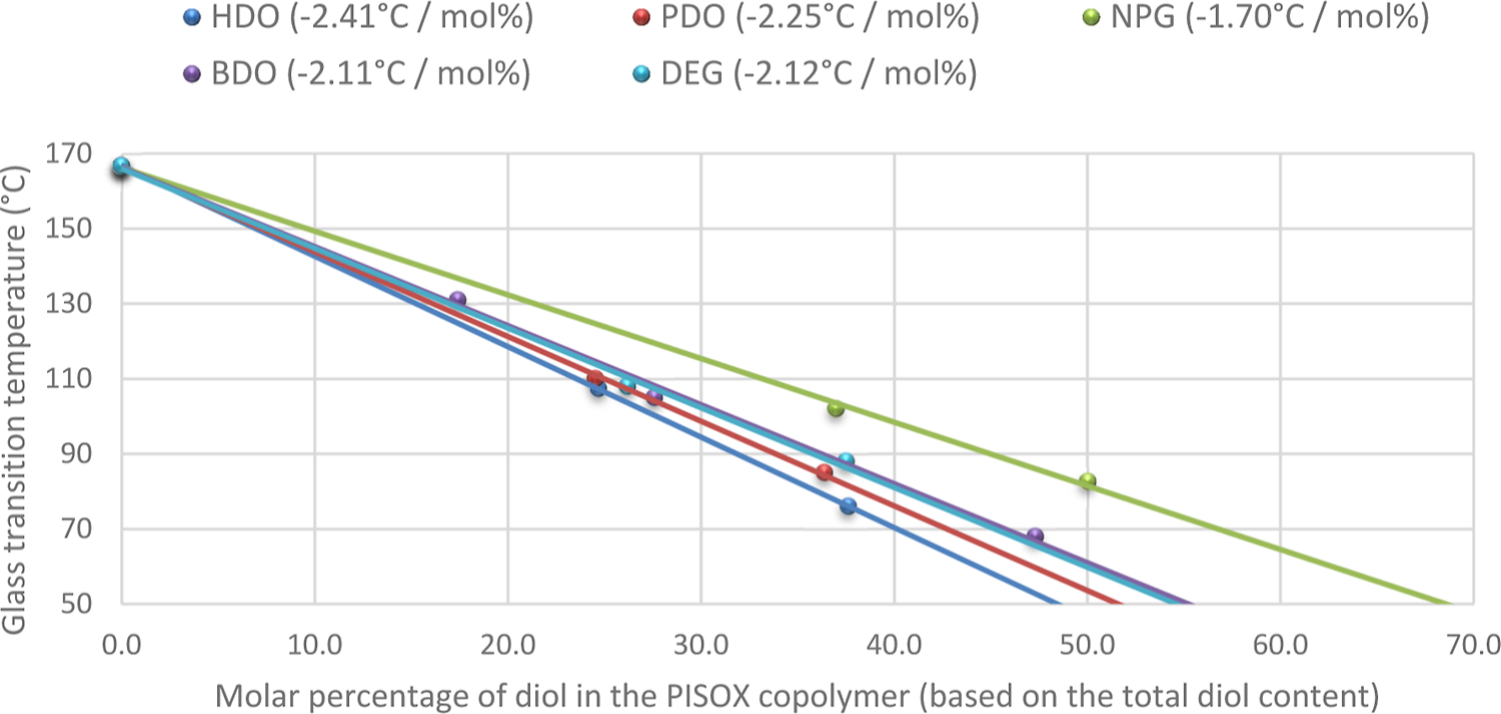
Molar diol content of the PISOX copolymer is varied and plotted against the *T*_g_. A linear trend line is fitted to the data points and the slope, intersect, and *R*^2^ of the trend line are given in Table S3. See also Figures S28 and S29 for more details.

PISOX copolyesters are amorphous polymers with the possibility to tune the *T*_g_ up to 167 °C (without a comonomer). This allows for targeting the *T*_g_ of the PISOX toward the application by selecting the type and amount of the second diol. The Fox, Gordon–Taylor–Wood, Johnston, and Barton equations have been applied to predict the glass transition temperatures of copolymers.^33^ Whether which equation works best to predict the *T*_g_ depends on the type of copolymer system. In blends when two polymers are miscible without any strong interaction, the *T*_g_-composition curve usually follows the Fox equation.^34^ In the case of the Fox equation, the *T*_g_ is a function of the mass fraction, and in the case of the Barton equations, the molar fraction is used. Within our experimental window, a linear relationship between molar fraction and *T*_g_ holds true (*R*^2^ = 0.997 to 1, Figure 3), as well as the Fox equation gives a good prediction (see Supporting Information, Table S1 and Figure S3). This could be related to the fact that there are relatively small differences in *M*_w_ between the homopolymers (ISO-OX and Diol-OX). Interestingly, while DEG and 1,5-pentanediol have a similar number of atoms in the chain, the incorporation of the ether function of DEG results in a trend identical to the *T*_g_ dependency of the incorporation of 1,4-butanediol.

Apart from differences in *T*_g_, minor differences in thermal stability between the PISOX copolymers were observed (Table 1). The temperature at which an initial mass loss of 5% was recorded ranged from 326 to 337 °C, and the *T*_d-max_ ranged from 351 to 361 °C. These thermal decomposition temperatures are similar to the values found for poly isosorbide carbonates.^12,35^ Overall, PISOX exhibits good thermal stability and is stable at temperatures far above the softening temperatures used for processing. However, during compression molding, despite being well dried, bubbles easily formed at high temperatures (>200 °C). This made the processing of higher *T*_g_ PISOX copolymers more difficult, especially for the homopolymer. With injection molding, these problems did not occur.

### Density

The PISOX polymers had a density ranging from 1.38 to 1.44 g/mL, which is relatively high compared to other biodegradable biobased polyesters (Table 3). As its density is well above the density of water, PISOX polymers will sink in aquatic environments. This has a significant impact on its degradation pathway in marine environments when compared to floating polymers, and should thus be studied accordingly. We have reported the results of PISOX copolyester biodegradation and nonenzymatic hydrolysis in water elsewhere.^36^

### Barrier Properties of PISOX

**Figure 4:**
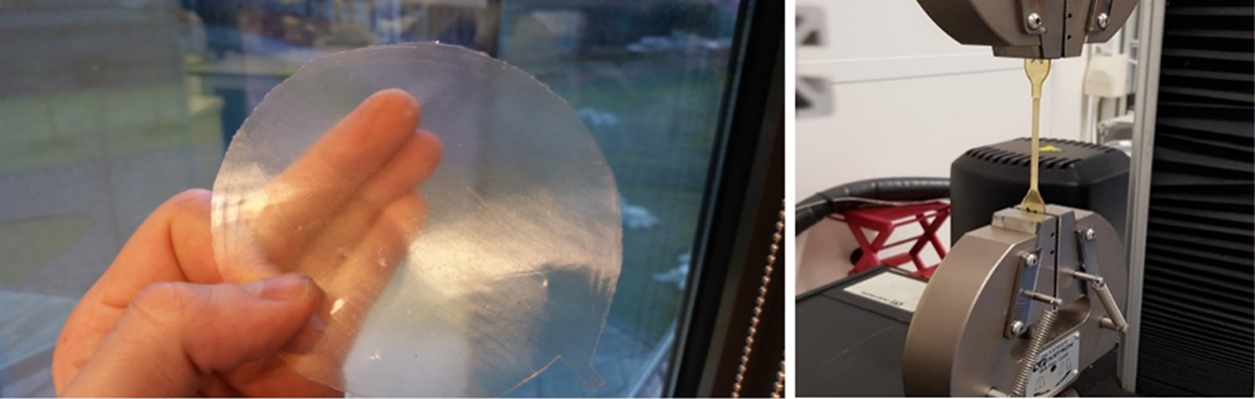
Compression molded film of the 25% HDO PISOX copolymer and a tensile bar being tested.

**Figure 5:**
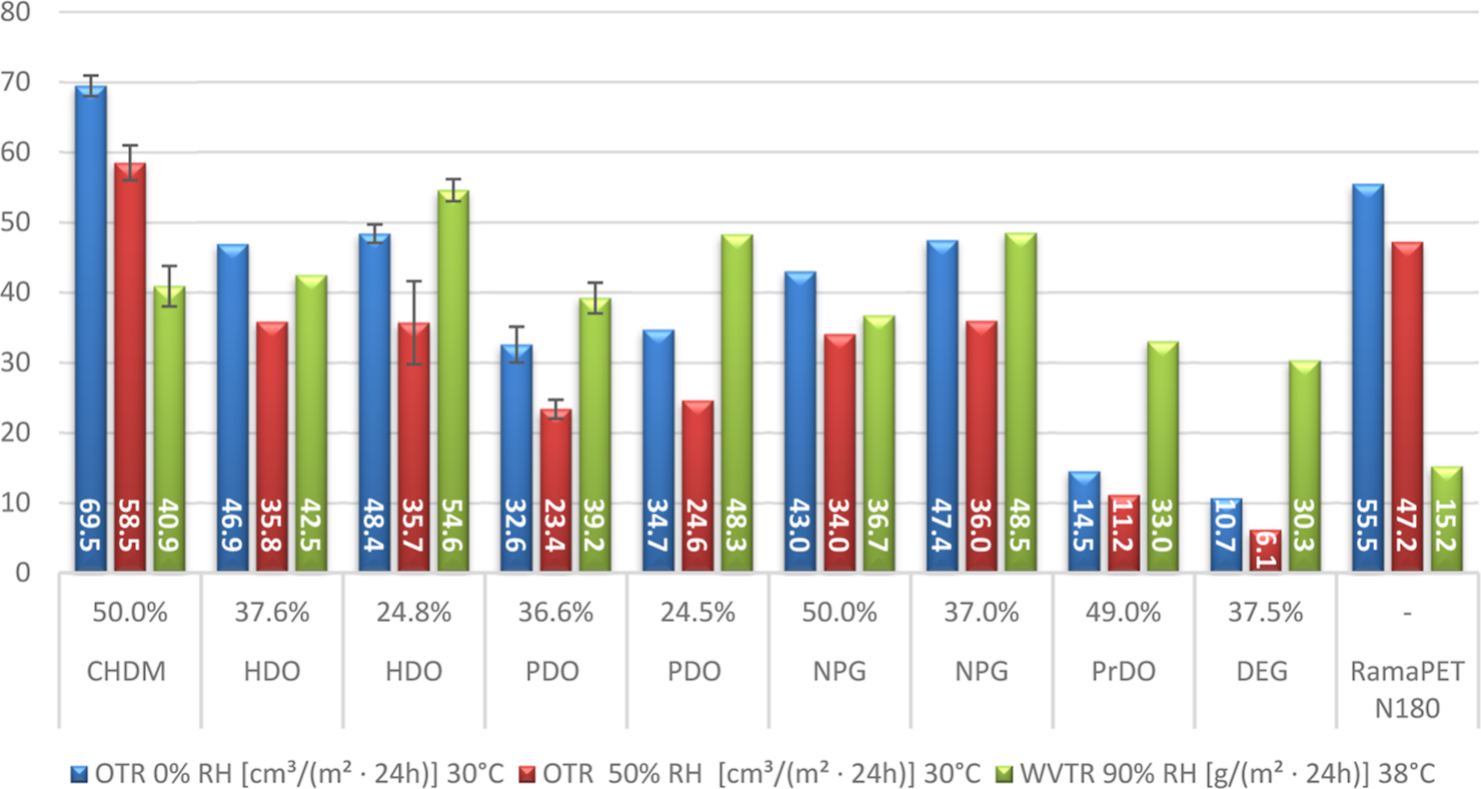
Water vapor (WVTR) and oxygen (OTR) permeability of different PISOX copolymers: The WVTR and OTR values are reported for a film thickness of 100 μm. Some values are averages of multiple films, and the standard deviation is shown by the error bars. All films were made by compression molding. The percentages provided represent the molar content of codiol in the polymer as determined through NMR analysis. See Table S5 and Figures S36–S49 for more details. The RamaPET film had a degree of crystallinity of 10%.

The gas permeability of the different PISOX polymers was measured by using thin films of approximately 100 μm thickness (Figure 4). The oxygen and water transmission rates of the different PISOX copolymers were measured, and the obtained results are shown in Figure 5.

For the PISOX copolymers, the OTR at 0% humidity (30 °C) ranges from 10.7 to 69.5 (cm^3^/(m^2^·24 h)), the OTR at 50% humidity (30 °C) ranges from 6.1 to 58.5 (cm^3^/(m^2^·24 h)), and the WVTR at 90% humidity (38 °C) ranges from 33.0 to 54.6 (g/(m^2^·24 h)).

The oxygen barrier appears to depend mostly on the relative humidity, chain length, and shape of the codiol. The oxygen barrier of all PISOX copolymers was enhanced by higher relative humidity. Upon comparison of the PISOX copolymers that contain aliphatic diols, it is clear that shorter chain length results in a better oxygen barrier. PrDO, the copolymer with the shortest diol, has the highest oxygen barrier performance, followed by PDO and HDO. Another significant factor is the shape of the codiol. Despite the fact that NPG and PrDO have the same chain length, the less bulky PrDO has a 3× better barrier. The same effect can be seen from the most bulky copolymer, CHDM, which has the worst oxygen barrier of all the copolymers. A remarkable copolymer worth highlighting is DEG, which has a chain length similar to PDO but contains an ether linkage, which results in an almost 4× better oxygen barrier than PDO. This could be explained by the polarity and improved flexibility of the ether linkage.^37^

Overall, the water barrier varies less by the type and content of codiol and thus seems to be less affected by the codiol. One trend which can be observed if we only consider the aliphatic codiols is that a higher content and shorter chain length of the codiol improves the water barrier. Also, the polarity of the codiol seems to play a role, as the most polar codiol, DEG, has the best water barrier.

PET has relatively good barrier properties and is therefore commonly used as packaging material for food and household applications. By comparing the barrier properties of PISOX with PET, its potential as a packaging material can be explored. When comparing the oxygen barrier of PISOX with PET, the oxygen barrier of PISOX is better than that of PET at a relative humidity of 0%, except for the copolymer, which contains 50% CHDM. At a more realistic relative humidity of 50%, the barrier of PISOX gets significantly better. When diethylene glycol is used as codiol, the oxygen barrier is up to almost 8 times higher than PET. The barrier properties also depend on the way the material is processed into a film, e.g., compression molding, film stretching, and solution casting. Stretching is the most common method for PET. Stretching allows better control over the orientation and crystallization of the polymer chain, which results in better improved barrier properties.^38,39^ For PET, it is known to significantly improve its barrier properties due to induced crystallization: reported values show three times improvement.^40^ However, as PISOX polymers are amorphous, it is difficult to estimate how orientation can improve the barrier. To have an equal comparison between materials, the commercial PET was processed in the same way as PISOX (compression molding).

After the transmission rate is converted to permeability, the barrier properties can be compared to literature values (Table 2). The permeability values for PET in our experiments are similar to the values for PET reported in the literature, indicating good reliability of barrier measurements. At present, the availability of biodegradable–biobased polymers possessing mechanical and barrier qualities that can rival conventional petroleum-based plastics is limited.^41^ The oxygen permeability (OP) of PISOX-DEG is very close to the reported value for amorphous PEF. This is thus much better than for most currently available biobased or biodegradable plastics, such as PLA and PBAT. In terms of oxygen barrier performance, PISOX-DEG demonstrates a barrier improvement factor (BIF) of 35 versus PLA and 117 versus PBAT. Regarding water barrier performance, PISOX-DEG demonstrates a BIF that is 2.4× higher than PLA and 9× higher than PBAT. While measured at a slightly lower temperature, the reported values for the homopolymer of PISOX exhibit an even better oxygen barrier, which matches that of oriented PEF.

**Table 2:** Overview of the (In-House) Measured Films and Literature-Reported Values^a^

type of film	OP [(cm^3^·μm)/(m2·24 h·atm)]	BIF	WVP [(g·μm)/(m2·24 h·kPa)]	BIF	references
Experimental Measurements					
PISOX-DEG37.5	606 (38 °C: 90%)	1	505 (38 °C: 90%)	1	
PET^b^	4720 (30 °C: 50%)	7.8	253 (38 °C: 90%)	0.5	
Literature					
PISOX	247 (23 °C: 50%)	0.4			(20)
PET (amorphous)	3940 (30 °C: 50%)	6.5			(42)
PET-oriented (mylar)	1680 (25 °C: 45%)	2.8	80 (38 °C: 90%)	0.2	(40)
PBAT (ecoflex)	70,851 (23 °C: 50%)	117	4470 (38 °C: 90%)	8.9	(41,43)
PLA	21,370–23,060 (28 °C: 50%)	35	1204 (38 °C: 100%)	2.4	(44)
bio-PE	54,356 (23 °C: 0%)	90	∼76 (38 °C: 100%)	0.2	(41)
PEF (amorphous)	722 (30 °C: 50%)	1.2			(42)
PEF (oriented)	∼270 (25 °C: 0%)	0.4			(45)

a The barrier improvement factor (BIF) relative to the PISOX-DEG 32.5% film is listed for both oxygen and water vapor.

b A visually clear film made by compression molding, with a crystallization percentage of 10%, measured by DSC.

### Mechanical Properties

**Figure 6:**
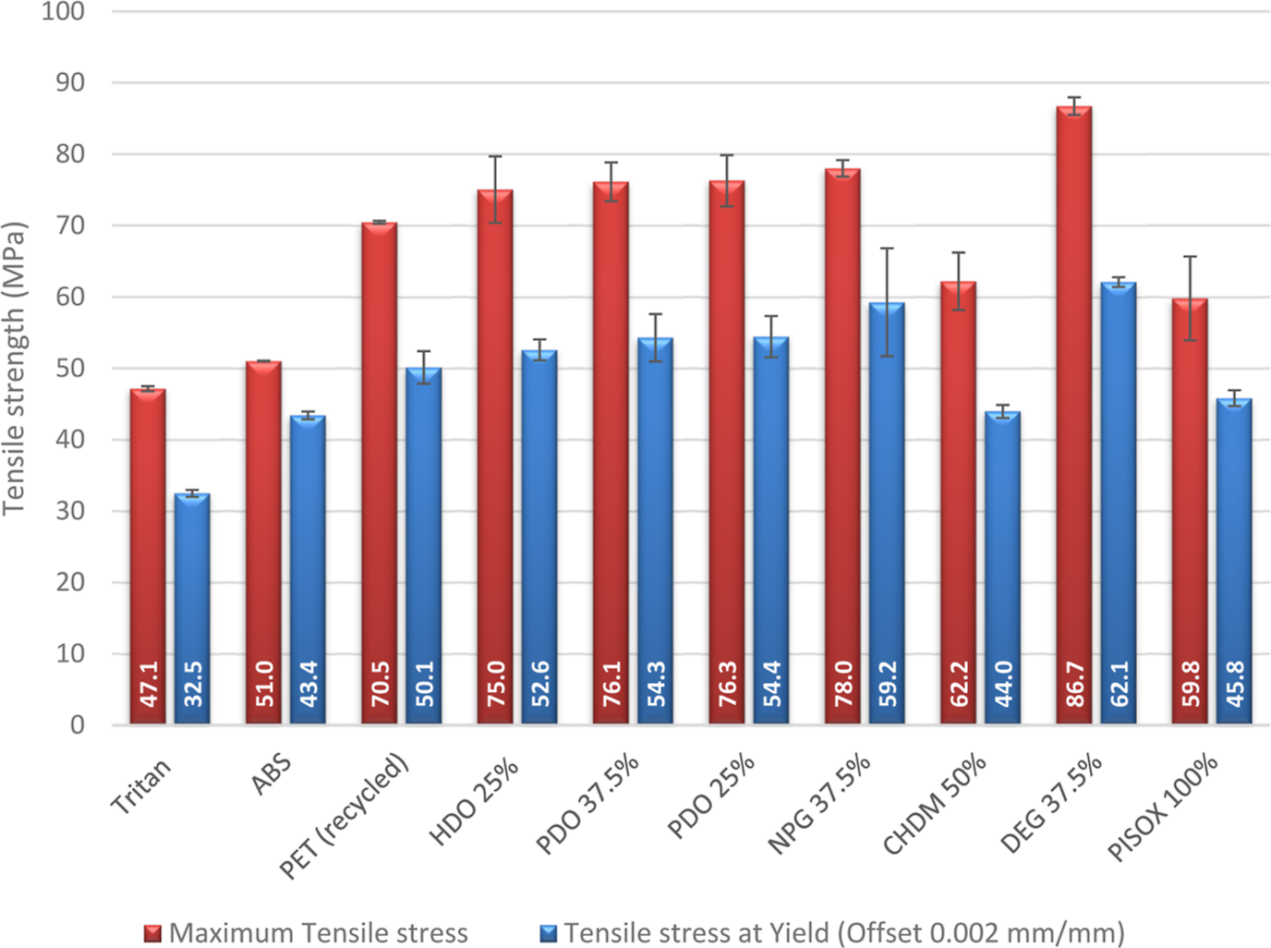
Maximum tensile stress and yield strength of the different PISOX copolymers mentioned in Table 1. Values are averages of at least 3 samples, and their standard deviation is given by the error bars. See Tables S6–S8 and Figures S50–S61 for more details.

**Figure 7:**
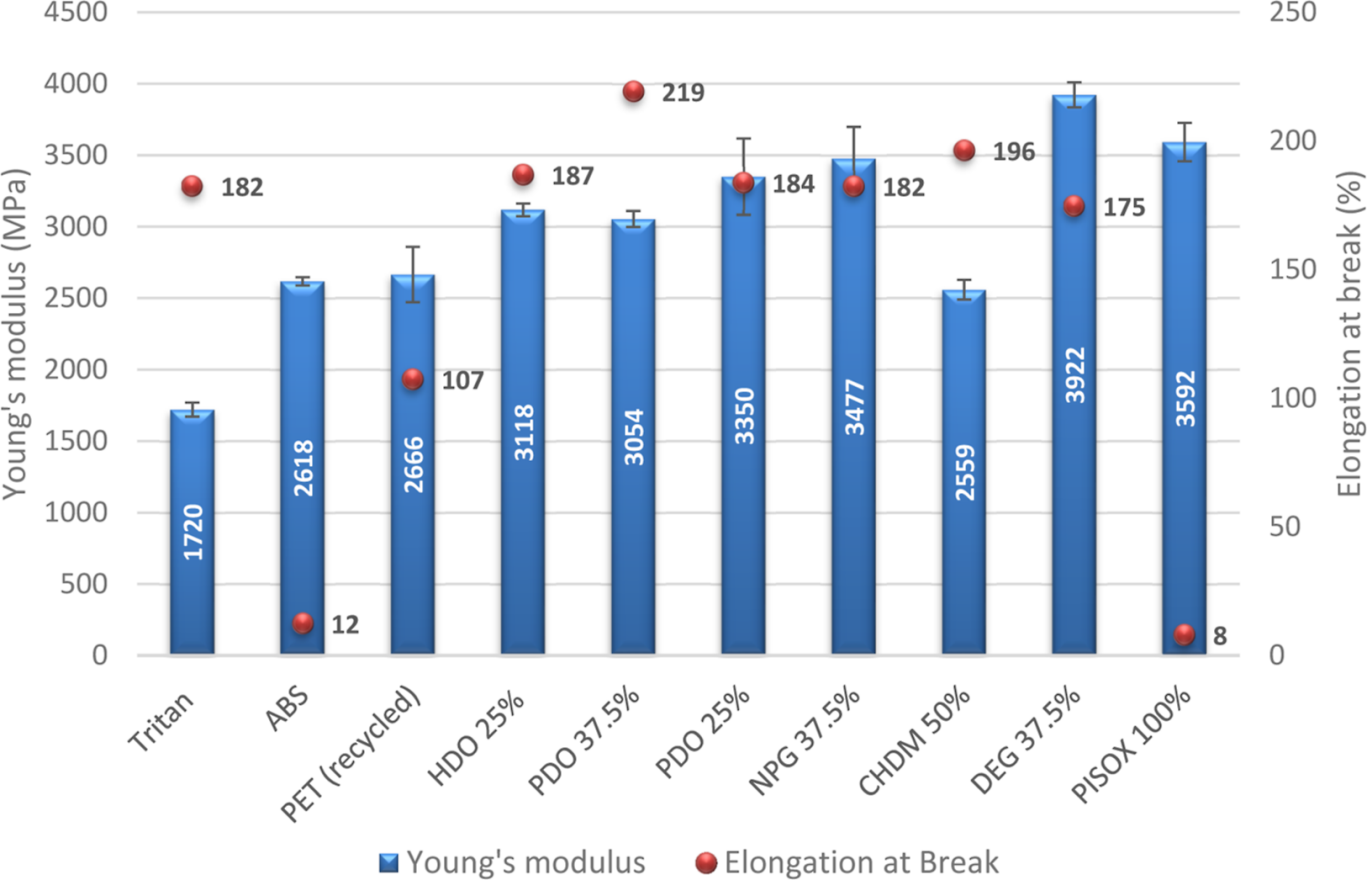
Young’s modulus and elongation at break for different PISOX copolymers mentioned in Table 1. Values are averages of at least 3 samples, and their standard deviation is given by the error bars. For the elongation at break, the maximum observed value was taken. See Tables S6–S8 and Figures S50–S61 for more details.

To see what effect the diol composition has on the mechanical properties, several PISOX copolymers were processed into tensile bars (Figure 4). These were used to measure the tensile strength, Young’s modulus, and elongation at break, shown in Figures 6 and 7. To validate the reliability of the results, commercial ABS (Terluran GP-35) was processed in a similar way as the PISOX tensile bars and compared to the provided technical data sheet. Also, commercial Eastman Tritan (copolyester TX1001) and PET tensile bars were tested and compared to their reported values. Furthermore, an external party performed the same procedure with different equipment and reported results that were in agreement with ours.

PISOX copolymers show an ultimate tensile modulus of 62.2–86.7 MPa, a yield strength of 44.0–62.1 MPa, a Young’s modulus of 2559–3922 MPa and an elongation at break of 175–219%. Overall the tensile strengths for all PISOX copolymers were quite similar, even when the chain length or content of the codiol is varied. The Young’s modulus appears to be most affected by the diol composition. As expected, when either a shorter aliphatic diol or a lower content of said aliphatic diol is used, the modulus decreases. There are only two codiols with observably different tensile strengths and modulus: CHDM and DEG. CHDM decreases the tensile modulus and strength, which is likely related to its bulkiness and flexibility. This is also observed for other reported materials that contain isosorbide and CHDM.^46,47^ While not measured in this research, CHDM likely has a positive effect on the impact resistance. Interestingly, even though it is relatively long and flexible, DEG appears to significantly increase the tensile modulus and strength, even above the value found for the homopolymer. Also for polycarbonates with isosorbide, it was found that oligo(ethylene glycol) changes the mechanical properties differently than linear aliphatic diols.^48^

Another interesting result is that the tensile strength values for the copolymers range higher than the value found for the homopolymer. For all copolymers, except the homopolymer, ductile fractures were observed with necking forces close to the yield stress and high elongation at break. This seems similar to what has been reported for poly isosorbide carbonate, the high rigidity causes the material to be brittle.^48,49^

Overall PISOX is a relatively strong and stiff material with good break resistance. When comparing the mechanical properties of PISOX copolyesters to other polymers, their values are equal or surpass those of most engineering thermoplastics, such as ABS, Tritan, and PET. The strongest copolymer, with DEG, shows tensile properties close to high-performance polymers such as PSU and PEEK.^50,51^ If we look at other isosorbide-based polymers reported in the literature, it can be seen that in general isosorbide-based polymers have a relatively high tensile strength and modulus.^7,46,47,49,52,53^ When comparing PISOX with commercial biodegradable plastics (Table 3), PISOX outcompetes these biobased plastics, both on their thermal resistance and their mechanical performance. Furthermore, PISOX is amorphous, which is not a feature of the current biobased plastics, which could be advantageous for applications where transparency or shape stability is of relevance.

**Table 3:** PISOX Mechanical and Thermal Properties Compared to Currently Most Used Commercial Biodegradable Bio-based Plastics^54,55^

	renewable content (%)	density (g/cm^3^)	tensile modulus (GPa)	tensile strength (MPa)	elongation at break (%)	*T*_g_ (°C)	M.P. (°C)
PLA	100	1.27	∼3.5	70	7	55	155–175
PBAT	0	1.22	0.085	21	670	–30	115–125
PBS (A)	30–50	1.26	0.25–0.65	24–36	170–380	∼0	84–115
PCL	0	1.15	0.3–0.4	50	400	–60	60
PHA	100	1.25	1–2	15–40	1–15	2	160–175
PISOX	70–100	∼1.4	2.5–3.9	62–87	∼200	76–167	amorph.

### Biodegradation and Hydrolysis of PISOX

Hydrolysis and biodegradation experiments on the PISOX copolymers and monomers were performed in our group in both soil and seawater.^36,56,57^ Also nonenzymatic hydrolysis of the PISOX copolyesters was studied in water. These experiments show that PISOX is significantly more degradable than cellulose in both media. In soil, it will completely degrade to CO_2_ and biomass in a matter of months and in seawater in less than a year. Compared to other biodegradable plastics, this is extremely fast, especially in the case of marine environments. This fast degradation can likely be attributed to its unique fast nonenzymatic hydrolysis under ambient conditions.

### PISOX-DEG Synthesis at Kilogram Scale

**Figure 8:**
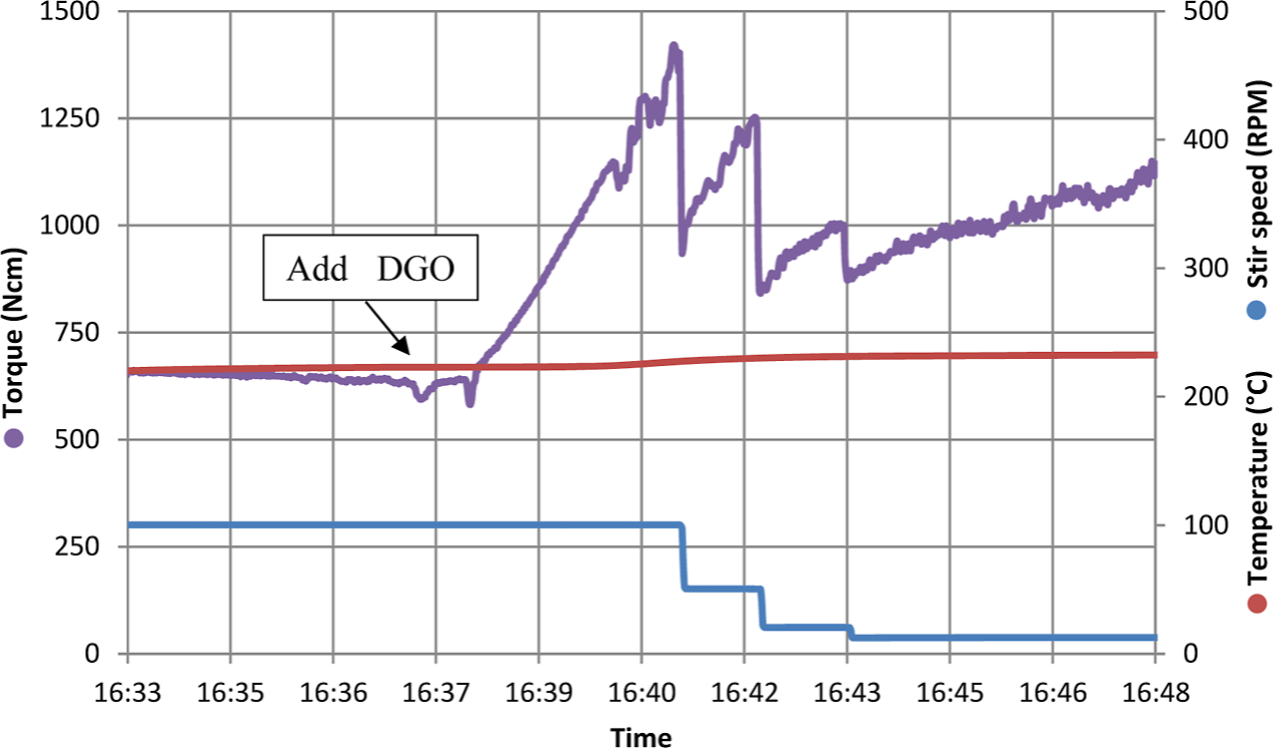
PISOX DEG 32.5% kg scale autoclave parameters (temperature, torque, and stirring speed) during a 15 min time interval during which the last addition of DGO was performed (3.0 g).

To explore the scale-up potential of PISOX, a larger-scale PISOX DEG polymerization experiment was carried out in a 2 L Buchi autoclave. Diphenyl oxalate (750 g) was used as the monomer because of its commercial availability. In the same way, as for the small-scale syntheses, the monomers were fed in equimolar ratios of total diol (67.5% isosorbide +32.5% DEG) and oxalate diester. Subsequently, the reactor was heated and stirred (75 rpm) for 3 h at 180 °C, after which vacuum was slowly applied and the temperature was raised. In the final polycondensation stage (full vacuum), no significant increase in torque was observed over time. This indicates that further polymerization had stopped. The NMR spectrum revealed that all phenyl groups had reacted and only isosorbide end groups were present. This suggests that some of the DPO had decomposed, likely by moisture in the hygroscopic monomers. To react the isosorbide end groups and continue the polymerization, multiple times DGO (30, 10, 6, 3, and 3 g; total of 7 mol %) was fed to the reactor. At the moment the last addition of DGO was added and the polycondensation was continued (see Figure 8), the high reactivity of DGO was clearly reflected in the slope of the torque increase: within less than 5 min, the torque increased from 600 to 1400 Ncm, indicating that a high molecular weight polymer was obtained rapidly. After extrusion and chipping, 425 g (74% isolated yield) of PISOX copolymer was collected. The material had a *T*_g_ of 98 °C with a DEG content of 33.7%, and a *M*_n_ of 18.7 kDa, determined by NMR.

### 3D Printing

**Figure 9:**
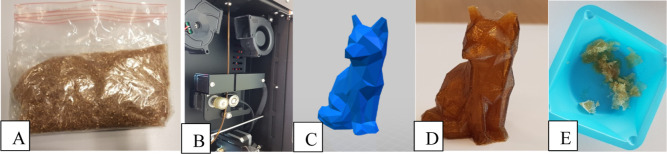
(A) 425 g of obtained PISOX resin from the autoclave. (B) PISOX filament from the 3Devo filament maker. (C) 3D model .stl file to print. (D) 3D printed “low poly fox” made from the filament. (E) Fox after 10 months (31 Aug. 2020 to 26 Jul. 2021).

The 425 g of obtained PISOX DEG copolymer was processed into a 3D printing filament using a 3Devo filament maker. The PISOX filament was used to print a 3D model “low poly fox” (Figure 9). Apart from some stringing, the print was successful and looked like the intended 3D model. An interesting feature of PISOX was observed after leaving the printed fox for almost 1 year in the open air. During this period, every now and then the fox was picked up, and after a couple of months the outer surface felt somewhat “sticky”, but the shape held firm. After about 10 months, however, the fox completely crumbled to pieces when an attempt was made to pick it up again. This clearly shows the hydrolytic instability of this polymer, which could be of interest for applications where fast degradation is desired, such as in the agricultural and biomedical sectors.

## Conclusions

Producing high-molecular-weight isosorbide-based (co)polyoxalates (PISOX) using conventional polymerization techniques is challenging. Our research presents a new approach to produce such polymers using diguaiacyl oxalate (DGO). DGO facilitates the generation of high molecular weights in short reaction periods, even in the absence of a catalyst. This exceptional reactivity of DGO can be attributed to the structural properties of oxalate (the low p*K*_a_’s of the diacid) and the good leaving ability of phenyl groups. In addition, the reverse reaction between polymer and guaiacol is extremely slow due to the steric hindrance of the *ortho*-methoxy group. Compared to currently available biobased plastics, PISOX displays superior mechanical, thermal, and barrier properties. PISOX stands out for its rapid biodegradability in both soil and seawater. It is remarkable that such a thermally and mechanically strong material degrades in a matter of months, which holds potential for specific applications.

## Data Availability

All data needed to evaluate the conclusions in the paper are present in the paper and/or the Supporting Information.

## Supporting Information Available

Additional experimental details, methods, calculations, materials, thermal data, barrier data, mechanical testing data, GPC data, and NMR spectra of polymers, monomers, and reaction stages (PDF)
