# Supplementary material for: PISOX Copolyesters—Bio- and CO2-Based Marine-Degradable High-Performance Polyesters
Source: ACS Sustain Chem Eng. 2024 Jun 18;12(26):9822–32. doi: 10.1021/acssuschemeng.4c02266 (PMC11220794; doi:10.1021/acssuschemeng.4c02266)
Supplement: Supplementary file 1 — sc4c02266_si_001.pdf [file sc4c02266_si_001.pdf]

Supporting information

PISOX copolyesters– Bio- and CO_2-based Marine Degradable High Performance Polyesters

Kevin van der Maas[1], Yue Wang[1], Daniel H. Weinland[1], Robert-Jan van Putten[1,2], Bing Wang[2], Gert-Jan M. Gruter[1,2].*

[1] Van't Hoff Institute of Molecular Sciences, University of Amsterdam; Science Park 904 1098 XH Amsterdam, The Netherlands.

[2] Avantium Chemicals BV; Zekeringstraat 29, 1014BV Amsterdam, The Netherlands.

Author e-mail addresses: k.vandermaas@uva.nl; y.wang6@uva.nl; d.h.weinland@uva.nl; robert-jan.vanputten@avantium.com; bing.wang@avantium.com

*Correspondence: g.j.m.gruter@uva.nl

Number of pages:	75
Number of figures:	64
Number of tables:	9
Number of equations:	3

Experimental
Materials

1,6-Hexanediol (99%), 1,5-propanediol (96%), neopentyl glycol (99%), diethylene glycol (99%), triethylamine (99%), titanium(IV)isopropoxide (97%), phenol (>99%) diphenyl terephthalate (98%), diphenyl carbonate (99%) and oxalyl chloride(98%) were supplied by Sigma Aldrich. Guaiacol (99%) and isosorbide (98%) were purchased from Carbosynth. Dimethyl oxalate (>99%), 1,3-propanediol (98%), cyclohexanedimethanol (99%; cis- and trans- mixture), diguaiacyl carbonate (98%) and diphenyl oxalate (98%) were bought from TCI chemicals. Tetrahydrofuran (99%), dichloromethane (99%), diethyl ether (99%), sodium bicarbonate (99.5%), sodium sulfate (99%; anhydrous), sodium chloride (>99%) were supplied by VWR International. TCE-d_2 (99.5%) and DMSO-d_6 (99.8%) were ordered from ABCR chemicals. Except for isosorbide, all chemicals were used as received. Isosorbide was purified in-house. Titanium tetra(phenolate) was synthesized according to a described patent procedure.[1] Diguaiacyl carbonate, Diphenyl terephthalate, Diphenyl Oxalate and Diphenyl carbonate were crystallized from THF before use. Diphenyl carbonate was additionally distilled before crystallization. Diguaiacyl terephthalate and Diguaiacyl oxalate were synthesized in-house.

Characterization

NMR

^{1}H-NMR and ^{13}C-NMR spectra were recorded at appropriate frequencies on a Bruker AV 300 (^{1}H, 300.10 MHz), a Bruker DRX300 (^{1}H, 300.13 MHz), a Bruker AMX 400 (^{1}H, 400.13 MHz) and a Bruker DRX 500 (^{1}H, 499.91 MHz) spectrometers. Chemicals shift are referenced to residual proton in the specified solvent.

DSC

Differential scanning calorimetry thermograms were obtained with a Mettler Toledo DSC 3 STAR^{e} system. Around 5 mg of sample was weighed in a standard aluminum crucible (40 µl). Next, the sample was analyzed in three steps under a nitrogen flow of 50 ml*min^{-1}. First, after stabilizing at 20 °C for 5 minutes, the sample was analyzed at a rate of 10 °C*min^{-1} from 20–230 °C. Second, the sample was cooled down to the starting temperature of 20 °C with a cooling rate of 50 °C*min^{-1}. Lastly, the first step is repeated, and the data of this cycle is used for reporting.

TGA

Thermogravimetric analyses were obtained by a Mettler Toledo TGA/DSC 3 STAR^{e} system. Around 15 mg of sample was weighed in an aluminum crucible (100 µl). Next the sample was analyzed at a heating rate of 10 °C*min^{-1} from 20–550 °C under a nitrogen flow of 50 ml*min^{-1}.

GPC

For all copolymers - Molecular mass distributions were measured using size exclusion chromatography (SEC) on a Shimadzu LC-20AD system with two PLgel 5 μm MIXED-C columns (Polymer Laboratories) in series and a Shimadzu RID-10A refractive index detector, using dichloromethane as mobile phase at 1 mL/min and T = 35 °C.

For PISOX-100% GPC measurements were carried out on a Hitachi Chromaster 5450 with a Agilent HPLC system equipped with two PFG 7 micrometer (μm) Linear M (300×7.5 mm) columns. HFIP was used as mobile phase with a 1mL/min flow and T = 35 °C. Calculation of the molecular weights were carried out with Astra 6 Software.

Procedure compression molding films

The films used for the barrier measurements were prepared by compression molding with the help of a thermal press (Carver Auto Four/3015-NE,H). Granulates of PISOX are dried in a vacuum oven overnight at 60 °C and 2 mbar. A press shape (20*20 cm) is prepared by folding a long piece of aluminum foil 3 times to get 8 layers thick aluminum foil. Half a circle with a diameter of 10 cm is cut out of the foil. The foil is then folded open once to get a circle with a diameter of 10 cm and 4 layers thickness (~0.1 mm). The aluminum foil press shape is then pre-pressed (10 Force tons) in between two sheets of glass fiber reinforced PTFE (20*20*0.14 cm) and two aluminum plates (20*20*3 cm). The sandwich is opened and 1.5 grams of polymer is transferred to the middle of the press shape. The polymer is pre-molten by placing the sandwich in the hot press at 190 °C for 2 minutes without pressing. Then the sandwich is pressed at 0.5 tons for 1 minute, 1 tons for 30 seconds, 2 tons for 30 seconds, 5 tons for 30 seconds, and 10 tons for 30 seconds. The sandwich is then removed from the press. The Teflon sheet with the polymer

and press shape is separated from the sandwich and left to cool at a flat cold surface. When cooled down the pressing shape and PTFE sheets are removed to obtain the PISOX film (~100 μm). The thickness was measured with a digital caliper (accuracy of 5 μm). Multiple films were made and visually examined for bubbles and defects. The best films were selected for barrier measurements. The thickness of the films ranged from 100 to 120 μm.

For the PET film: a similar procedure was used, except that a higher hot-press temperature was used of 275 °C and the resulting film was cooled down on a cold aluminum plate to prevent crystallization. The resulting film had a 10% degree of crystallization according to DSC analysis.

Barrier measurements

Oxygen and water barrier measurements were performed on a Totalperm (Permtech s.r.l) instrument. Calibration of the system was carried out with a standard PET film provided by Permtech (Italy), according to the ASTM F1927-14 standard. Oxygen measurements were performed at 30 °C, 0% humidity and 30 °C, 50% humidity. Water vapor measurements were performed at 38 °C, 90% humidity.

Injection molding

Tensile bars were obtained with a Thermo Scientific HAAKE Minijet II apparatus equipped with an ISO-527-2-A5 mold. The pressure was set at 1000 bar, mold temperature at 50 °C and the pressure time was set at 15 seconds. The cylinder temperature varied for each copolymer: HDO-25% (245 °C); CHDM-50% (235 °C); PrDO-25% (210 °C); NPG-37.5% (225 °C); PrDO-37.5% (190 °C); DEG-37.5% (190 °C); PISOX-100% (260 °C). When temperature and pressure was stable, the cylinder was checked to make sure no residues from previous runs remained. Before

each press, the mold was coated with water-based silicon mold release agent, and when dry, placed in the holder. Next 2.2-2.7 grams of polymer was weighed and transferred inside the cylinder. The polymer was left to melt for 120 seconds inside the cylinder. Subsequently, the cylinder was placed on top of the mold, the door was closed, and the injection program was started. After the injection program, the mold was taken apart and the sample was removed and examined for defects. The excess polymer in the cylinder was discharged before each injection run. A typical PISOX tensile bar (ISO-527-2-A5) weighed 1.8 gram.

Density measurements

The Thermo Scientific HAAKE Minijet II was equipped with a 25x1.5 mm rheometer probe disk mold. The same press settings as for the tensile bars were used. The weight and volume of the disks were used to determine the density of the material.

Tensile testing

The tensile bars were analyzed on an Instron 5565 machine with load cell (10 kN) and Instron strain gauge extensometer 2630-106 (25 mm). Sample size were set to width (4 mm), thickness (1.95 mm) and parallel length (25 mm). The tensile tests were performed at a test speed of 5 mm/s or 50 mm/s. When the maximum elongation of the extensometer was reached (100%) the extension of the frame was used to determine the elongation at break.

Filament making

The filament was made on a Precision 350 filament maker from 3Devo. The filament maker has a 4-zone heated extruder screw with exchangeable nozzle size. Only a couple of hundred grams of resin is required for filament processing. The drawn filament is air cooled, measured in

diameter and coupled to a spool winding system. The process of spooling and keeping a consistent diameter is controlled by the machine. The following settings were used: Heater 1 (135 °C), 2 (140 °C), 3 (140 °C), 4 (135 °C), screw speed (3.5 RPM), fan speed 15% and filament diameter 2.85 mm.

3D printing

The 3D models were printed on an Ultimaker 3 Extended. The build plate was prepared by coating with a glue stick. The following settings were used for PISOX-35% DEG: Heating plate (60 °C), Nozzle (185 °C), infill (20%), print speed (60 mm/s), fan speed (100%) and layer height (0.2 mm).

Equations

Calculations equilibrium constant and molecular weight

Below (**Figure S1.**) you can find the general transesterification reaction of isosorbide or isosorbide end groups with phenyl oxalate or phenyl oxalate end groups. [A] represents the concentration of phenyl ester groups, [B] the isosorbide hydroxyl group, [C] the isosorbide oxalate ester and [P] the phenol leaving group.

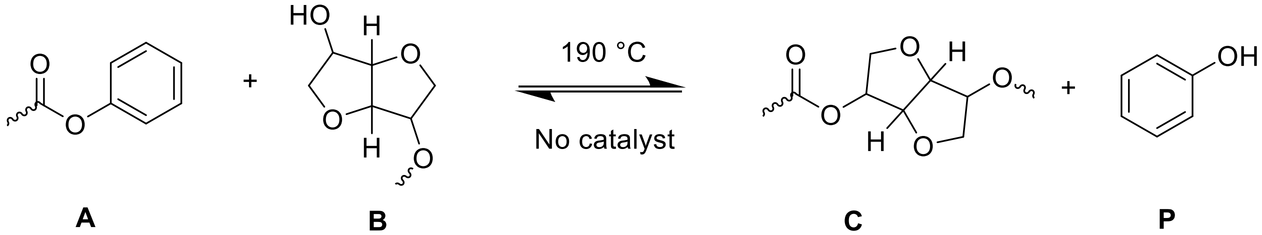

Figure S1. Equilibrium reaction of isosorbide (end groups) with phenyl oxalate (end groups).

Since the reactants are in a closed system and added in equimolar amounts, [A] = [B] = $[A]_0$ - [P] and [P] = [C] at any time, with $[A]_0$ being the initial phenyl end group concentration. Therefore, the following formula (**Equation 1**) can be used to calculate the equilibrium constant:

$$K_{eq} = \frac{[C] * [P]}{[A] * [B]} = \frac{[P]^2}{[A_0 - P]^2}$$

Equation 1. Calculation of the equilibrium constant.

Subsequently, the following formula (**Equation 2**) can be used to calculate the average degree of polymerization ($\bar{X}_n$):

$$\bar{X}_n = \frac{1}{1 - P}$$

Equation 2. Calculation of the average degree of polymerization.

The average degree of polymerization can then be used to calculate the average molecular weight (M_n), see **Equation 3**. With $M_w(PISOX)$ representing the molecular weight of the PISOX repeating unit (200 g/mol) and $M_w(Aryl)$ the molecular weight of the used aryl group (124 g/mol for guaiacyl and 94 g/mol for phenyl)

$$M_n = \bar{X}_n * 0.5 * M_w(PISOX) + M_w(Aryl)$$

Equation 3. Calculation of the average molecular weight.

Calculations molecular weight by ^{1}H-NMR

To calculate the molecular weight (M_n) by ^{1}H-NMR, the content of the monomers and the repeating length should be determined. See **Figure S2** for an example.

Monomer content – The monomer content can be determined by the integrals of the monomers. In our example we use the 1,6-hexanediol copolymer (of course, molecular weight, and integral signals changes accordingly to the type copolymer). The integral of 4.75 – 5.10 ppm is taken for isosorbide, 1 proton. The integral of 1.35 – 1.55 ppm is taken for hexanediol, 4 protons. The sum of both diols is taken as 100%. The oxalate is not directly visible in ^{1}H-NMR, however in the case of a polymer, it should be present in equal amounts as the diols.

Repeating length – To calculate the repeating length of the polymer chain, the integrals of the endgroups have to be used. The end groups which are most likely present are: formate (1H, 8.0 - 8.25 ppm), guaiacyl (4H, 7.4 - 6.9 ppm), isosorbide (1H, 3.5 - 3.7 ppm) and hexandiol (4H, 1.35 - 1.25 ppm). In our case we take the integral of isosorbide (1H, 4.75 – 5.10 ppm) as 100%. Since each polymer chain requires two end groups to form a chain, we can calculate the amount of isosorbide repeating units as following: $Iso_{Rep} = \frac{2}{\sum endgroups} * 100$. We can now find the other repeating unit length according to the monomer contents determined before.

Molecular weight calculation – To calculate the molecular weight (M_n), the average weight of the polymer chain should be determined and added up to the average molecular weight of the endgroups: $Mn_{polymer} = Mn_{end\ groups} + Mn_{polymer\ chain}$. To determine the molecular weight of the polymer chain, each repeating unit should be multiplied by their molecular weight and added up: $Mn_{polymer\ chain} = 56.03 * OX_{Rep} + 116.2 * HDO_{Rep} + 144.14 * Iso_{Rep}$. In our case

we used 56.03 g/mol for each oxalate repeating unit, 116.2 g/mol for hexanediol and 144.14 g/mol for isosorbide. To calculate the average molecular weight of the endgroups, each individual endgroup should be expressed as percentage of total end groups, this should then be multiplied by its molecular weight and normalized by the total endgroups present, namely two. The formula can be expressed as following: $Mn_{end\ groups} = 29 * \frac{Formate_{end}}{\sum endgroups} * 2 + 123.1 * \frac{Guaiacyl_{end}}{\sum endgroups} * 2 + 145.1 * \frac{isosorbide_{end}}{\sum endgroups} * 2 + 117.2 * \frac{HDO_{end}}{\sum endgroups} * 2$.

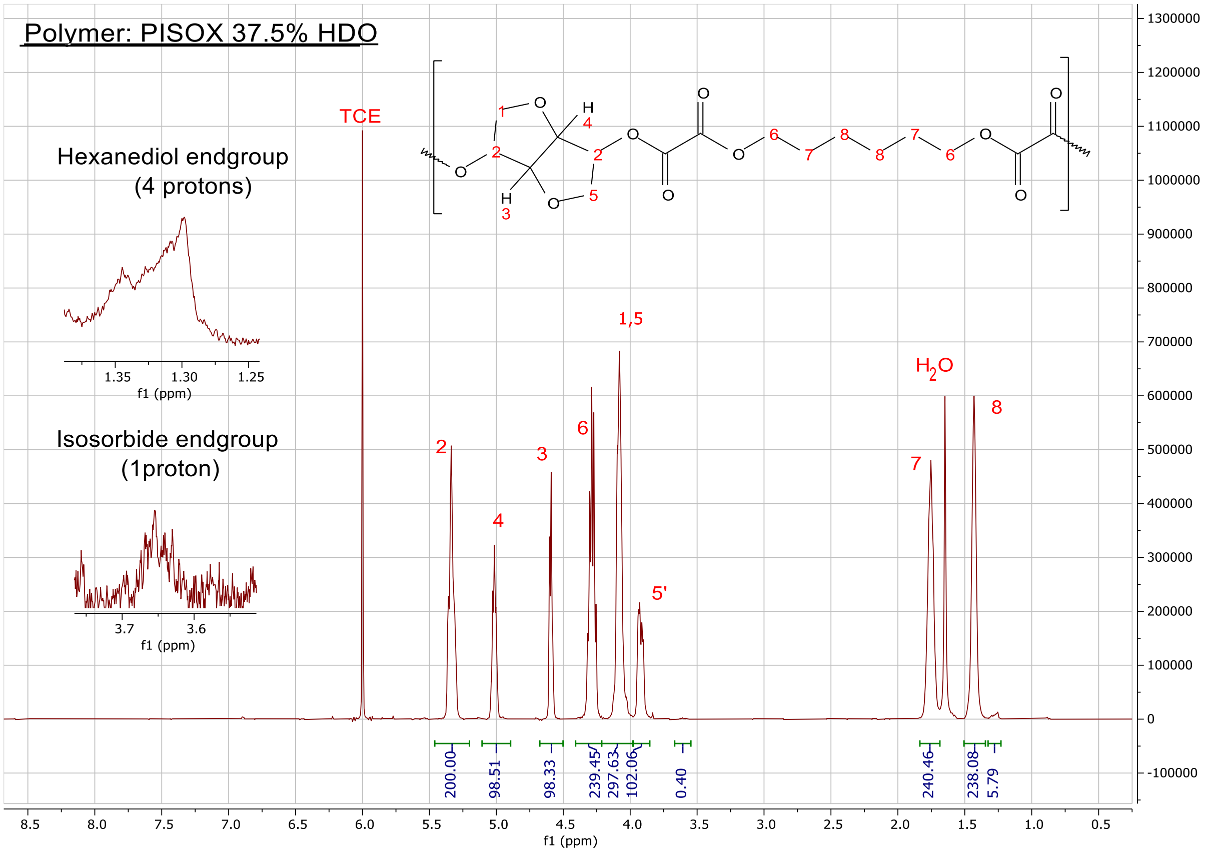

Figure S2. Example of NMR molecular weight calculation of PISOX 37.5% HDO. Important to add is that at these high molecular weights calculations based on NMR have a significant standard deviation, as the signal of the end group is rather close to the sensitivity limit of the NMR. Also GPC molecular weight calculations of polyester based on polystyrene standard are known to deviate from the real value. Both of these factors probably cause the molecular weight deviation between the two analysis methods.

T_g prediction with Fox Equation

The Fox Equation: $\frac{1}{T_g} = \frac{m_1}{T_{g,1}} + \frac{m_2}{T_{g,2}}$ was used as a model to predict the T_g of the different PISOX codiols. In the **Table S1** below you can find the T_g parameters either found in this work or literature. In **Figures S3** you can find the experimental and theoretical plot of this data.

Table S1. T_g values of the homopolymers found in literature.

	T_g literature	Source
Poly(hexylene oxalate)	-35	[2]
Poly(propylene oxalate)	-34	[3]
Poly(neopentyl oxalate)	7	[3]
Poly(butylene oxalate)	-18	[4]
Poly (isosorbide oxalate)	167	This work

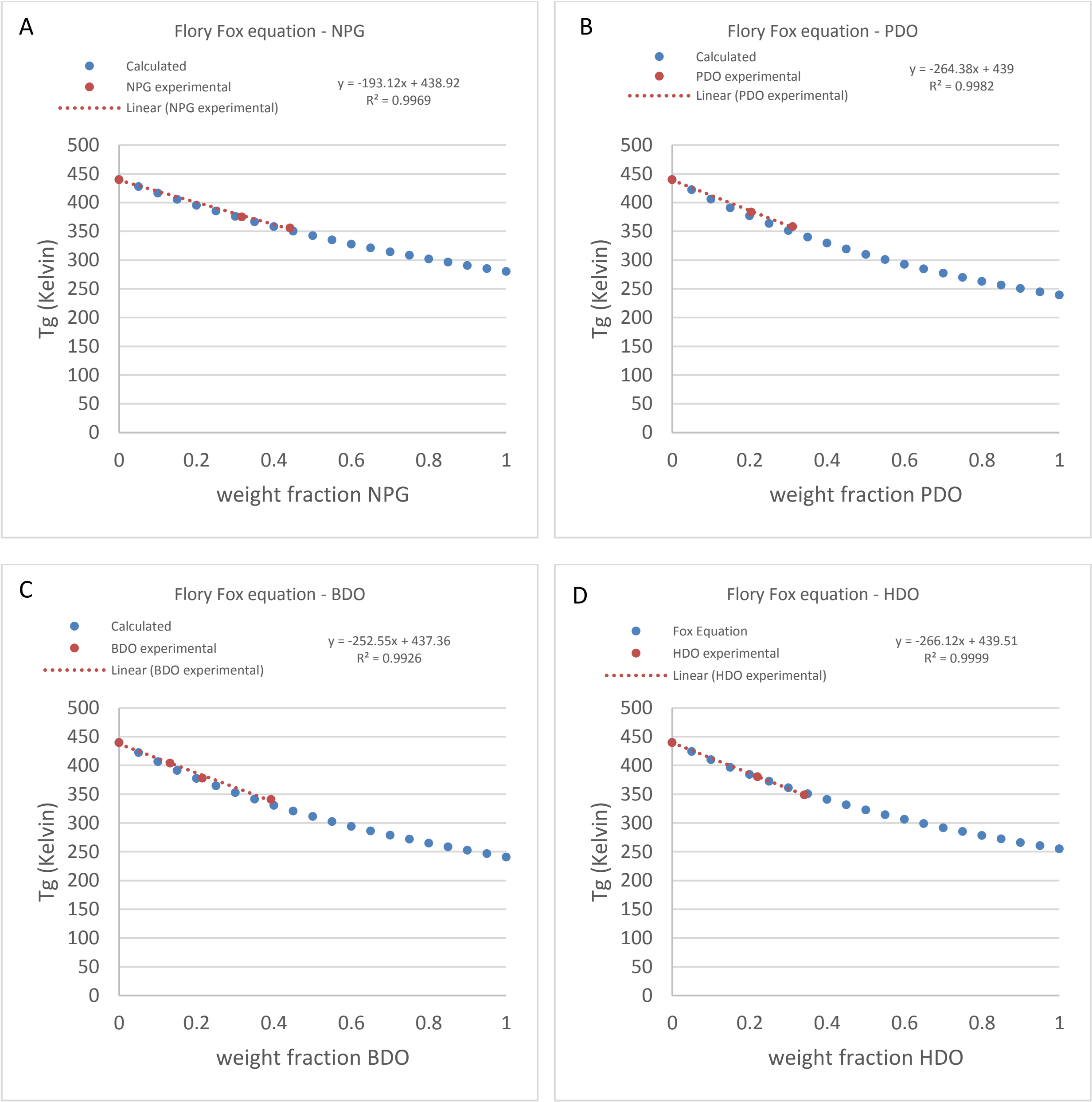

Figures S3. Fox equation prediction and experimental data; (A) NPG copolymer (B) PDO copolymer (C) BDO copolymer (D) HDO copolymer. Within this experimental window for all copolymers, a linear relationship with the weight fraction seems to fit the experimental data.

Calculations of the different barrier properties

To be able to compare the experimentally obtained barrier data to barrier data from literature, the oxygen transmission rate (OTR) has to be converted to oxygen permeability (OP) and the water vapor transmission rate (WVTR) to water vapor permeability (WVP). The equations and units for these calculations are given in **Table S2**.

Table S2. Calculations of the different barrier properties. Oxygen partial pressure difference in our experiments is [100%] (1 atm) and partial water pressure in our measurements (38 °C and 90% RH) is 6.633 kPa * 0.9 = 6 kPa

Barrier property	Equation	Unit
Water Vapor Transmission Rate (WVTR)	$WVTR = \frac{Weight\ passed\ through}{area \cdot time}$	$g/m^2 \cdot day$
Water Vapor Permeability (WVP)	$WVP = \frac{WVTR \cdot Thickness}{saturated\ pressure \cdot \Delta\%RH}$	$g \cdot \mu m/m^2 \cdot day \cdot kPa$
Oxygen Transmission Rate (OTR)	$OTR = \frac{Volume\ passed\ through}{area \cdot time}$	$cm^3/m^2 \cdot day$
Oxygen Permeability (OP)	$OP = \frac{OTR \cdot Thickness}{Oxygen\ partial\ pressure\ difference}$	$cm^3 \cdot \mu m/m^2 \cdot day \cdot atm$

Diguaiacyl terephthalate from terephthaloyl chloride

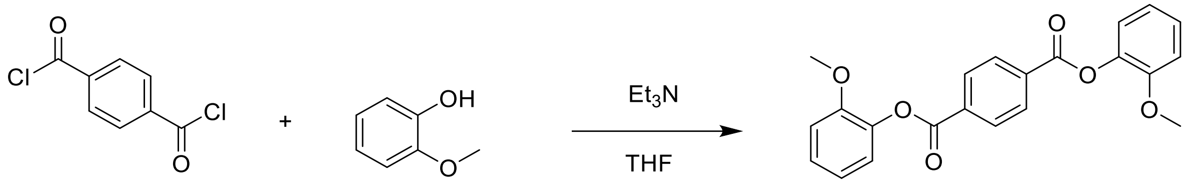

terephthaloyl dichloride Guaiacol bis(2-methoxyphenyl) terephthalate

62.7 g of guaiacol (505 mmol; 2.05 eq) and 52.3 g of triethylamine (517 mmol; 2.1 eq) was dissolved in 600 mL of THF and transferred to a 3 neck round bottom flask equipped with mechanical stirrer (PTFE), temperature probe and pressure equalizing dropping funnel. Next, a solution of terephthaloyl chloride 50 g (246 mmol; 1 eq) in 200 mL THF was made and was transferred to the dropping funnel. The stir speed was set at 150 RPM and the terephthaloyl chloride solution was added dropwise over the course of 30 minutes while the temperature was kept below 50 °C. After the addition, the reaction was heated by a water bath to 50 °C for 10 minutes. To remove most of the $Et_3N.HCl$, the warm solution was filtered by vacuum glass filtration. The glass filter residue was rinsed twice with 250 mL warm THF. The THF of the filtrate was evaporated under reduced pressure. The obtained crystals were dissolved in 750 mL DCM. The DCM was washed with $NaHCO_3$ (2x 500 mL), H_2O (500 ml) and brine (500 mL), followed by drying over $NaSO_4$. The DCM was then evaporated under reduced pressure. The resulting crude product was dissolved in hot THF (750 mL) and left to cool down slowly overnight (fridge). The formed crystals were separated by glass vacuum filtration, and washed twice with 50 mL diethyl ether. 75 g (80% yield) of fine white crystals were collected. ^{1}H-NMR analysis confirmed we obtained a pure product (**Figure S4**).

Synthesis of bis(2-methoxyphenyl) oxalate by oxalyl chloride

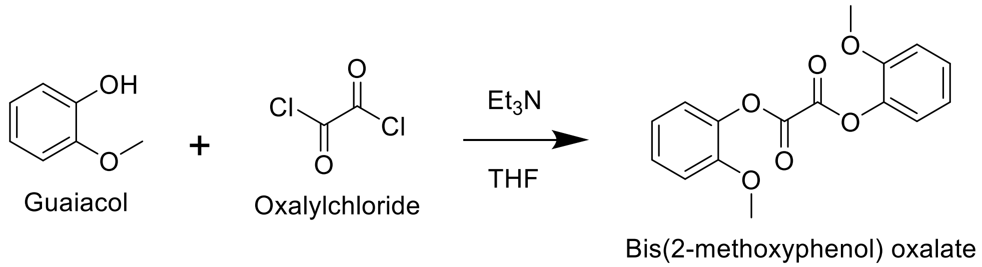

To a solution of guaiacol (600.3 g, 4.84 mol) and triethylamine (544.4g, 5.38 mol) in THF (3.0 L) cooled in an ice bath and stirred with an overhead stirrer, oxalyl chloride (304.1 g, 2.40 mol) in THF (1.25 L) was added dropwise while keeping the reaction temperature below 10 °C. After addition, the reaction was stirred for another 2 hours. To check completion a sample was taken and analyzed by ^{1}H-NMR: the shift of the methoxy group from 3.75 ppm to 3.84 ppm (DMSO-d6) confirms completion. Typically, the reaction was finished within 1 hour after addition.

When complete, the reaction mixture was filtered to remove most of the $Et_3N \cdot HCl$. The residue was washed twice with THF (2.0 L). The organic phases were combined and the THF was evaporated under reduced pressure on a rotavapor. Next, the product was dissolved in DCM (2.0 L), washed 1x with sat. $NaHCO_3$ (1 L), 2x with H_2O (1 L), 1x brine (1 L) and dried over anhydrous Na_2SO_4. The remaining (yellowish) crystals were dissolved in THF (0.75 L) at 70°C. When all dissolved, the solution was left to cool to room temperature to crystallize overnight. The next day the solution was put in the fridge (7 °C) to continue crystallization. The obtained crystals were filtered off and washed 3x with cold diethyl ether (0.5 L). The fine white crystals were dried under reduced pressure at 60 °C. The obtained product (518 g, 0.44 mol; 73% yield) was analyzed by ^{1}H-NMR (**Figure S5** to **Figure S9**) and DSC/TGA (**Figure S10** and **Figure S11**). The melting point of the product was 126.5°C (>99.9 mol% by DSC purity analysis).

Synthesis DGO - Transesterification of dimethyl oxalate with Guaiacol

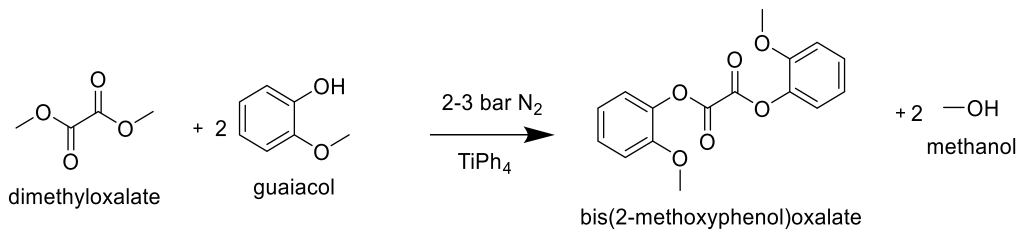

1192 g guaiacol (9.6 mol; 2.94 eq), 386 g dimethyloxalate (3.27 mmol; 1 eq) and 2.9 g titanium tetra(phenolate) (5 mmol; 1.7 meq) were transferred to a steel 2 L kiloclave (Buchi). The reactor pressure was set to 3 bar with a N2 bleed of 2 L/h. The heater oil temperature was set to 275 °C (245 °C internal), when the reactor temperature reached 100 °C the stirrer was started and gradually increased to 100 RPM. The reaction was followed by removing and monitoring the products in the condensation flask. After 4 hours the pressure was gradually lowered to 2 bar, and the reaction was continued for 2 hours. Subsequently, the oil temperature was set to 250 °C (225 °C internal) and the pressure was gradually lowered to atmospheric pressure to facilitate the distillation of guaiacol. When distillation came to a halt, the pressure was lowered further by using a vacuum pump. When a pressure of 10 mbar was reached, the condensation flask was drained to make room for the DGO product. Next, full vacuum was applied (<0.1 mbar) at an oil temperature of 260 °C to facilitate the distillation of the DGO product into the condensation flask. The condensation product (300 g ; 30% yield) was dissolved in THF (0.5 L), and left to crystallize overnight. The obtained crystals were filtered off and washed twice with (0.2 L) diethyl ether. The obtained product (225g, 22.5% yield) was analyzed by ^{1}H-NMR (**Figure S5** to **Figure S9**) and DSC (**Figure S12**). The melting point of the product was 126.2 °C (>99.8 mol% by DSC purity analysis).

Isosorbide purification

The commercial isosorbide gave a brittle and strongly colored polymer, indicating bad quality. High purity isosorbide is required for polymerizations. However, despite being commercially produced, isosorbide of sufficient purity was not readily available. Therefore the commercial isosorbide had to be purified. First the isosorbide was crystallized from acetone, followed by a distillation step over sodium borohydride. The purity was assessed by DSC/TGA analysis (>99.5%; **Figure S13** and **Figure S14**) and ^1H NMR (**Figure S15**). However, a better lead for assessing the quality of the isosorbide was the polymerization itself. After the crystallization step the color and quality of the polymer improved significantly and was good enough to obtain high molecular weight. Doing a distillation step over sodium borohydride, was found to be beneficial to improve the color of the final polymer (**Figure S16**).

1.6 Kg of isosorbide (Carbosynth) was dissolved in hot acetone (600mL). The slightly yellow transparent solution was then transferred to a 2 L 1-neck flask. The solution was left to cool at R.T. and left overnight to crystallize. The next day, the solution was placed in a freezer (-20 °C) for 2 more days to continue crystallizing. After crystallization, the leftover liquid was decanted off and the crystals were washed twice with ethyl acetate (300 mL). At this stage there was 1257g (78.6%) of isosorbide left in the flask. Next, 7 g 0.5% (w/w) of $NaBH_4$ was added. A long path distillation was used. The flask was slowly heated in an oil bath (100 °C), followed by slowly applying vacuum. Since gases are formed by the $NaBH_4$, which causes the solution to expand, temperature was gradually increased to 175 °C. Isosorbide distilled over at 175 °C oil temperature and 0.3 mbar. The received isosorbide had a faint yellow glow as a liquid, but as a solid was completely white. The yield was 874 g (55%). The residue (383 g) had a dark brown color.

Synthesis of PISOX (co)polymers

In a typical polymerization, a 100 mL three neck round bottom flask was charged with equimolar amounts of DGO and Diols. The round bottom flask was put in an oil bath and equipped with a mechanical stirrer, nitrogen inlet and short path connected Schlenk flask, all suited for high vacuum. Nitrogen flow was set to 50 mL/min and the temperature of the oil bath was set to 175-190 °C. The set temperature was reached in about 30 minutes. As soon as a homogeneous melt was observed the stirring speed was set to 100 RPM. Next, the polymerizations were carried out in two stages. In the first stage the reaction mixture was stirred under a nitrogen flow at a temperature of 175-190 °C for 3 hours. In the second stage the condensation product, guaiacol is removed by applying vacuum and increasing the temperature. Typically, the free guaiacol is removed over the course of 1 hour, depending on the scale. Finally, full vacuum (<1mbar) is applied for 1 hour at the melting temperature of the polymer, typically around 200 - 240 °C.

Equilibrium reactions

10 mmol of diaryl carboxylate and 10 mmol of isosorbide were weighed in a glass vial (7.5 mL) and a magnetic stir bar was added. Next, the glass vials were placed in a temperature controlled aluminum block, with individual slots for the glass vials. Roughly 2/3rd of the glass was in contact with the aluminum heating element. The vials were closed off with a plastic cap, and were pierced with a tiny needle. The temperature of the block was set to 190 °C (~185 °C inside sample vial). Time was started as soon as the block reached the set temperature (~15 min).

Characterization in-house made diguaiacyl terephthalate (DPT)

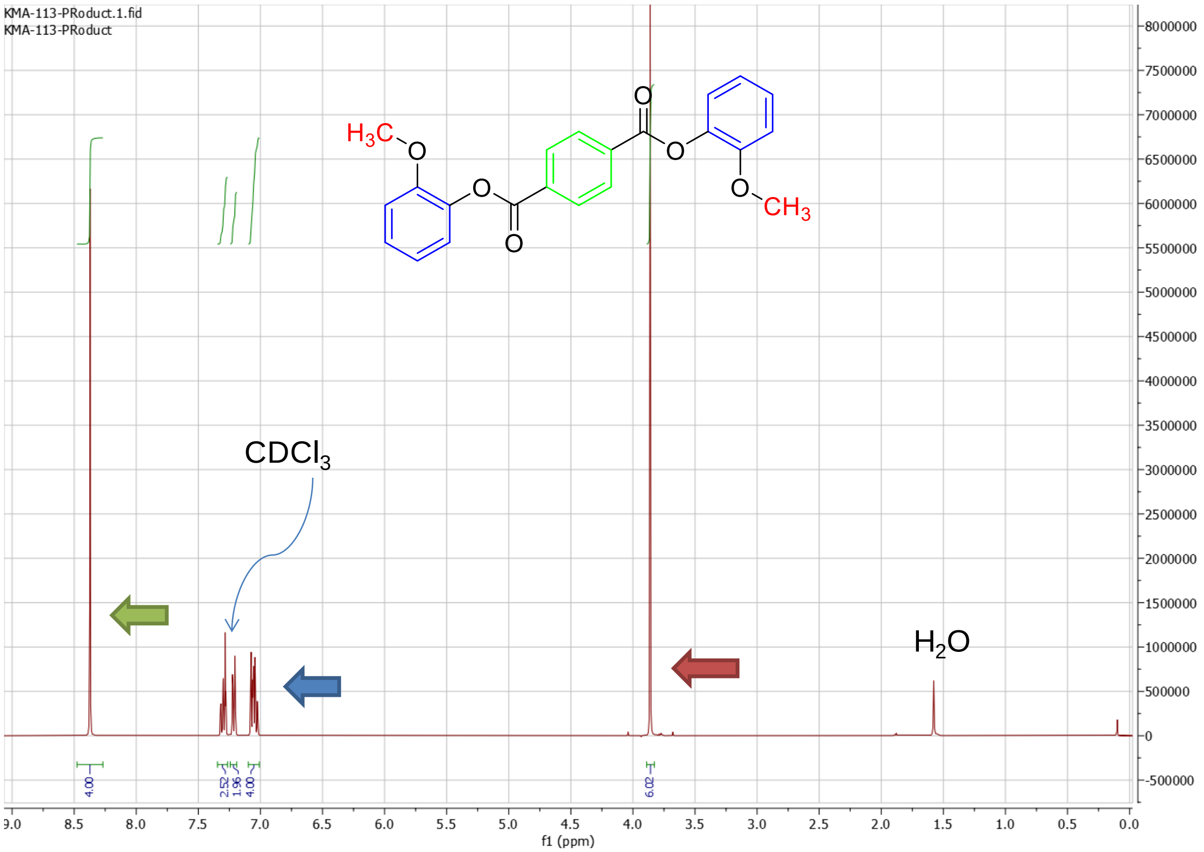

Figure S4. ^{1}H-NMR of Diguaiacyl terephthalate in $CDCl_3$.

Characterization of bis(2-methoxyphenol)oxalate (DGO)

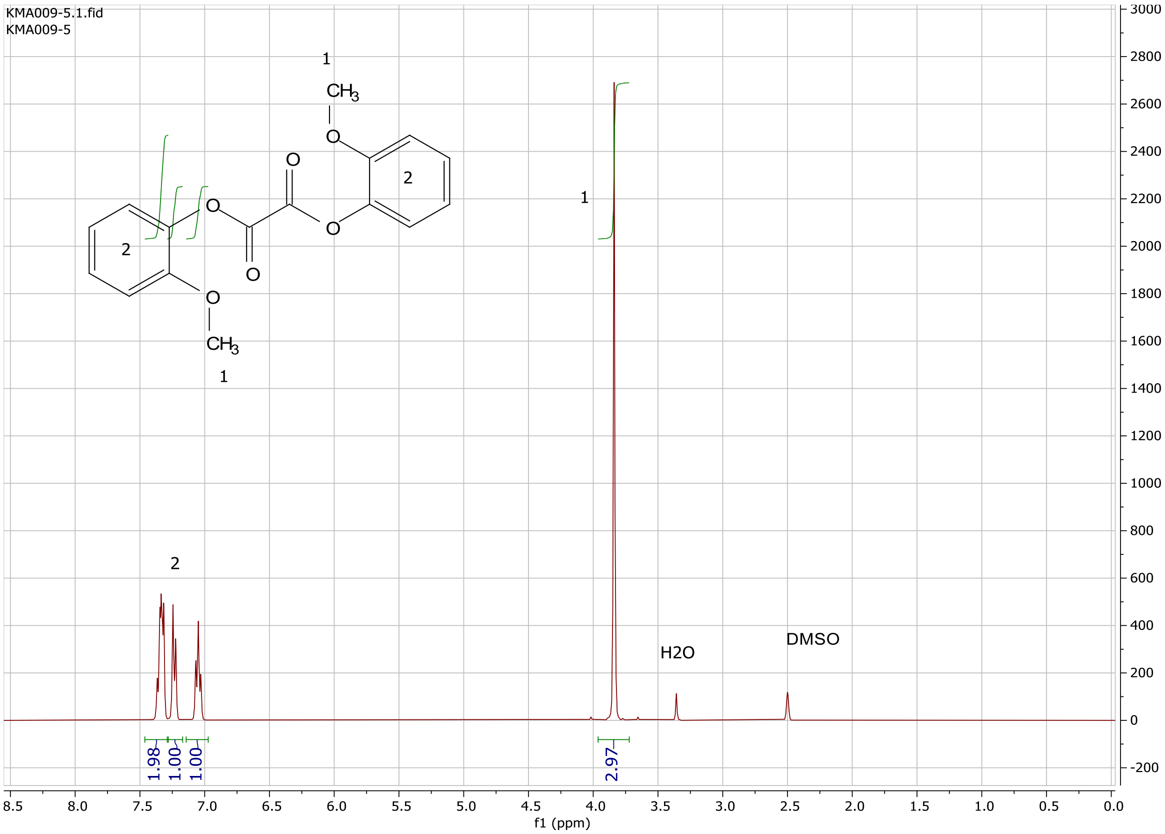

Figure S5. ^{1}H NMR of DGO in DMSO

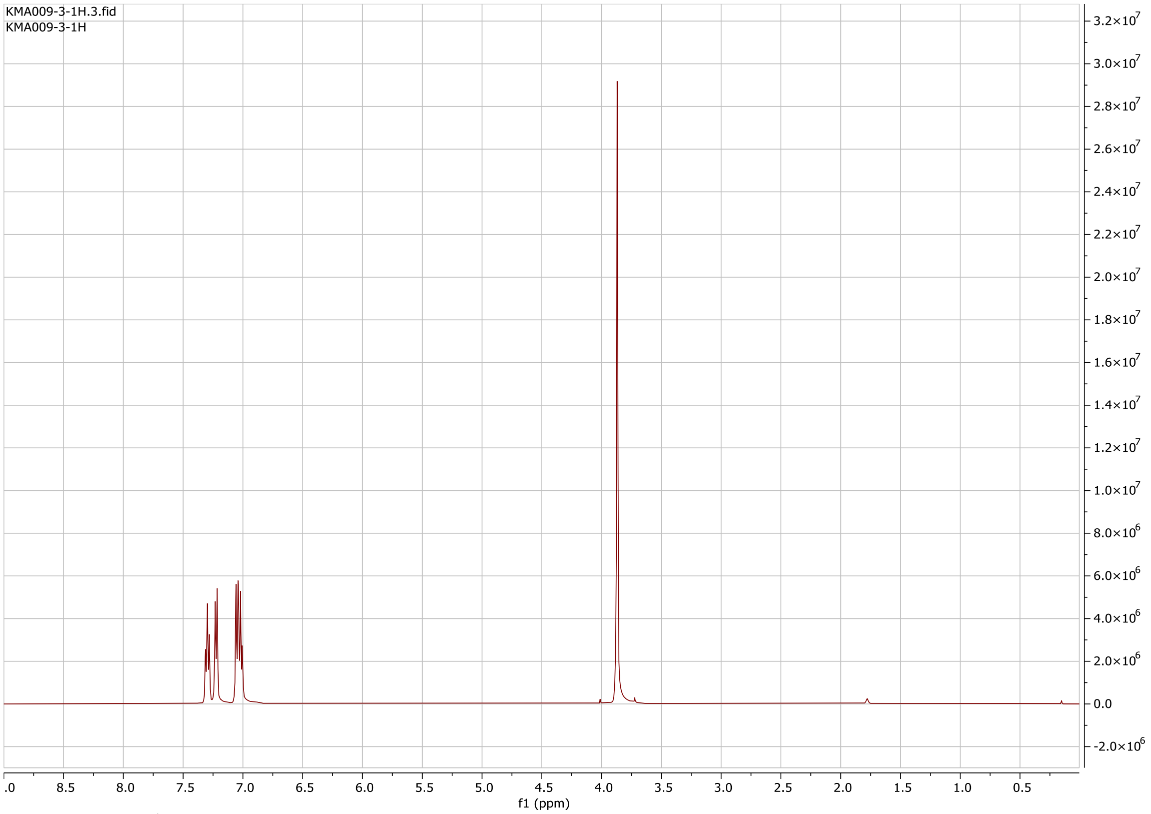

Figure S6. ^{1}H NMR of DGO in CDCl3. Note the signal of $CDCl_3$ falls into the product signals making reference assignment of the solvent signal difficult.

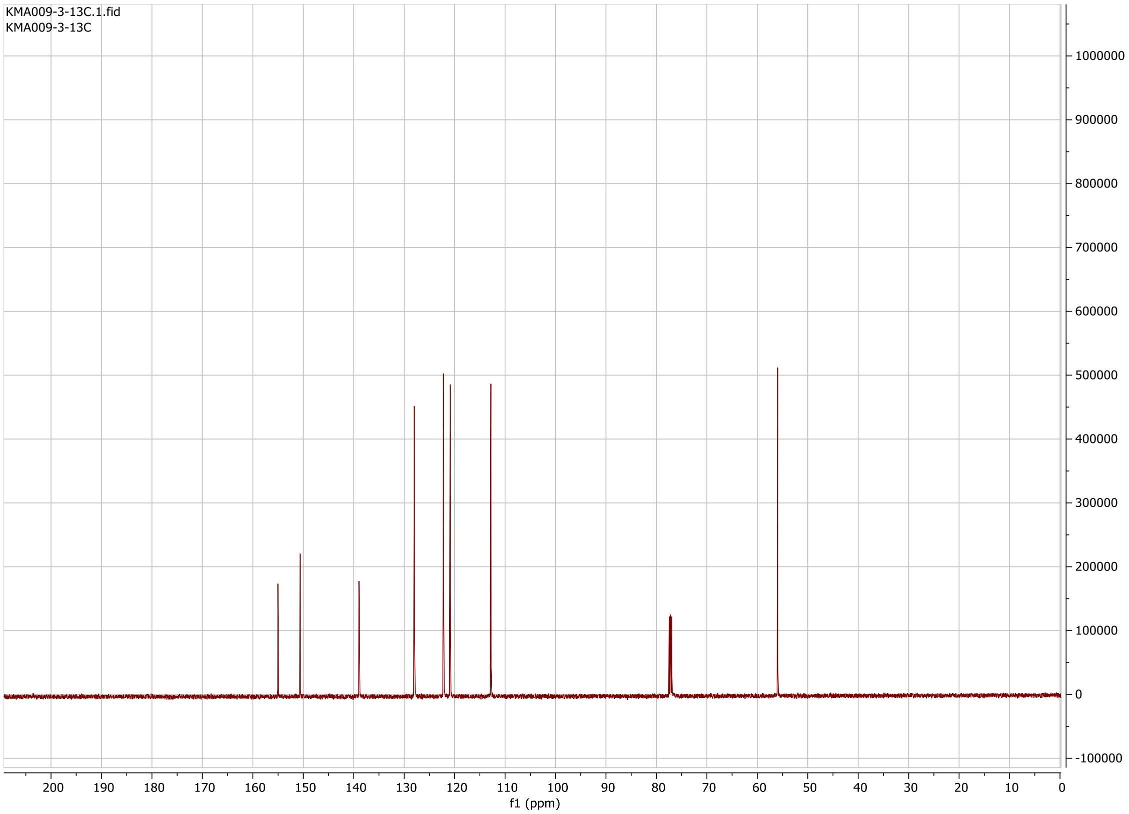

Figure S7. ^{13}C-NMR (500MHz) of DGO in $CDCl_3$.

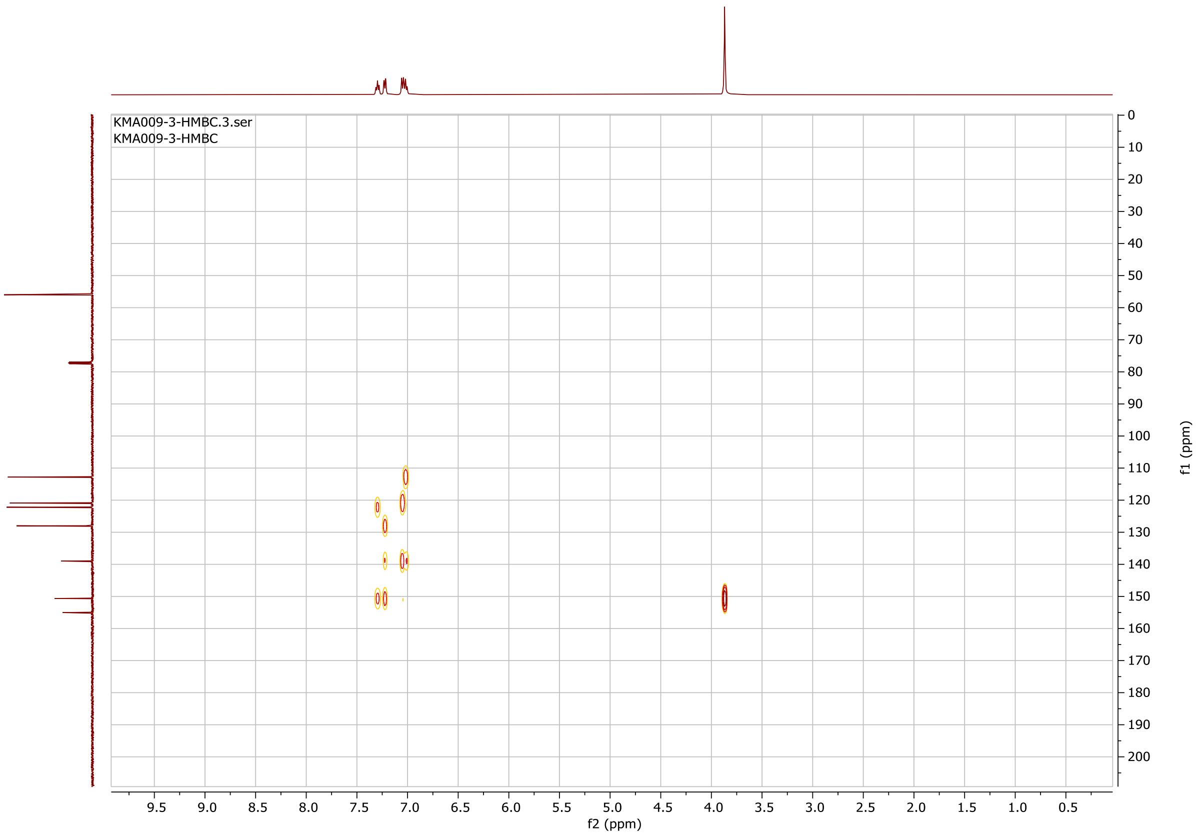

Figure S8. ^{1}H-^{13}C HMBC of DGO in $CDCl_3$

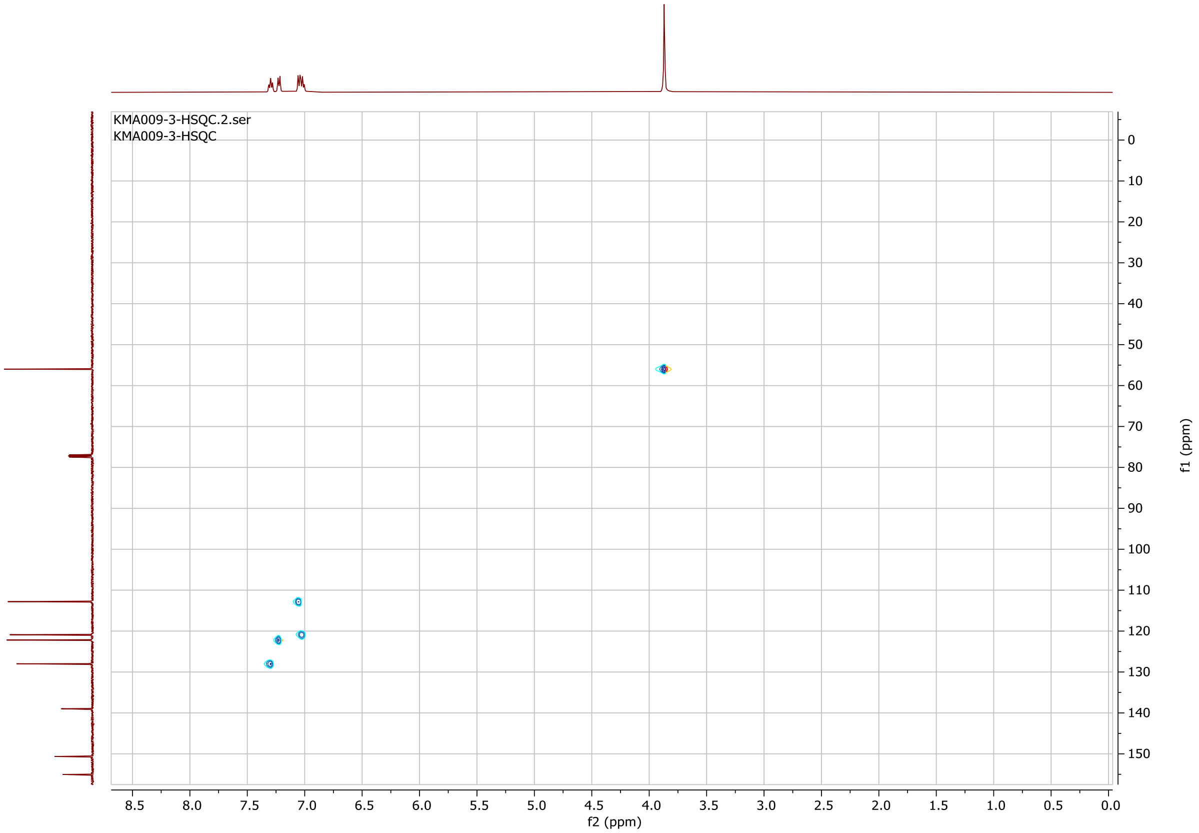

Figure S9. ^{1}H-^{13}C HSQC of DGO in $CDCl_3$

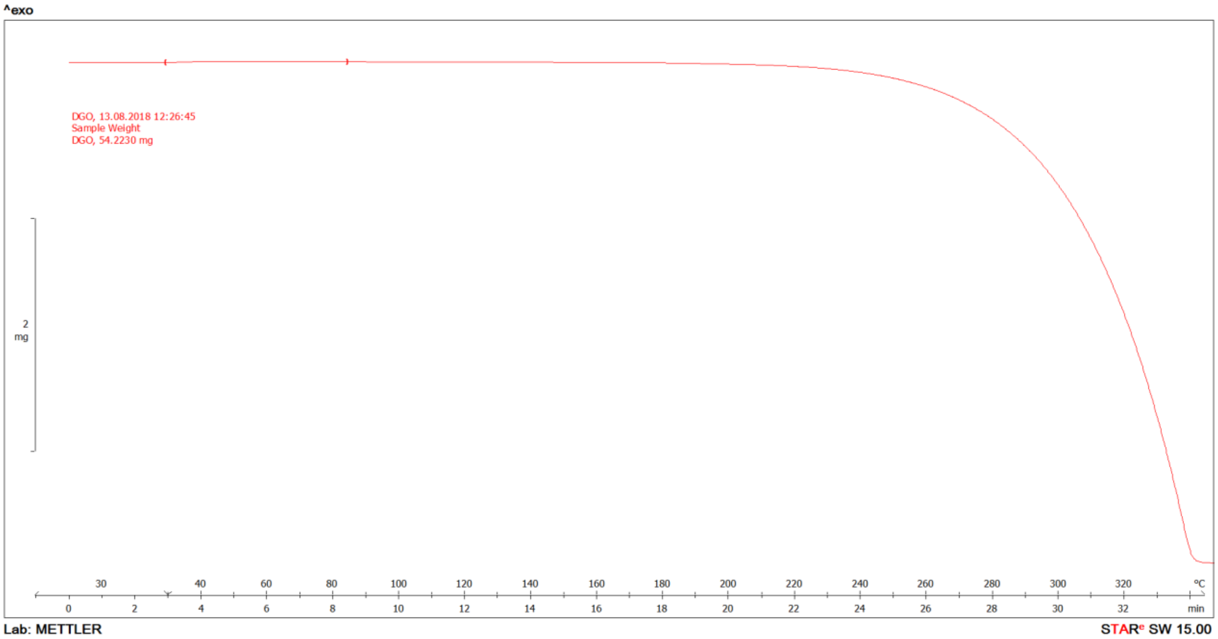

Figure S10. TGA DGO (10°C per minute)

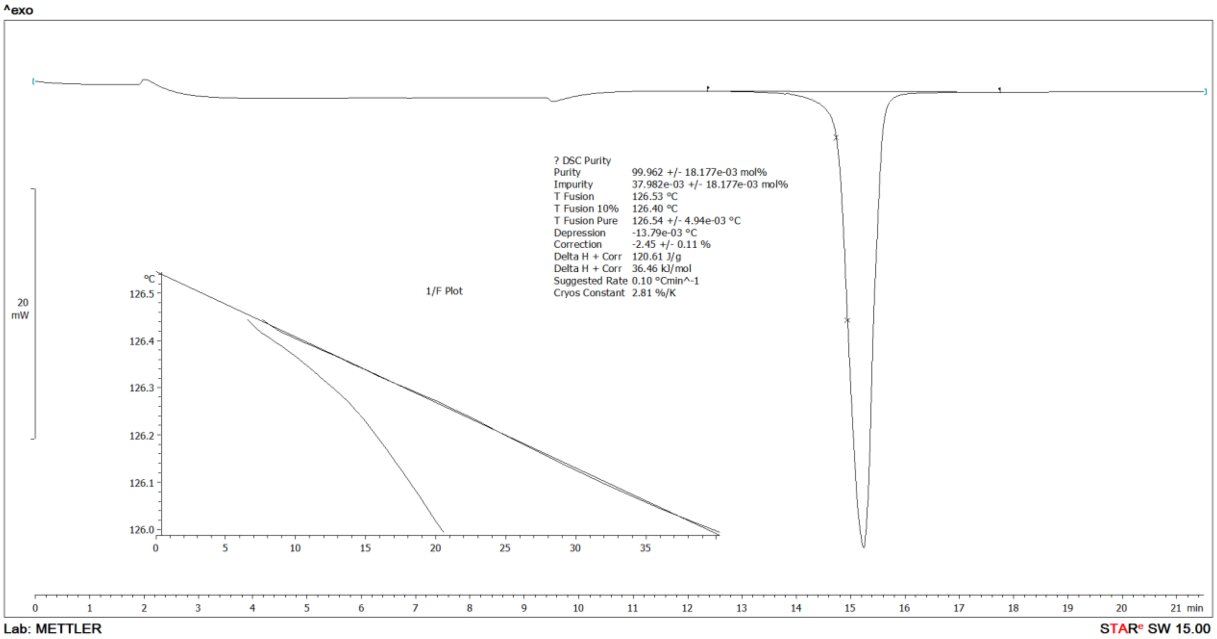

Figure S11. DSC melting curve of DGO from oxalyl chloride with purity analysis. A melting point of 126.53 °C is observed. As the correction factor for the purity analysis is lower than 5% we can accept the value of 99.962 mol%. However this value excludes the impurities which are insoluble in the melt.

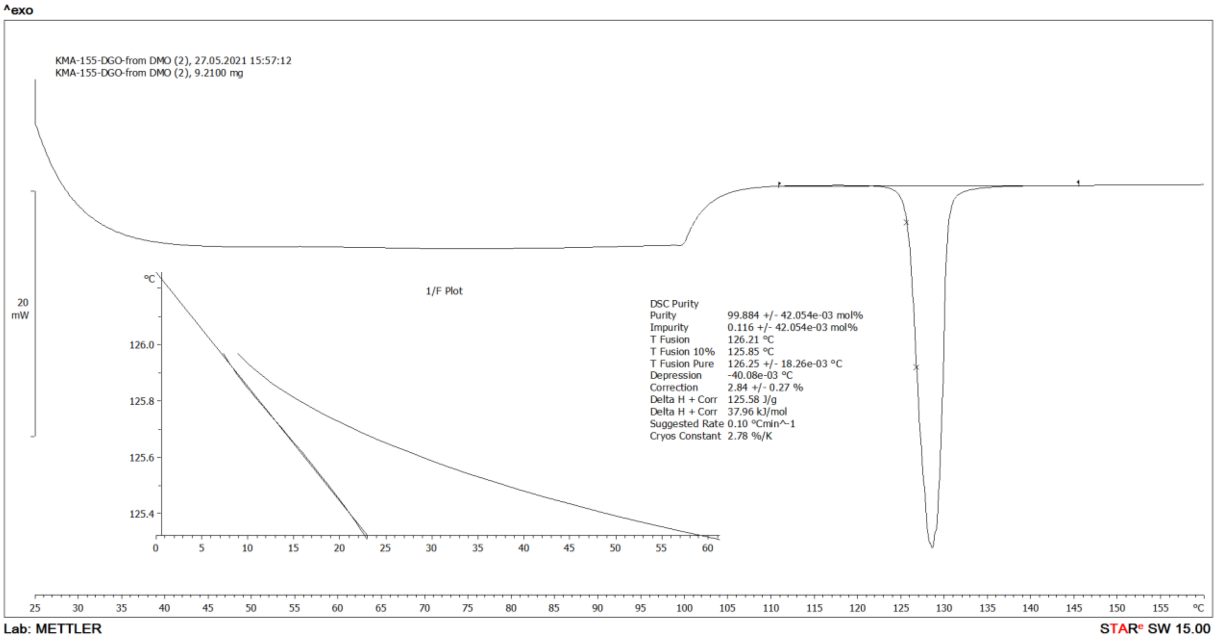

Figure S12. DSC melting curve of DGO from Dimethyl oxalate with purity analysis. A melting point of 126.21°C is observed. As the correction factor for the purity analysis is lower than 5% we can accept the value of 99.884 mol%. However this value excludes the impurities which are insoluble in the melt.

Isosorbide characterization

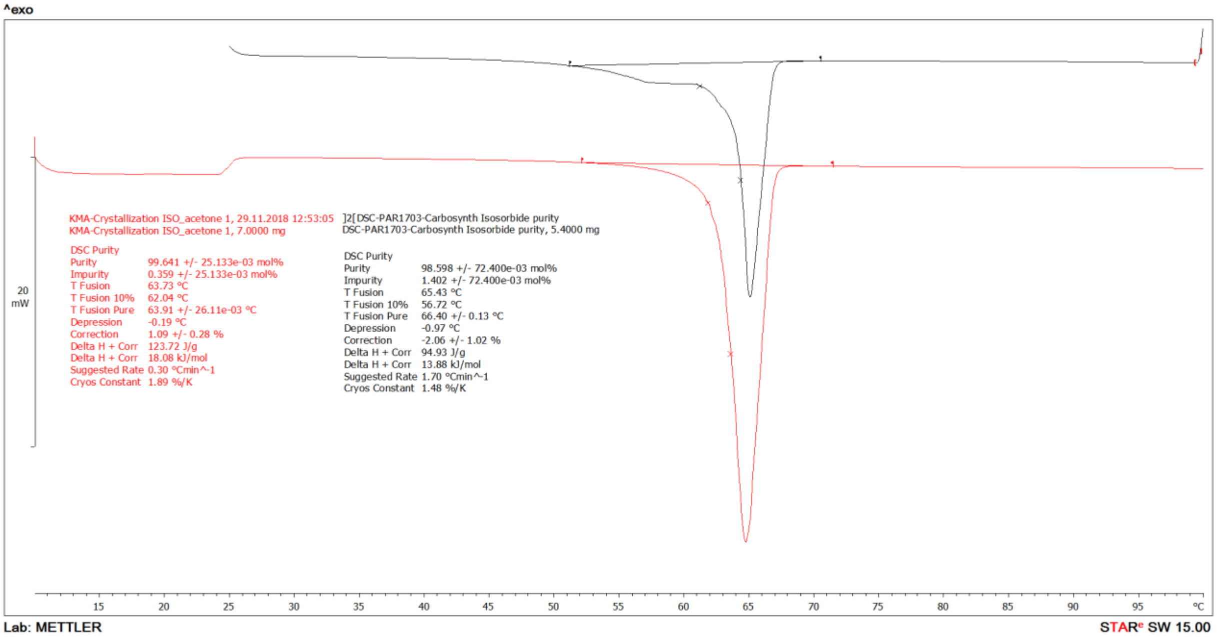

Figure S13. DSC from isosorbide (Carbosynth) and after its recrystallization from acetone.

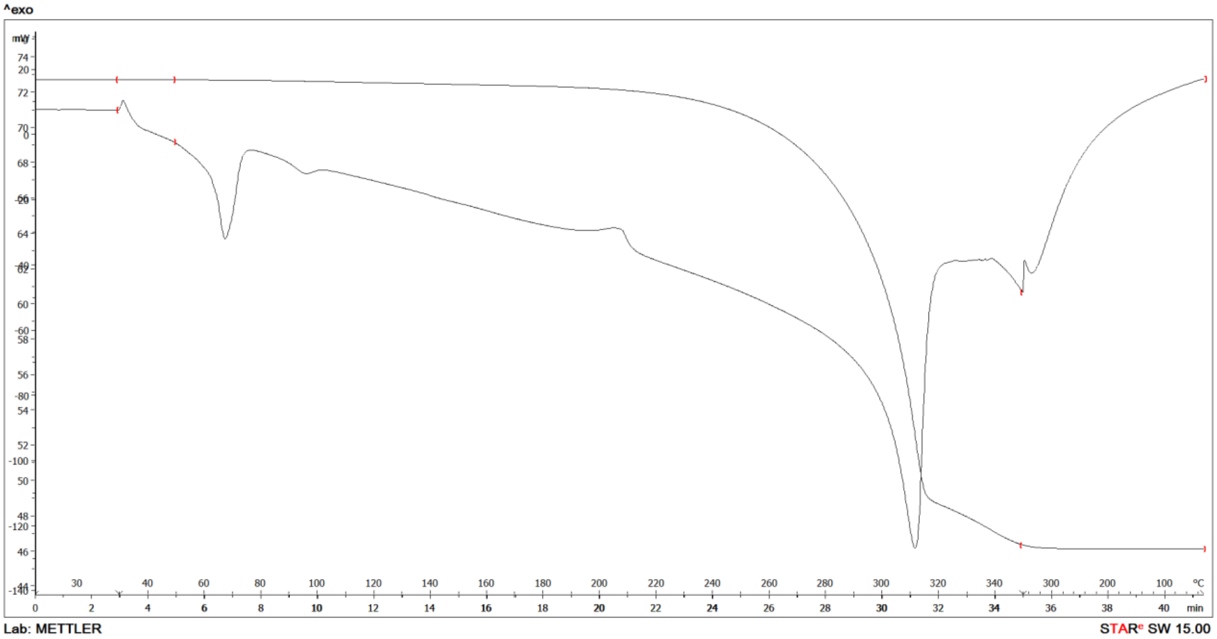

Figure S14. TGA – Isosorbide

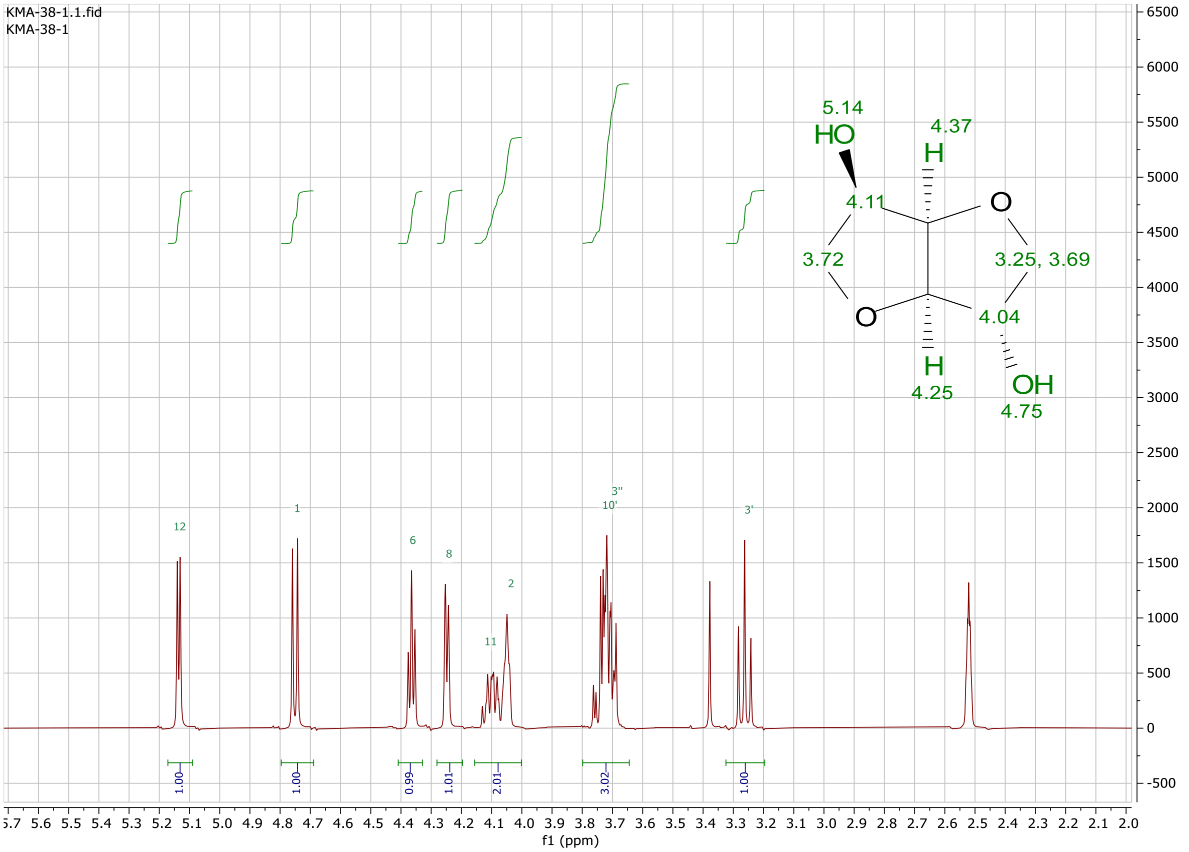

Figure S15. ^{1}H NMR Isosorbide in DMSO after distillation

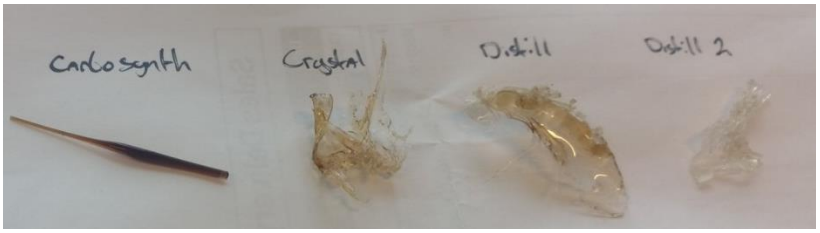

Figure S16. Photos of synthesized PISOX polymers from different processed isosorbide. (Carbosynth) used directly from the supplier, (crystal) recrystallized from acetone, (Distill) distilled, (Distill 2) distilled with sodium borohydride added.

NMR of synthesized PISOX copolymers

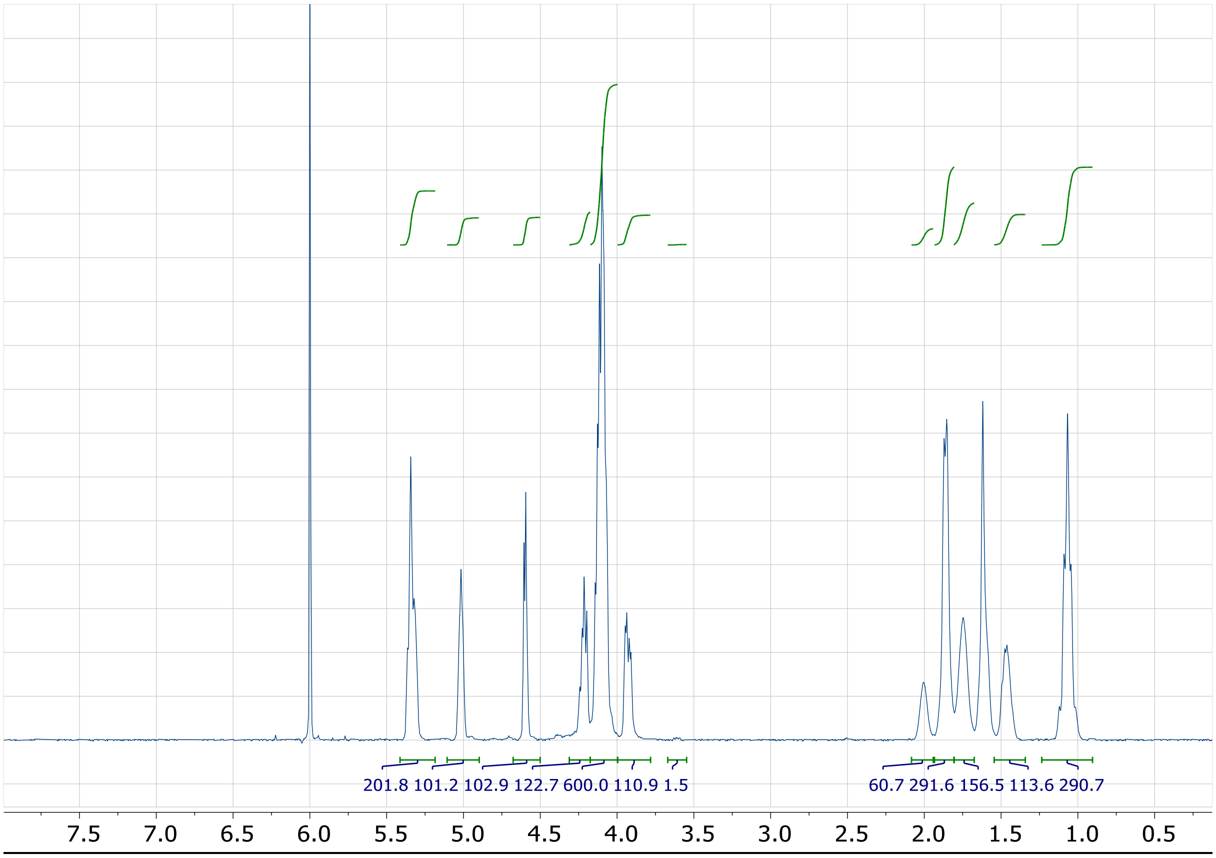

Figure S17. ^{1}H-NMR of PISOX copolymer with 50% cyclohexanedimethanol in TCE-d2.

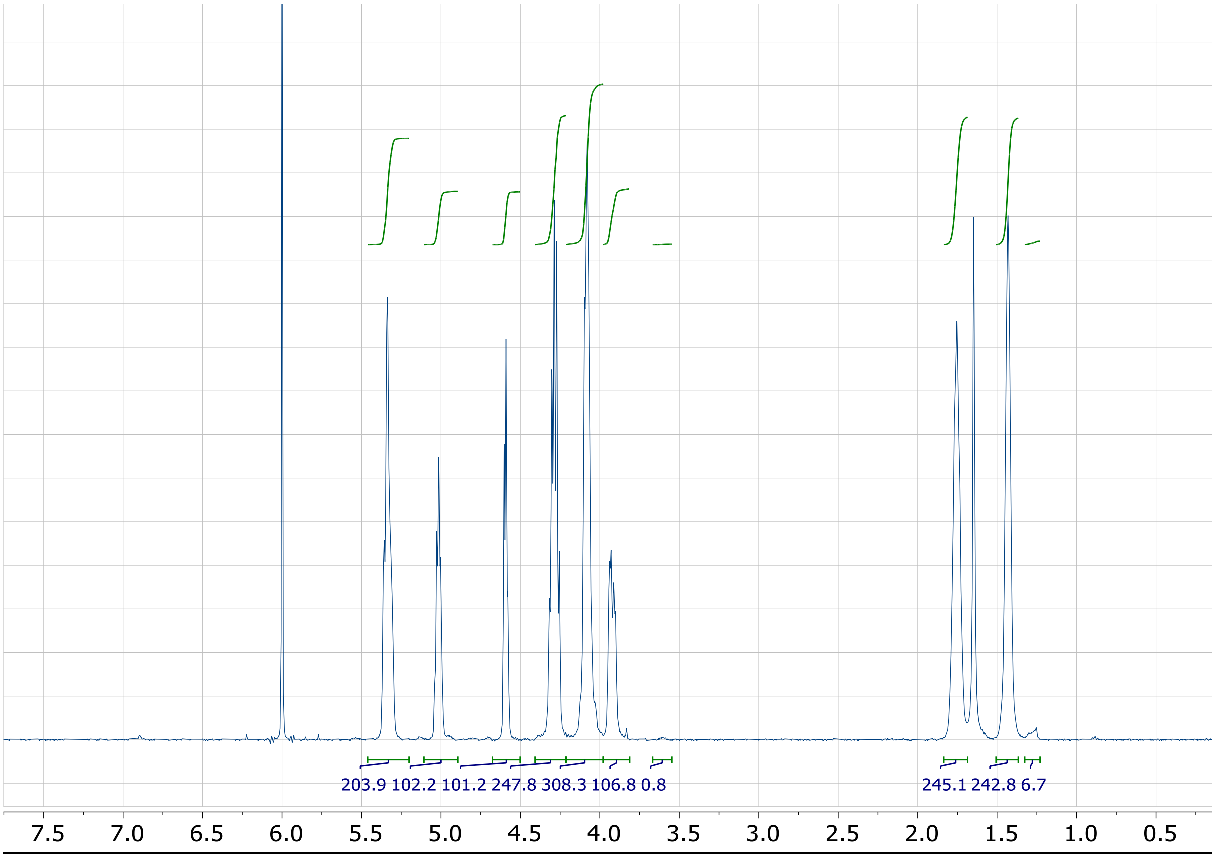

Figure S18. ^{1}H-NMR of PISOX copolymer with 37.5% 1,6-hexanediol in TCE-d2.

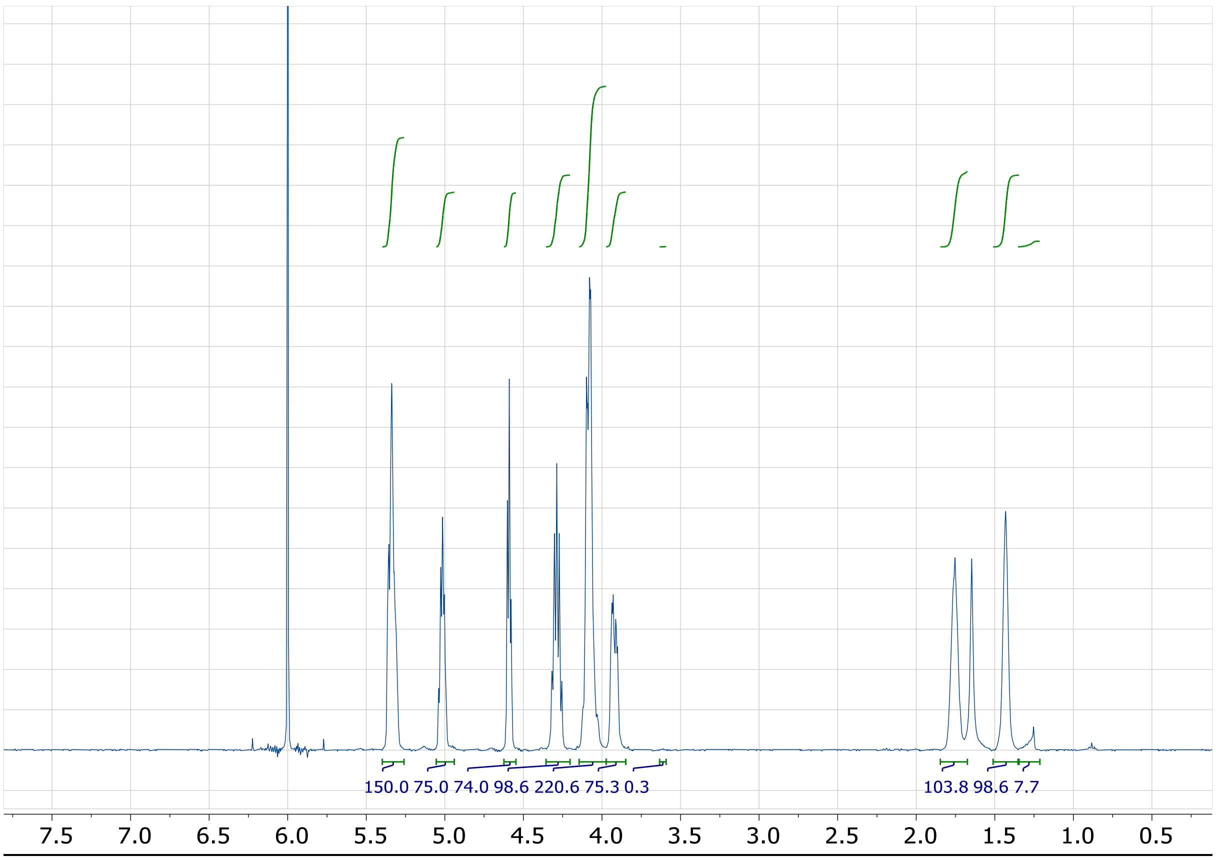

Figure S19. ^{1}H-NMR of PISOX copolymer with 25% 1,6-hexanediol in TCE-d2.

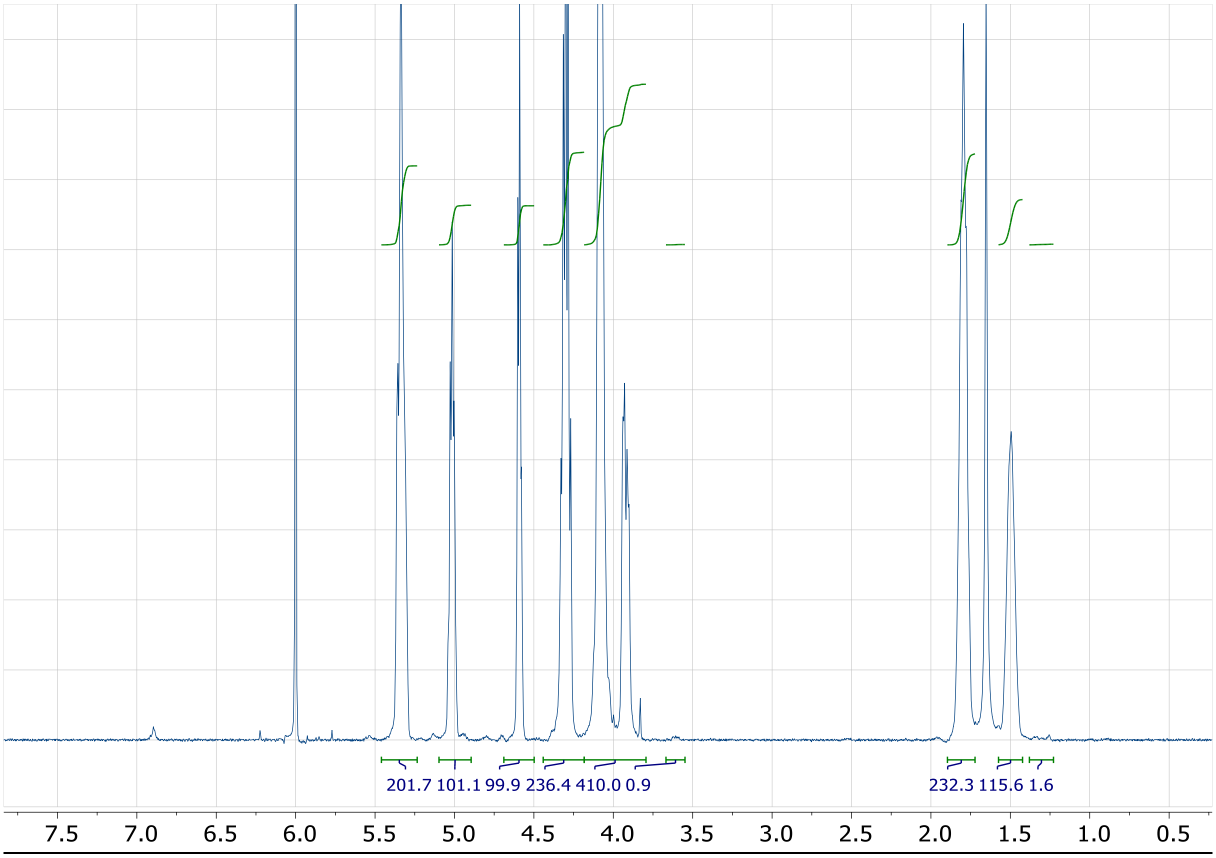

Figure S20. ^{1}H-NMR of PISOX copolymer with 37.5% 1,5-pentanediol in TCE-d2.

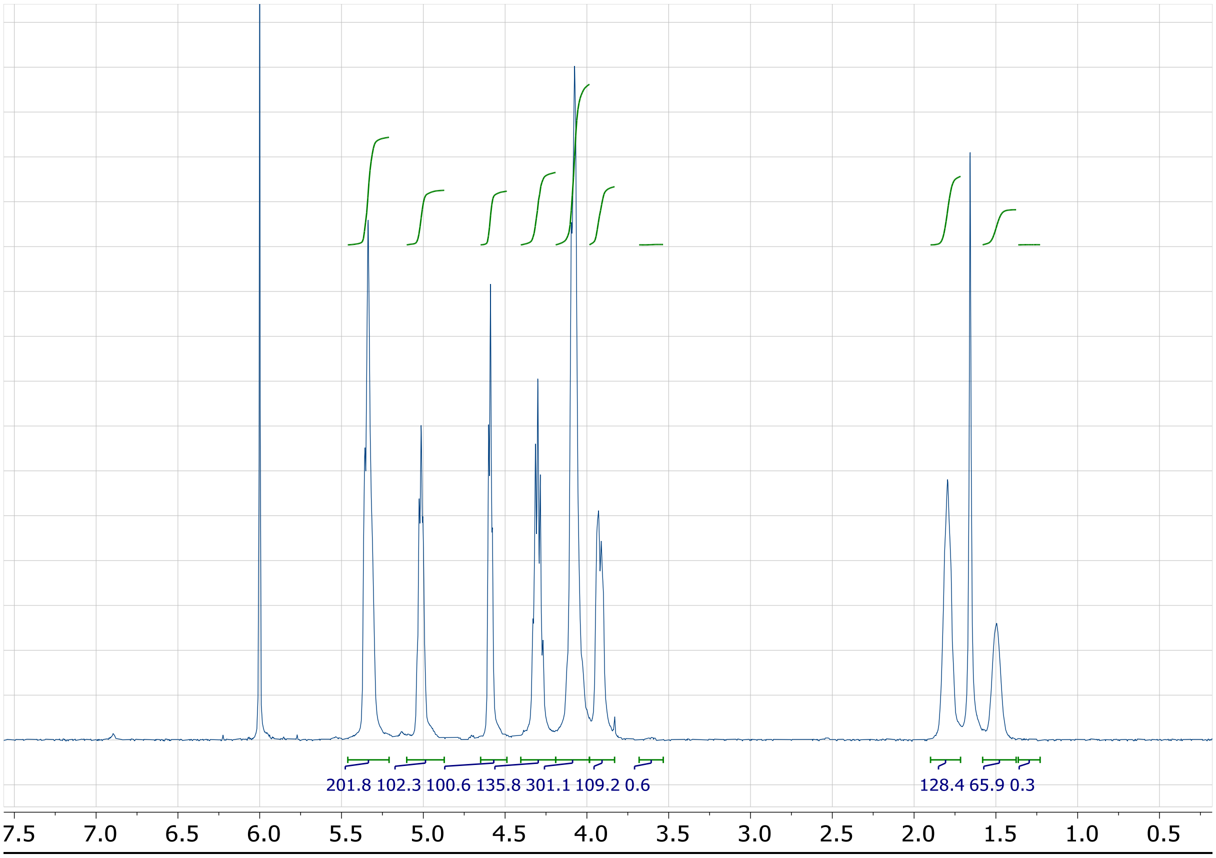

Figure S21. ^{1}H-NMR of PISOX copolymer with 25% 1,5-pentanediol in TCE-d2.

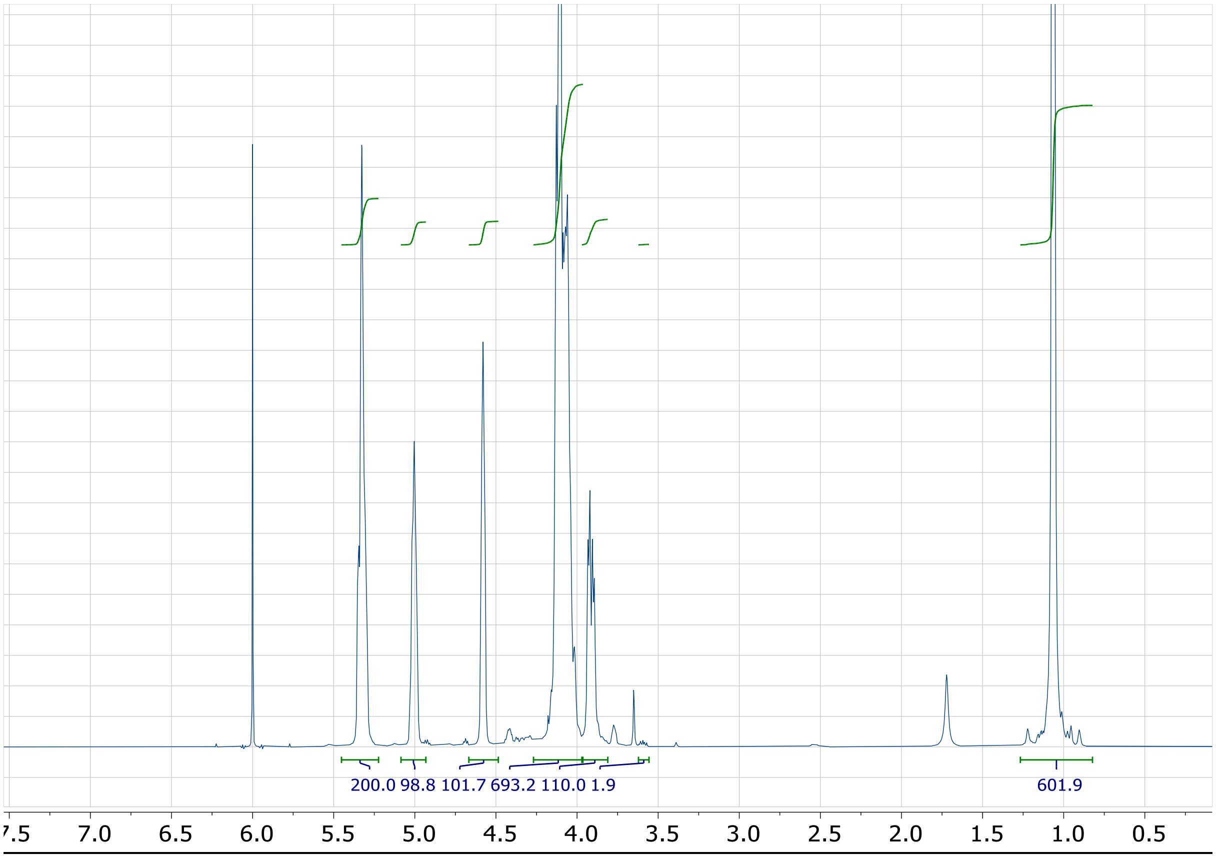

Figure S22. ^{1}H-NMR of PISOX copolymer with 50% Neopentyl glycol in TCE-d2.

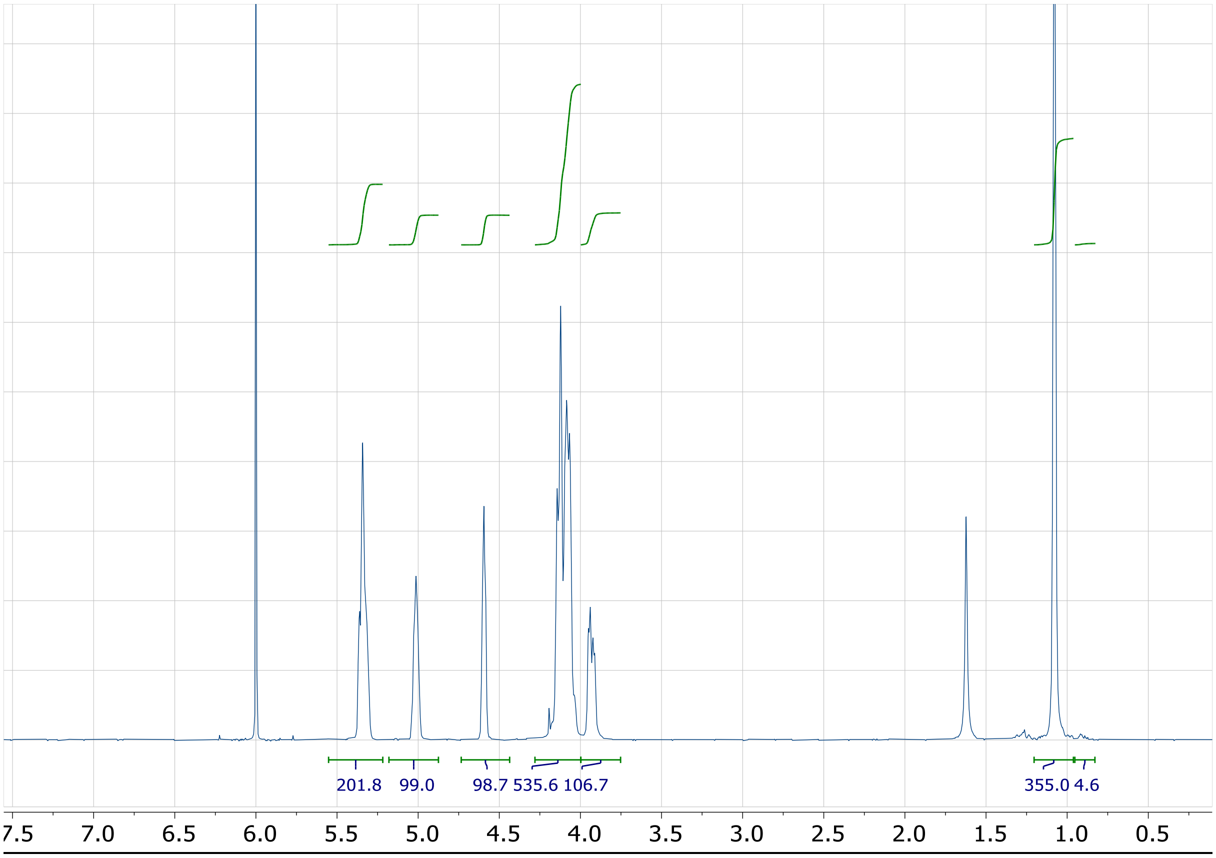

Figure S23. ^{1}H-NMR of PISOX copolymer with 37.5% Neopentyl glycol in TCE-d2.

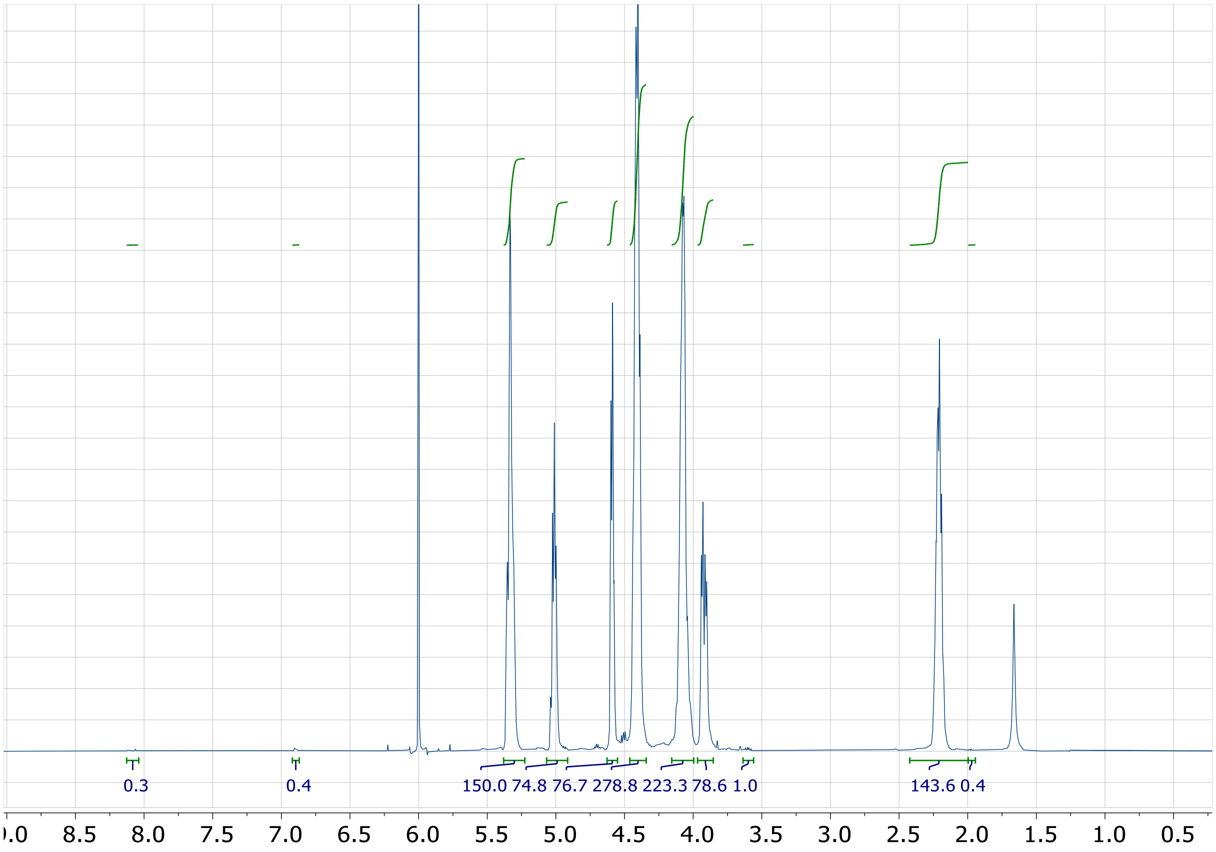

Figure S24. ^{1}H-NMR of PISOX copolymer with 50%1,3-propanediol in TCE-d2.

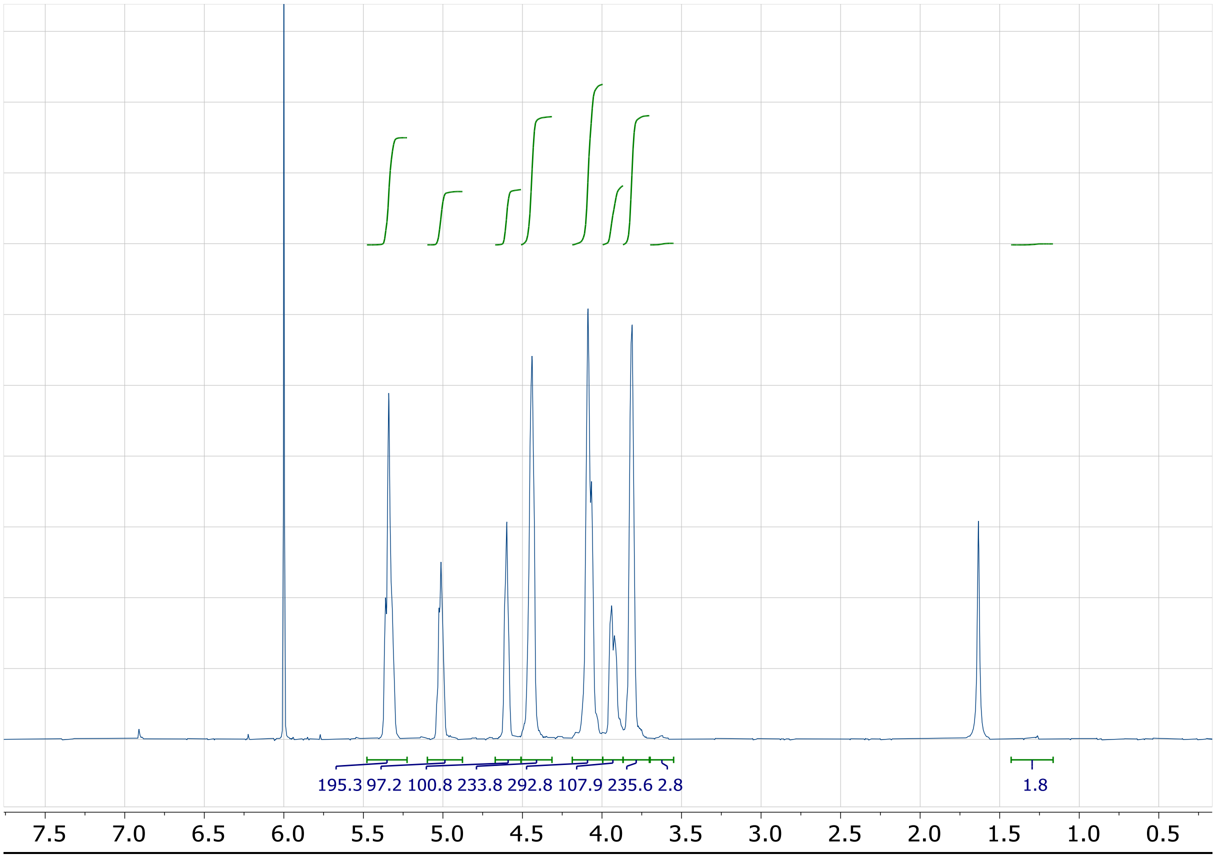

Figure S25. ^{1}H-NMR of PISOX copolymer with 37.5% Diethylene Glycol in TCE-d2.

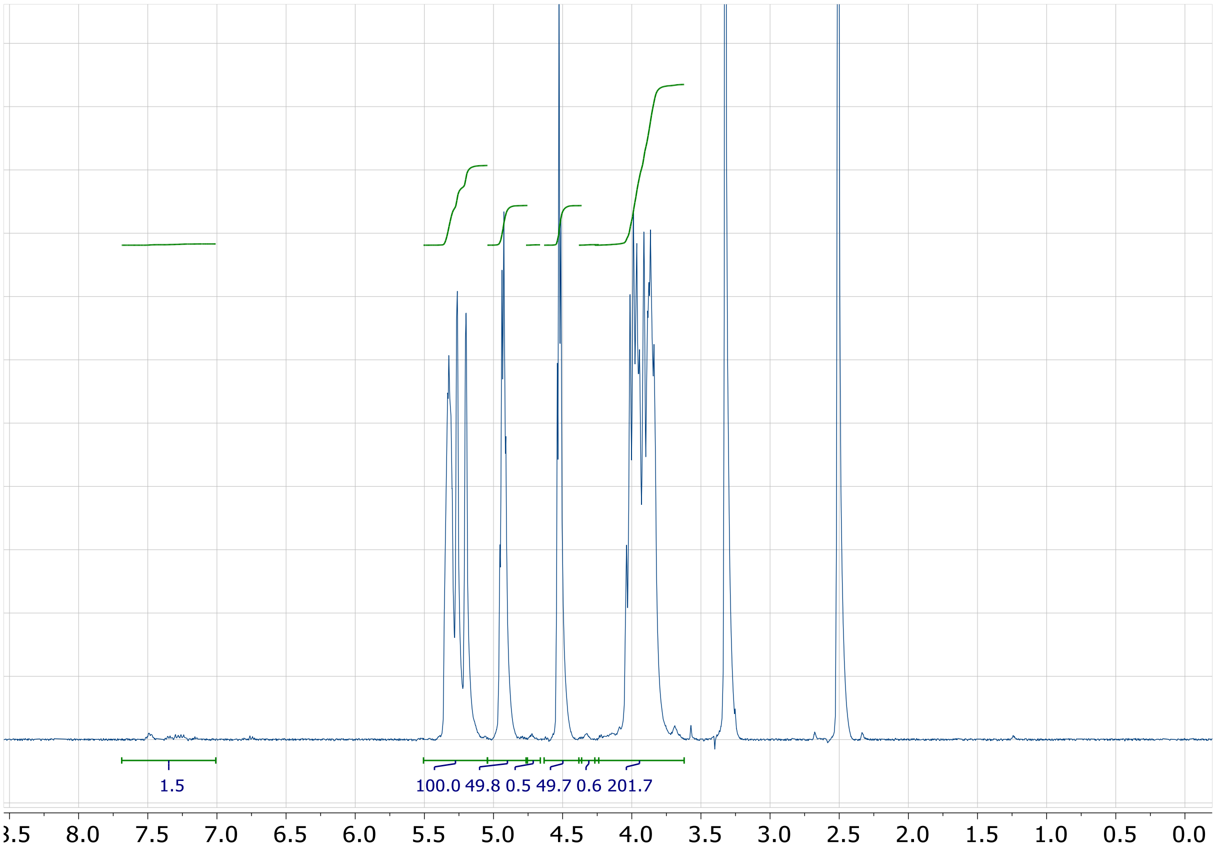

Figure S26. ^{1}H-NMR of PISOX 100% Isosorbide in DMSO-d6.

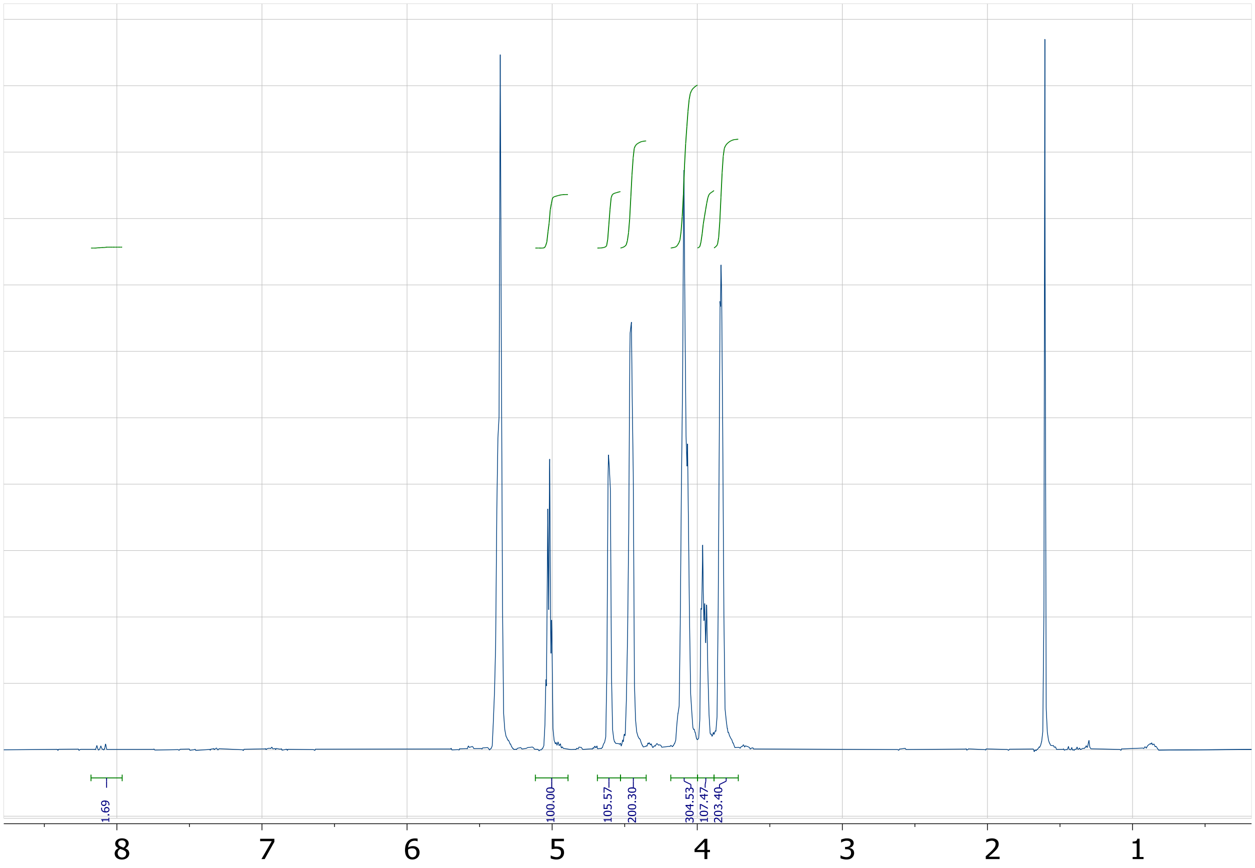

Figure S27. ¹H-NMR of PISOX DEG 32.5% autoclave experiment in DCM-d2.

Thermal Data of PISOX copolymers

Table S3. Overview of the data used for determining the slope of co-diol content.

Type of diol	% Diol	Tg (°C)	Slope	Intersect	R^2
HDO	37.6	76	-2.41	166.8	1.0000
	24.7	107			
PDO	36.4	85	-2.25	166.3	0.9995
	24.5	110			
BDO*	17.4	131	-2.11	166.2	0.9973
	47.3	68			
	27.6	105			
NPG	50.0	83	-1.70	166.3	0.9990
	37.0	102			
DEG*	26.2	108	-2.12	166.0	0.9973
	37.5	88			
Only ISO	0	167	-	-	-

*obtained from literature.[19]

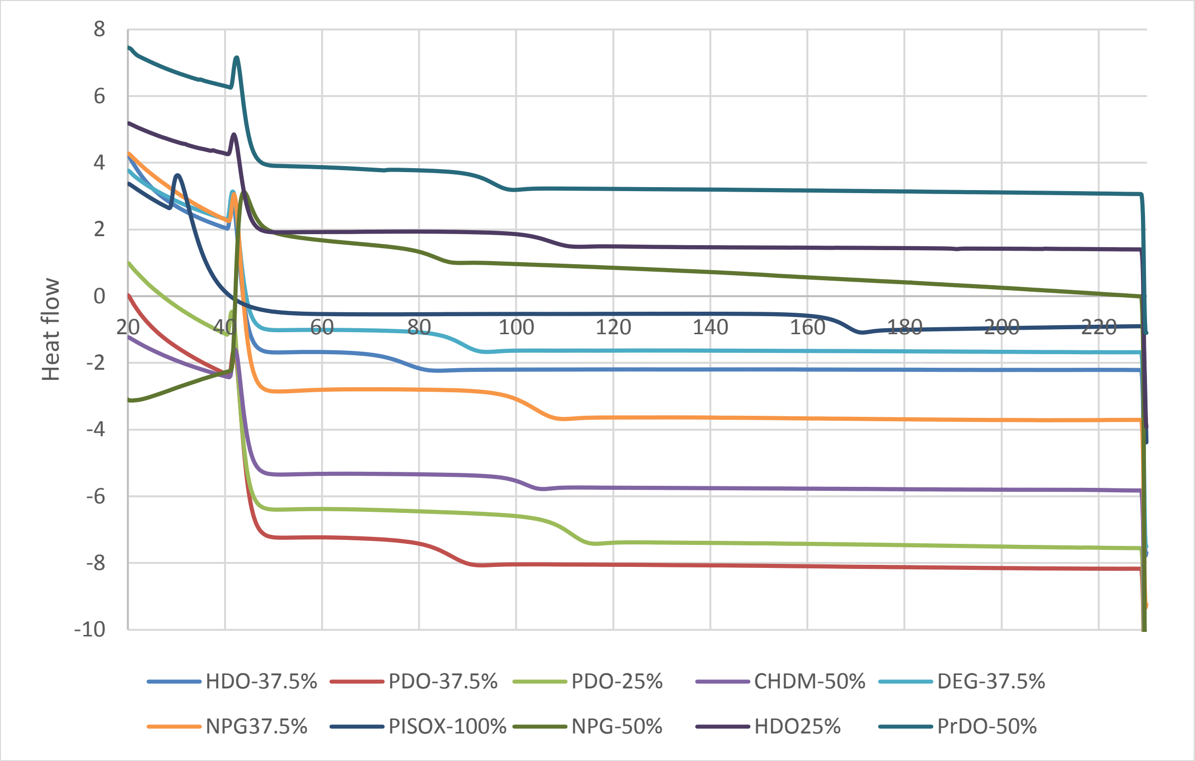

Figure S28. Stacked DSC raw data of the PISOX copolymers.

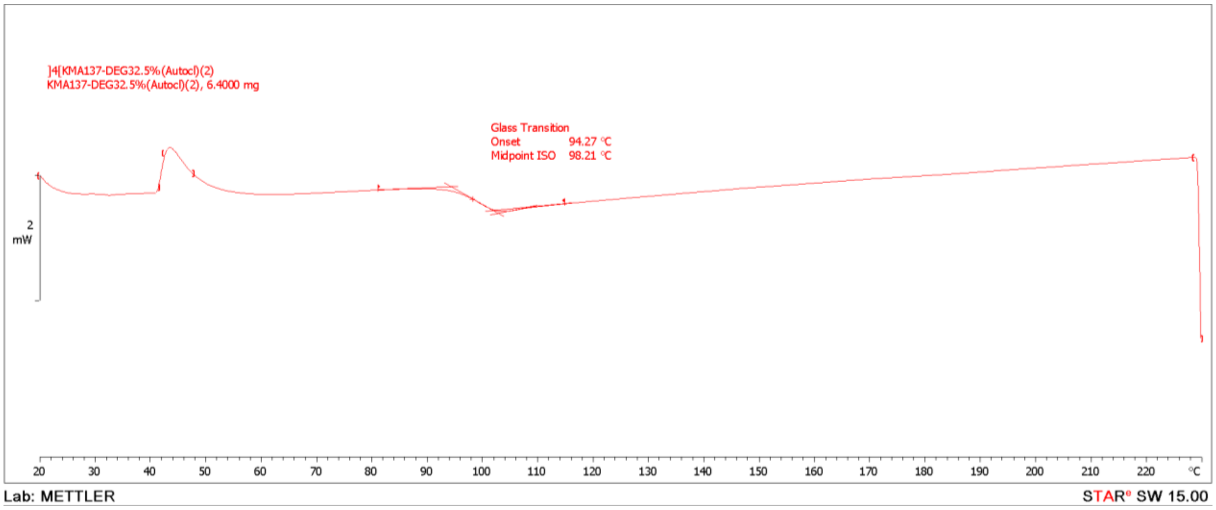

Figure S29 DSC autoclave experiment PISOX DEG

Table S4. Overview of the reaction stage between isosorbide and the diaryl esters. Q_r = reaction quotient and $\bar{X}_n$ = Average degree of polymerization.

DGO			
Time (h)	Guaiacol free (%)	Q_r	$\bar{X}_n$
0	7.1	0.01	1.1
1	91.2	106	11.3
2	93.6	211	15.5
3	93.4	202	15.2

DPO			
Time (h)	Phenol free (%)	Q_r	$\bar{X}_n$
0	1.6	0	1.0
1	54.1	1	2.2
2	79.8	16	4.9
3	87.1	46	7.8

DPC			
Time (h)	Phenol free (%)	Q_r	$\bar{X}_n$
1	38.0	0.38	1.61
3	45.3	0.69	1.83
6	51.7	1.14	2.07

DGC			
Time (h)	Guaiacol free (%)	Q_r	$\bar{X}_n$
1	4.1	0.00	1.04
3	16.9	0.04	1.20
6	32.6	0.23	1.48

DPT			
Time (h)	Phenol free (%)	Q_r	$\bar{X}_n$
1	1.7	0.00	1.02
3	3.0	0.00	1.03
6	9.5	0.01	1.10

DGT			
Time (h)	Guaiacol free (%)	Q_r	$\bar{X}_n$
1	0.2	0.00	1.00
3	1.2	0.00	1.01
6	4.5	0.00	1.05

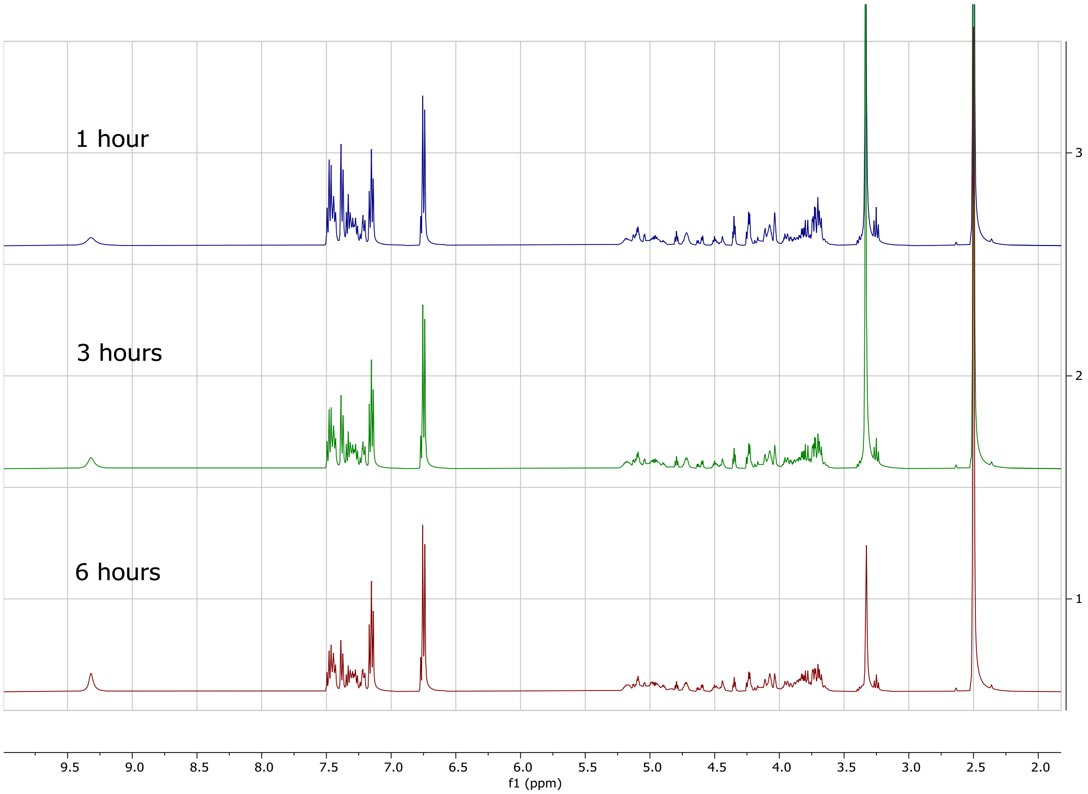

Figure S30. Stacked ^{1}H NMR spectra (DMSO) of the reaction (190 °C oil temperature) between Diphenyl carbonate (**DPC**) and Isosorbide (1:1).

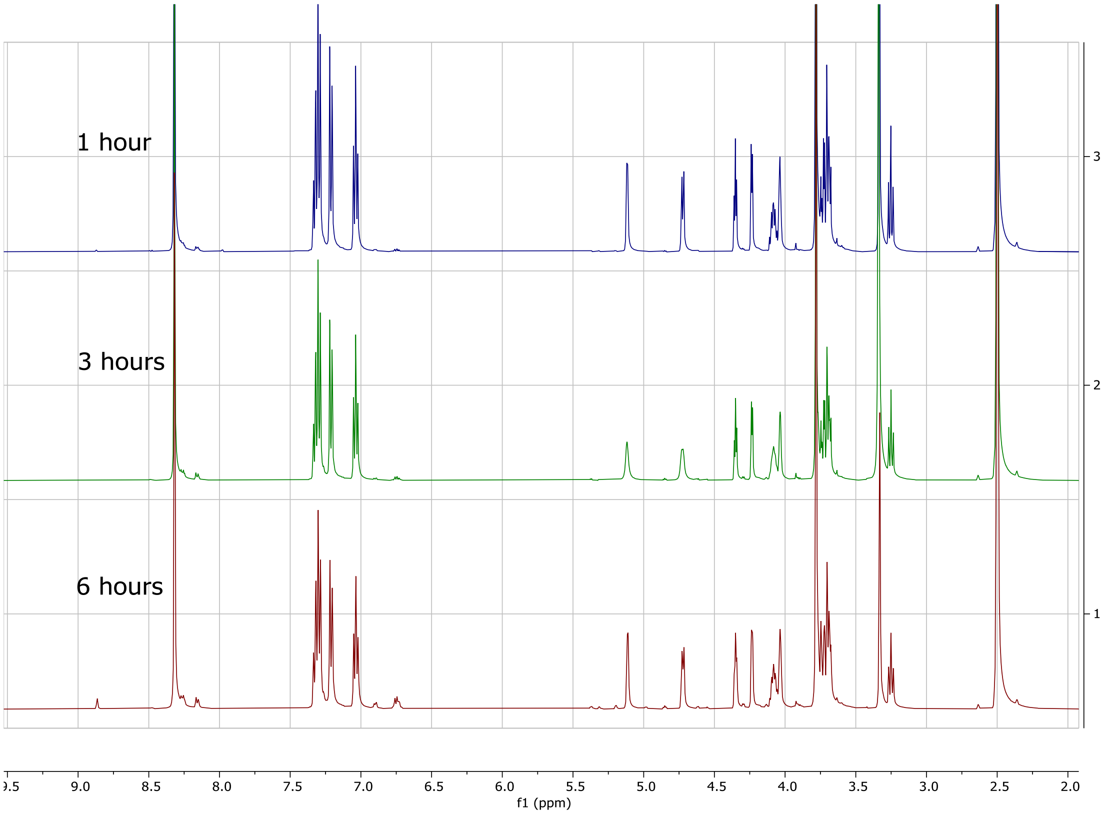

Figure S31. Stacked ^{1}H NMR spectra (DMSO) of the reaction (190°C oil temperature) between Diguaiacyl terephthalate (**DGT**) and Isosorbide (1:1).

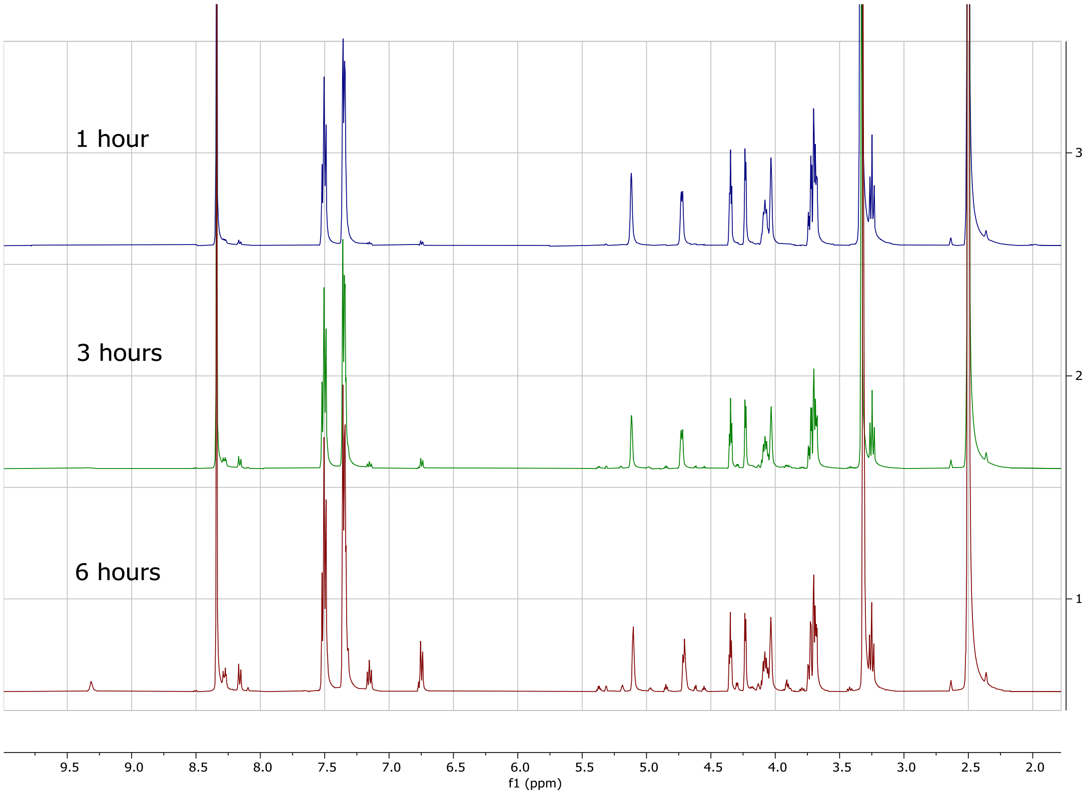

Figure S32. Stacked ^{1}H NMR spectra (DMSO) of the reaction (190°C oil temperature) between Diphenyl terephthalate (**DPT**) and Isosorbide (1:1).

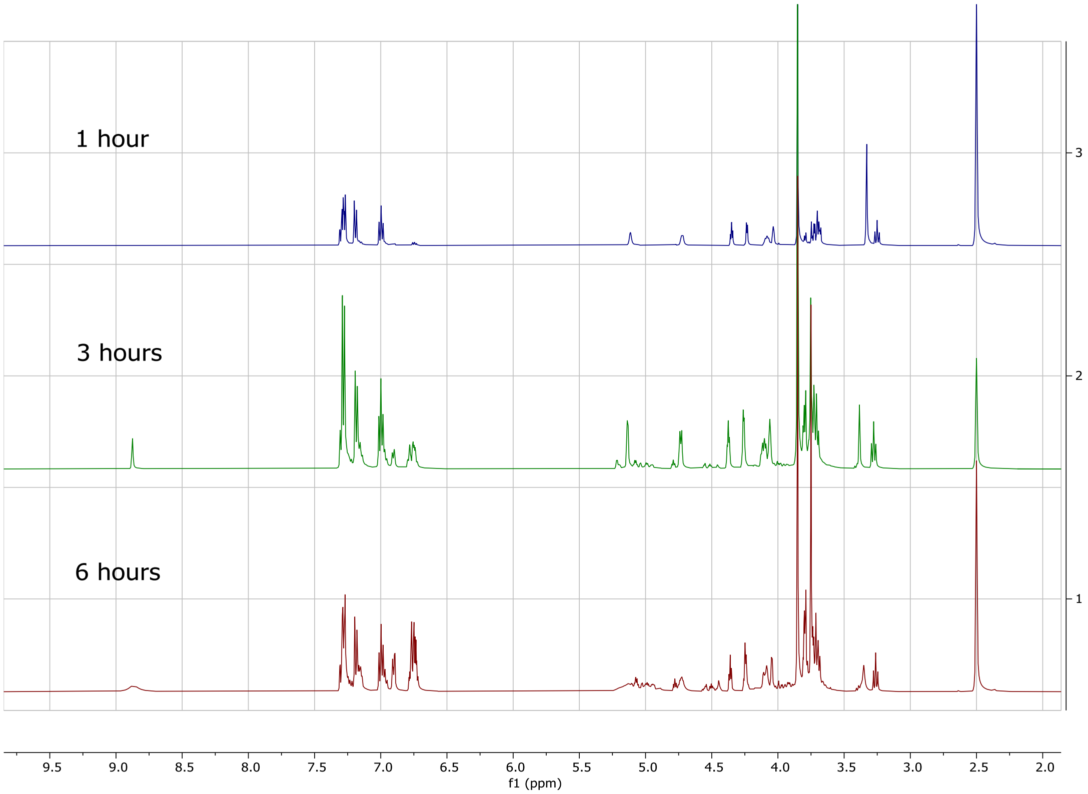

Figure S33. Stacked ^{1}H NMR spectra (DMSO) of the reaction (190°C oil temperature) between Diguaiacyl carbonate (**DGC**) and Isosorbide (1:1).

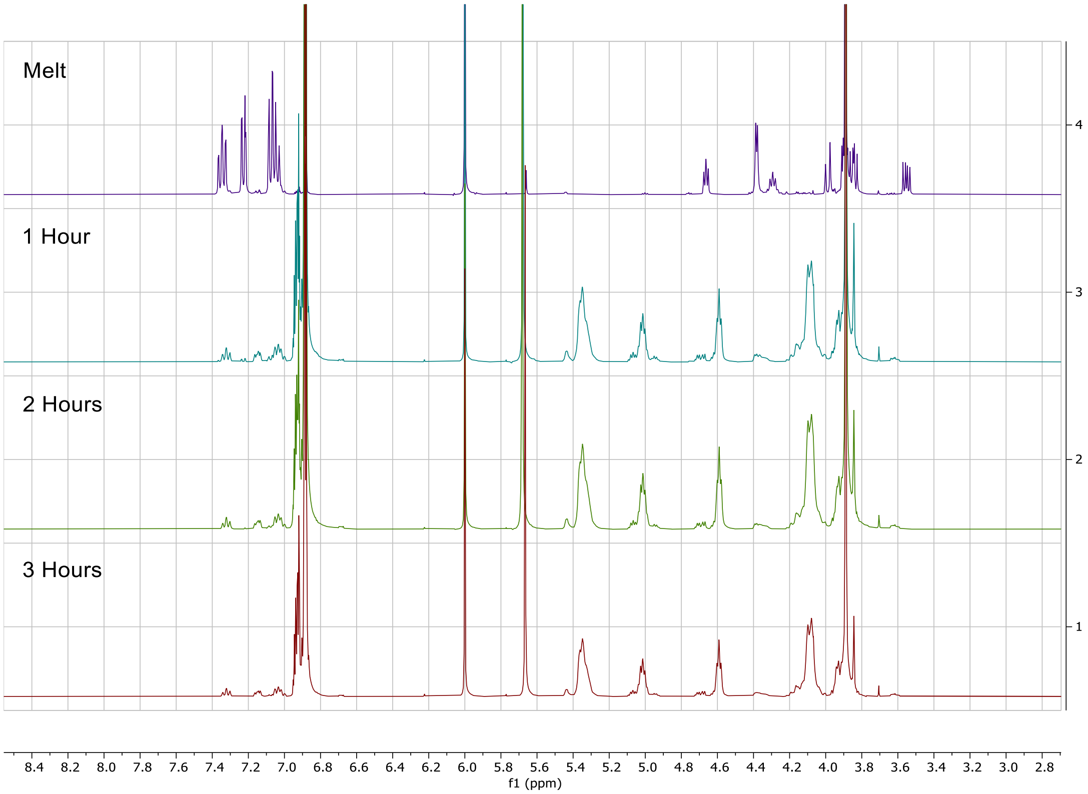

Figure S34. Stacked ^{1}H NMR spectra (TCE-d2) of the reaction (190°C oil temperature) between Diguaiacyl oxalate (**DGO**) and Isosorbide (1:1).

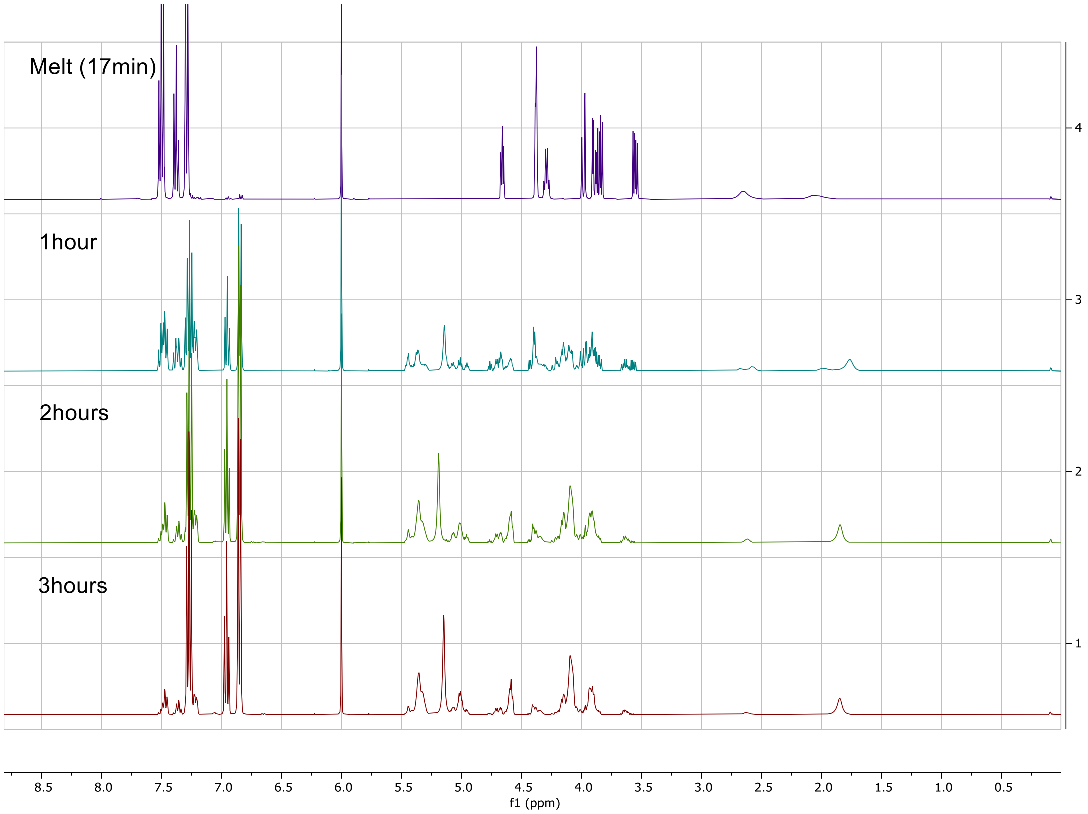

Figure S35. Stacked ^{1}H NMR spectra (TCE-d2) of the reaction (190°C oil temperature) between Diphenyl oxalate (**DPO**) and Isosorbide (1:1).

Barrier data

Table S5. Overview of all barrier data from the PISOX films.

Experiment	Type of Diol	Molar content	OTR	OTR	WVTR	Film thickness
KMA71	CHDM (film	50	59.1	50.8	36.47	120
Daniel	CHDM (film2)	50	68	56	38	100
KMA67A	HDO	37.6	46.9	35.8	42.5	100
SSA 013	HDO (film 1)	24.7	41.8	36.7	47.3	120
KMA58	HDO (film 2)	25.1	48.0	32.0	53.0	100
KMA29	HDO (film 3)	24.6	47.0	31.0	54.0	100
KMA67B	PDO (film 1)	36.4	35.1	24.7	41.4	100
PISOX1902	PDO (film2)	36.7	30.0	22.0	37.0	100
KMA69	PDO	24.5	34.7	24.6	48.3	100
SSA 004	NPG	50	43.0	34.0	36.7	100
KMA75B	NPG	37.0	43.1	32.7	44.1	110
SSA 003	PrDO	49	14.5	11.2	33.0	100
KMA74B	DEG	37.5	10.7	6.1	30.3	100
	RamaPET	-	55.5	47.2	15.17	100

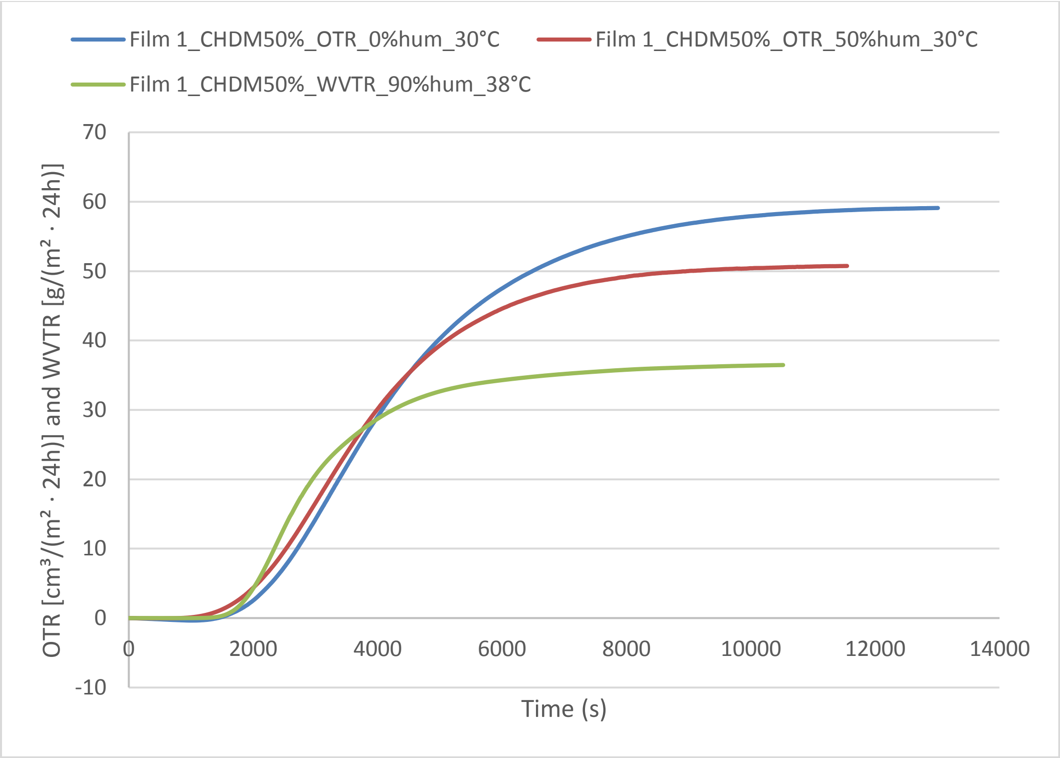

Figure S36. Permeability measurements PISOX 50% CHDM (Film 1)

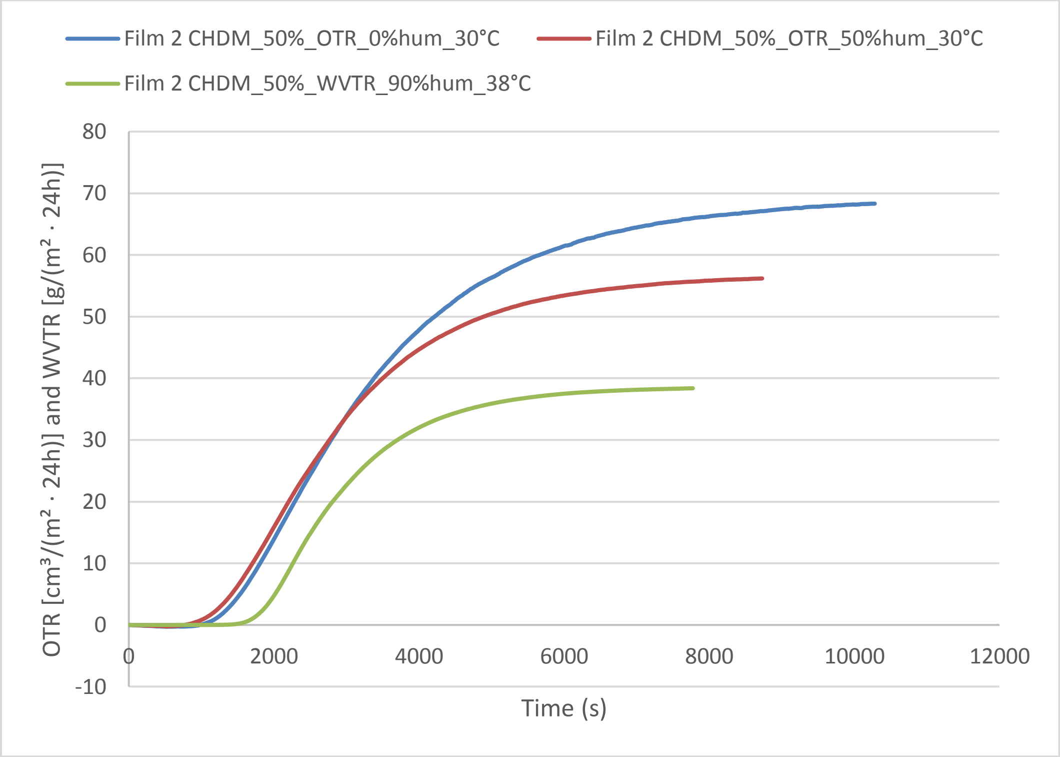

Figure S37. Permeability measurements PISOX CHDM 50% (Film 2)

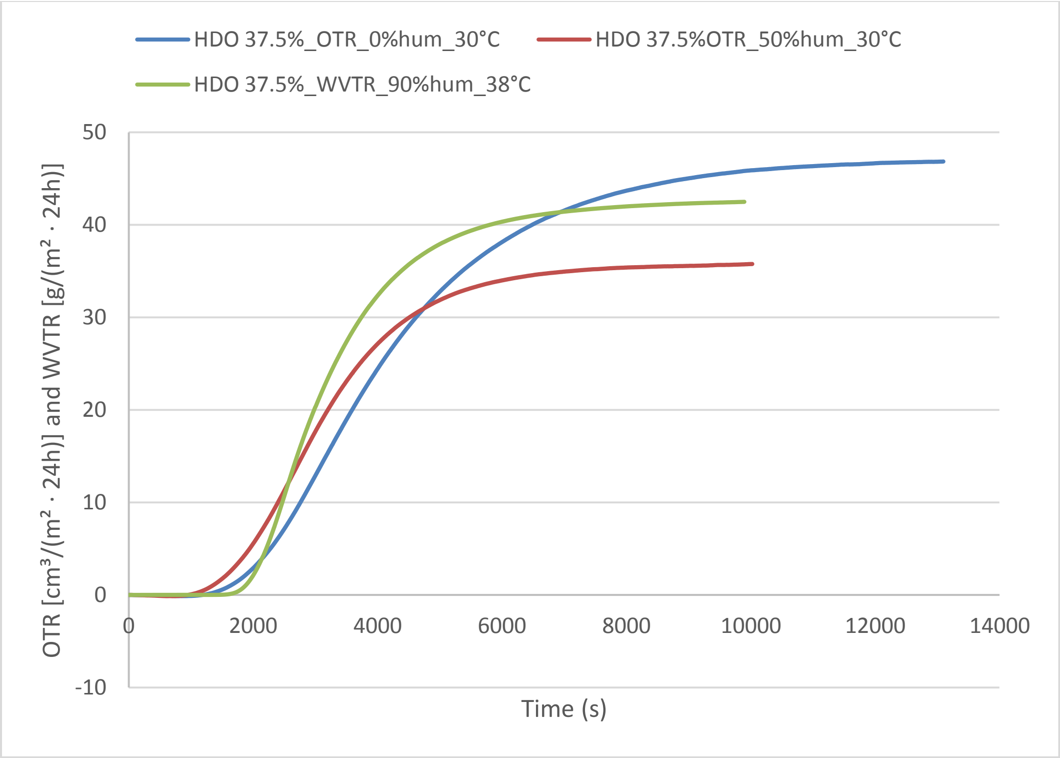

Figure S38. Permeability measurements PISOX HDO 37.5%

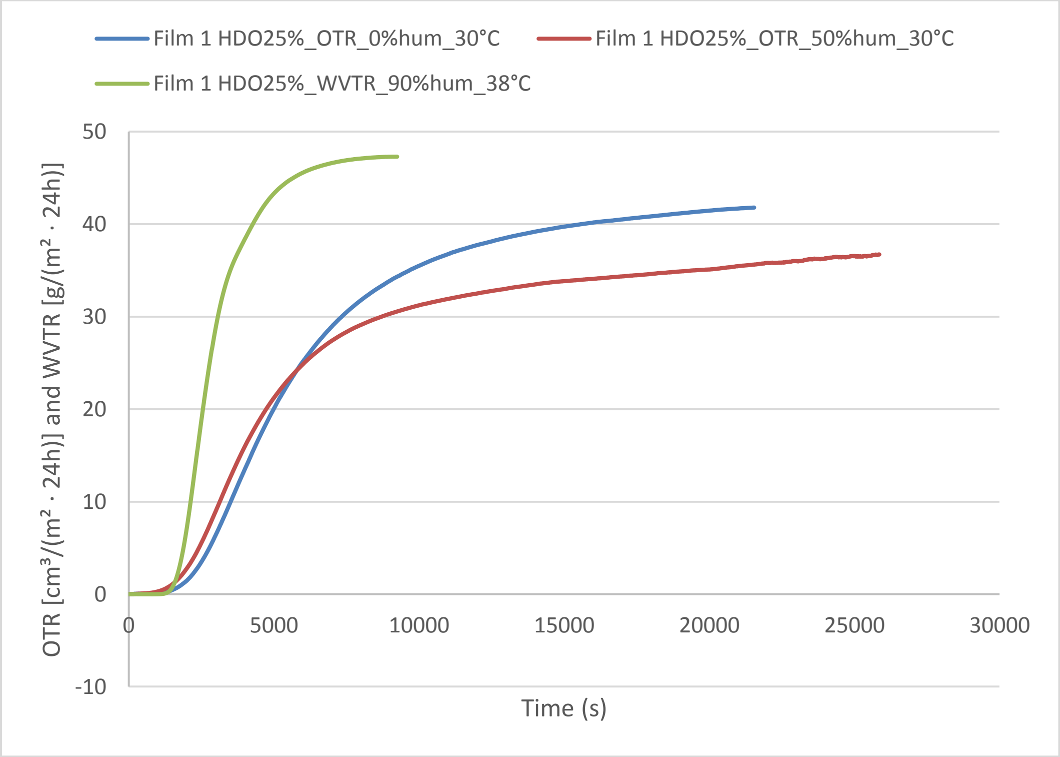

Figure S39. Permeability measurements PISOX HDO 25% (Film 1)

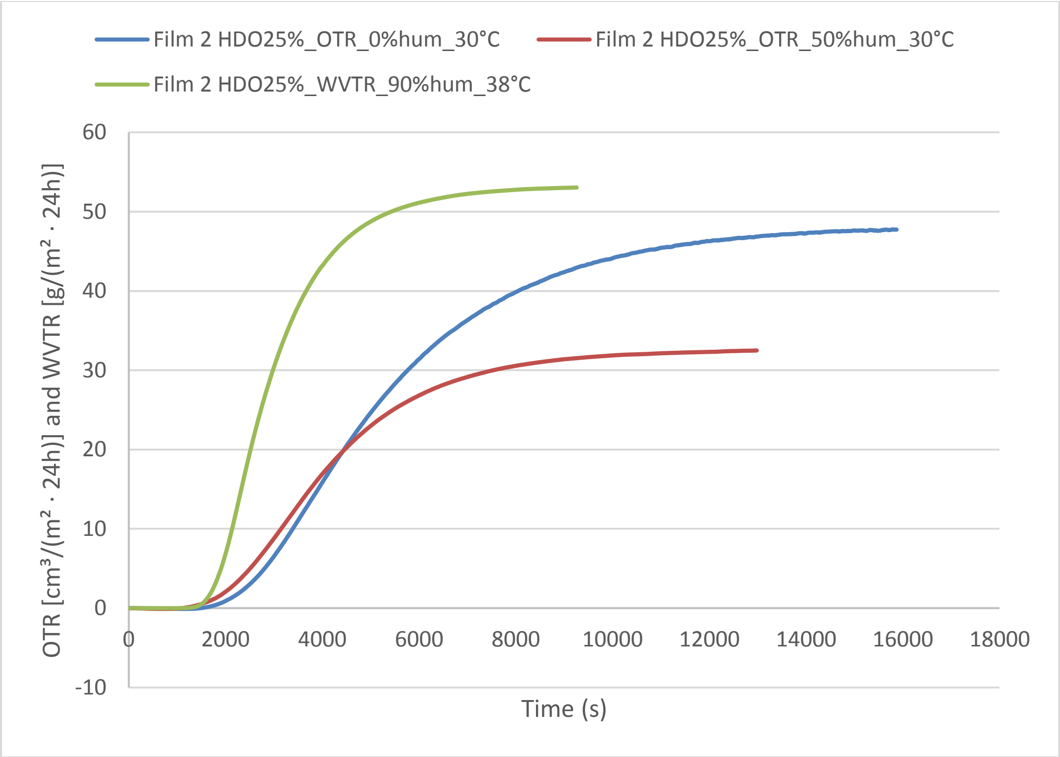

Figure S40. Permeability measurements PISOX HDO 25% (film 2)

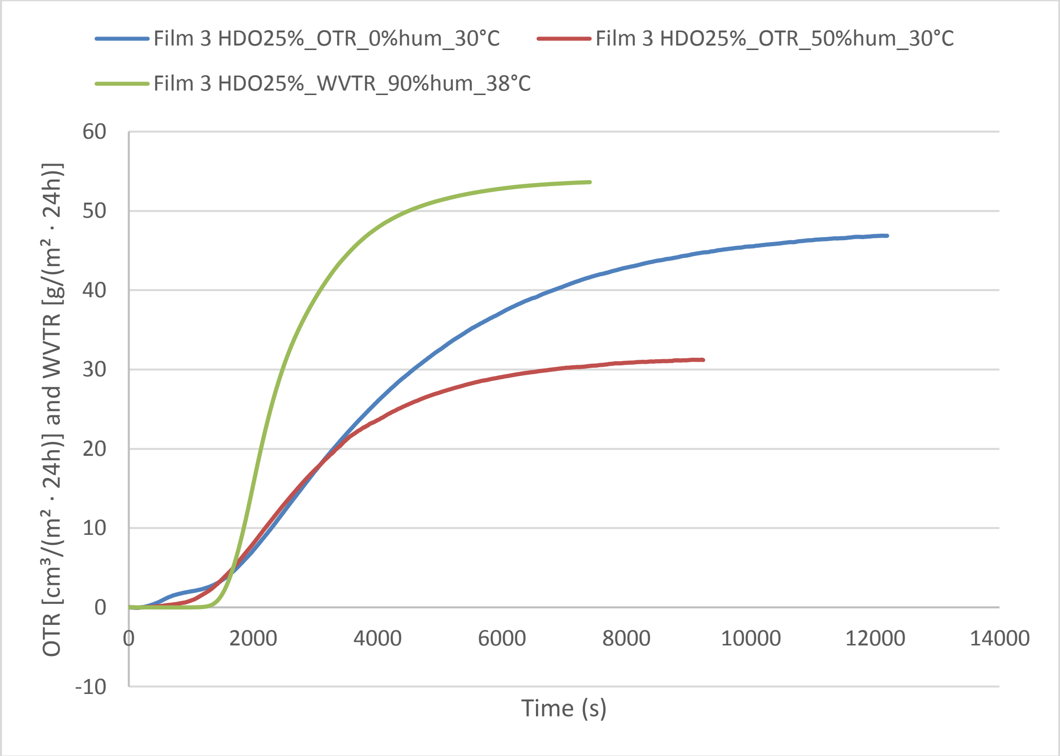

Figure S41. Permeability measurements PISOX HDO 25% (film 3)

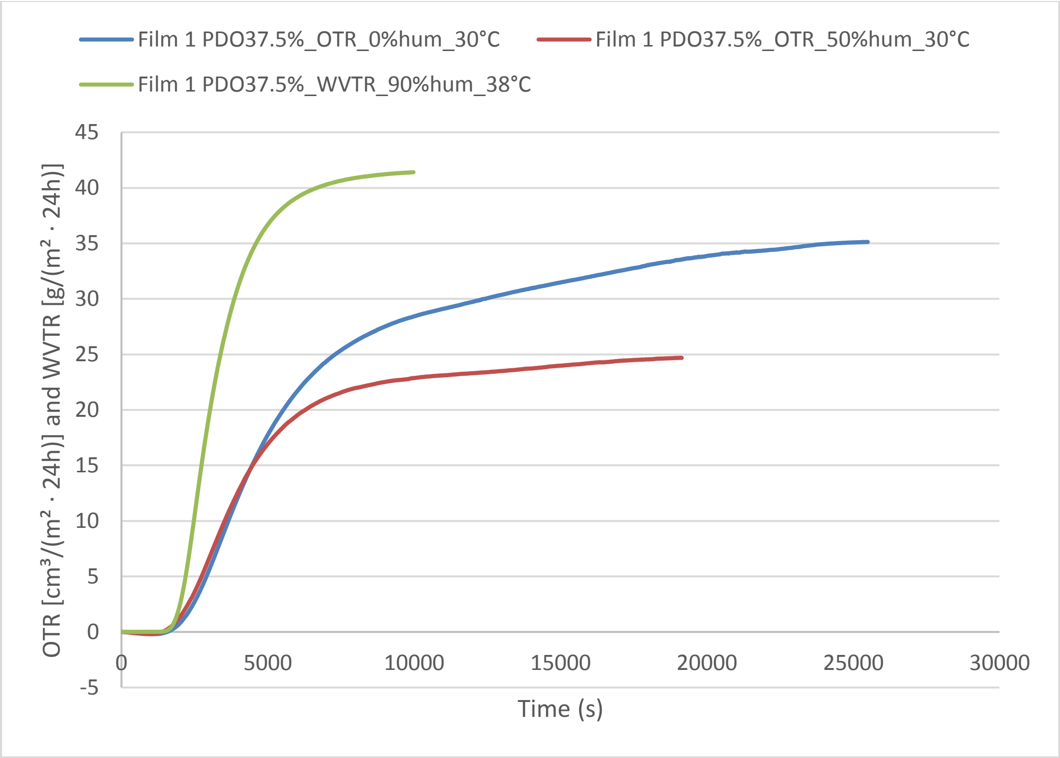

Figure S42. Permeability measurements PISOX PDO 37.5% (film 1)

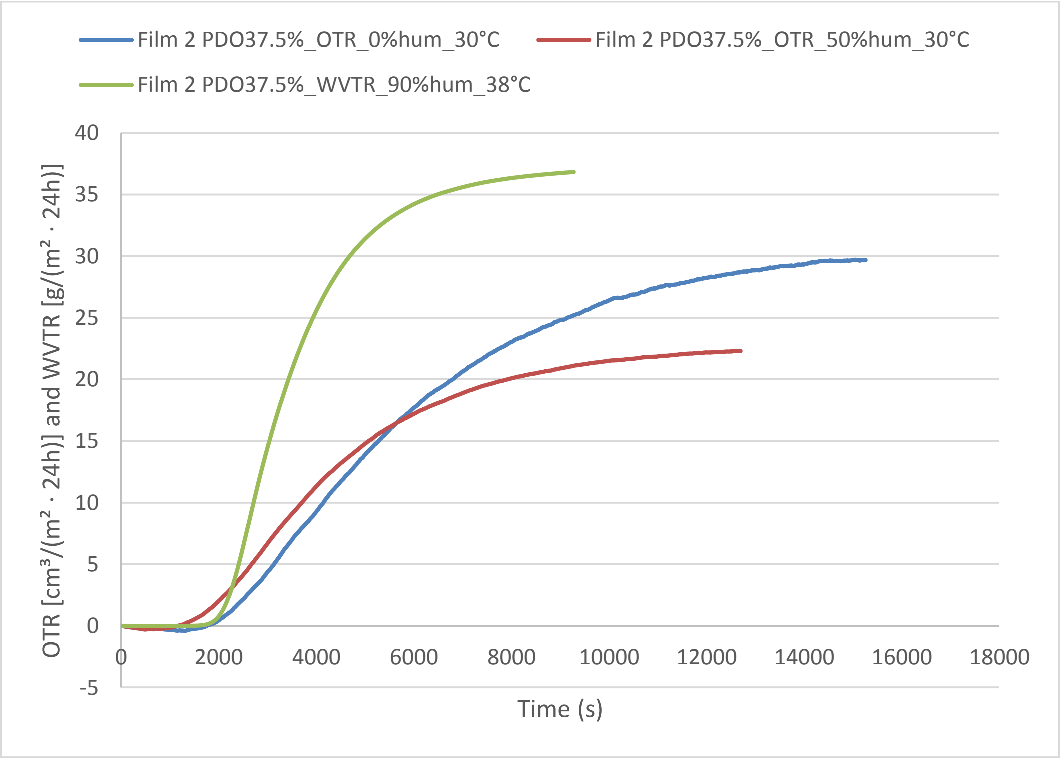

Figure S43. Permeability measurements PISOX PDO 37.5% (film 2)

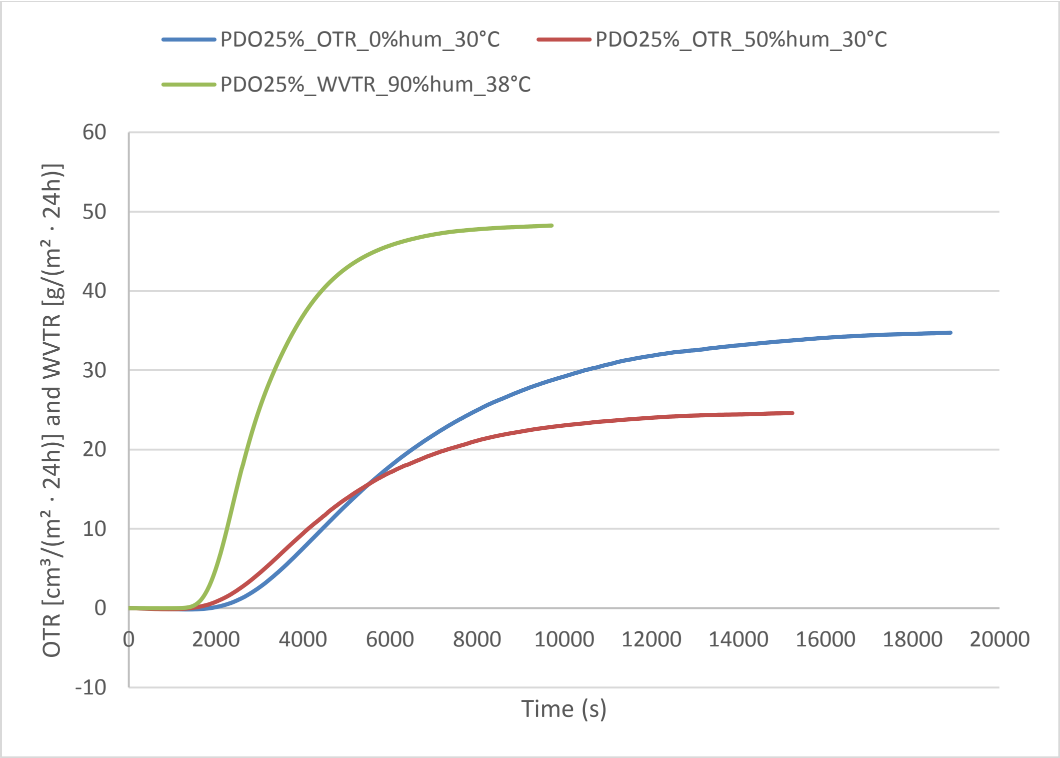

Figure S44. Permeability measurements PISOX PDO 25%

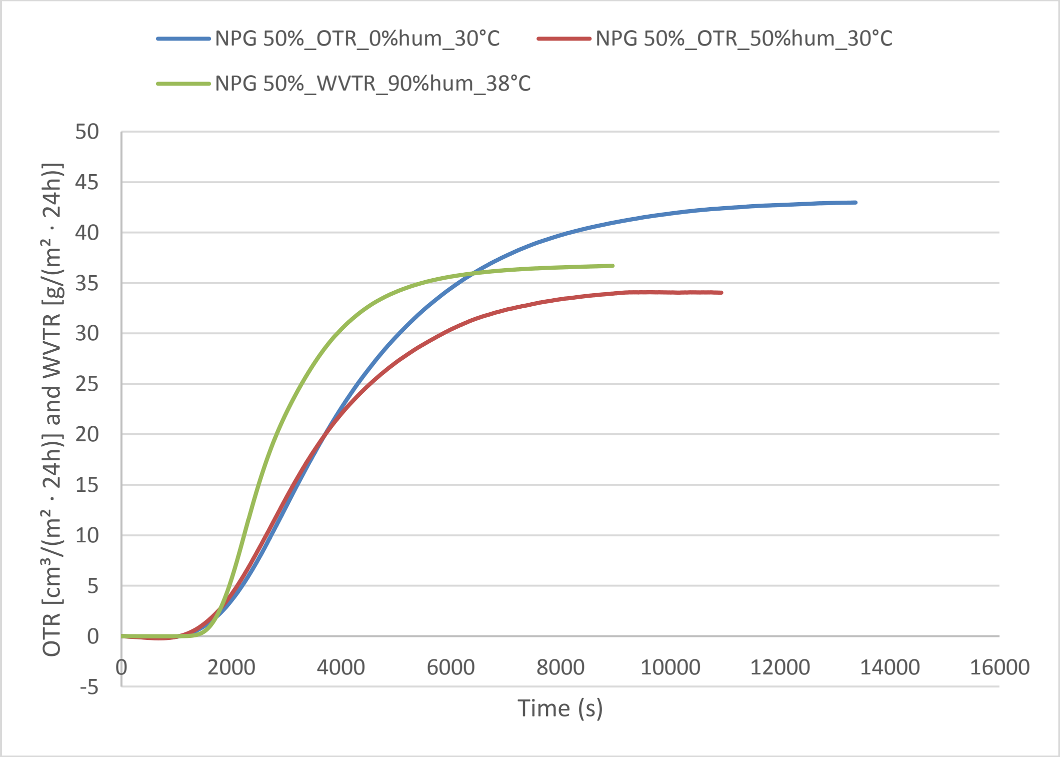

Figure S45. Permeability measurements PISOX NPG 50%

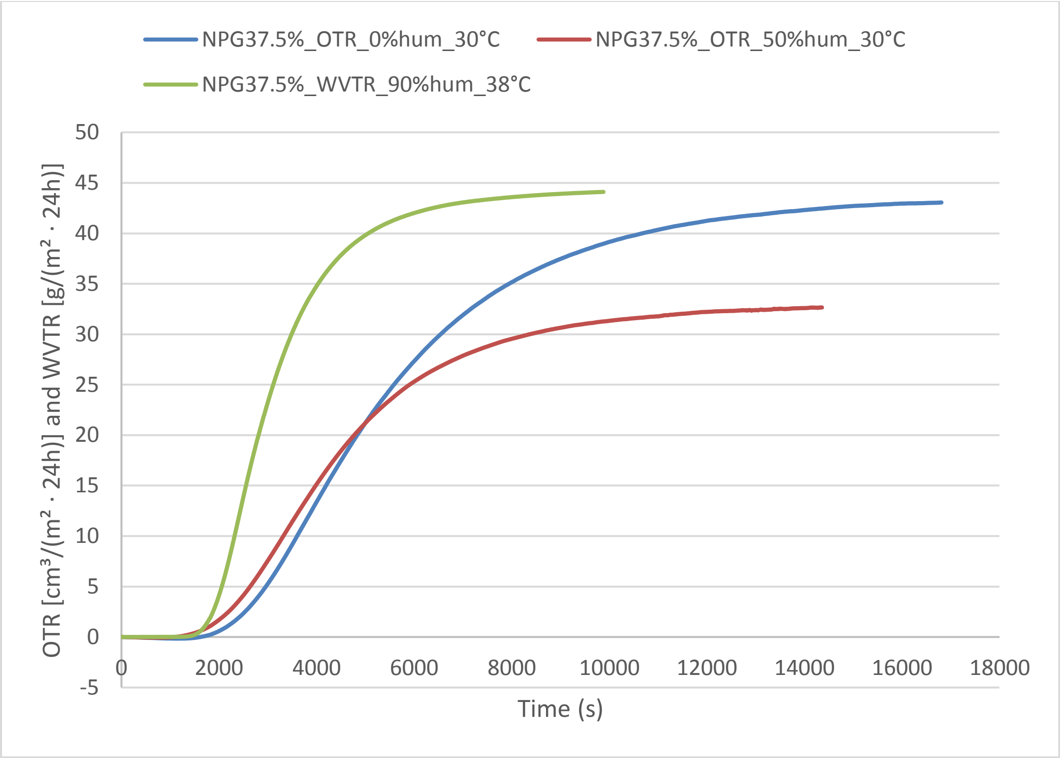

Figure S46. Permeability measurements PISOX NPG 37.5%

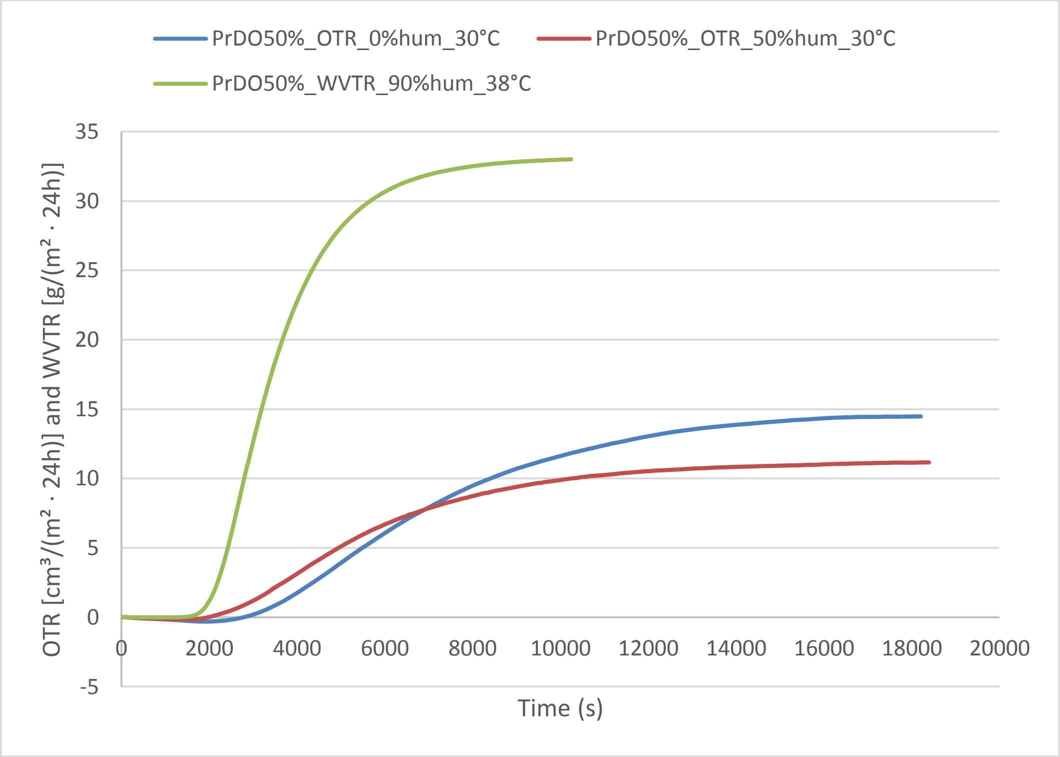

Figure S47. Permeability measurements PISOX PrDO 50%

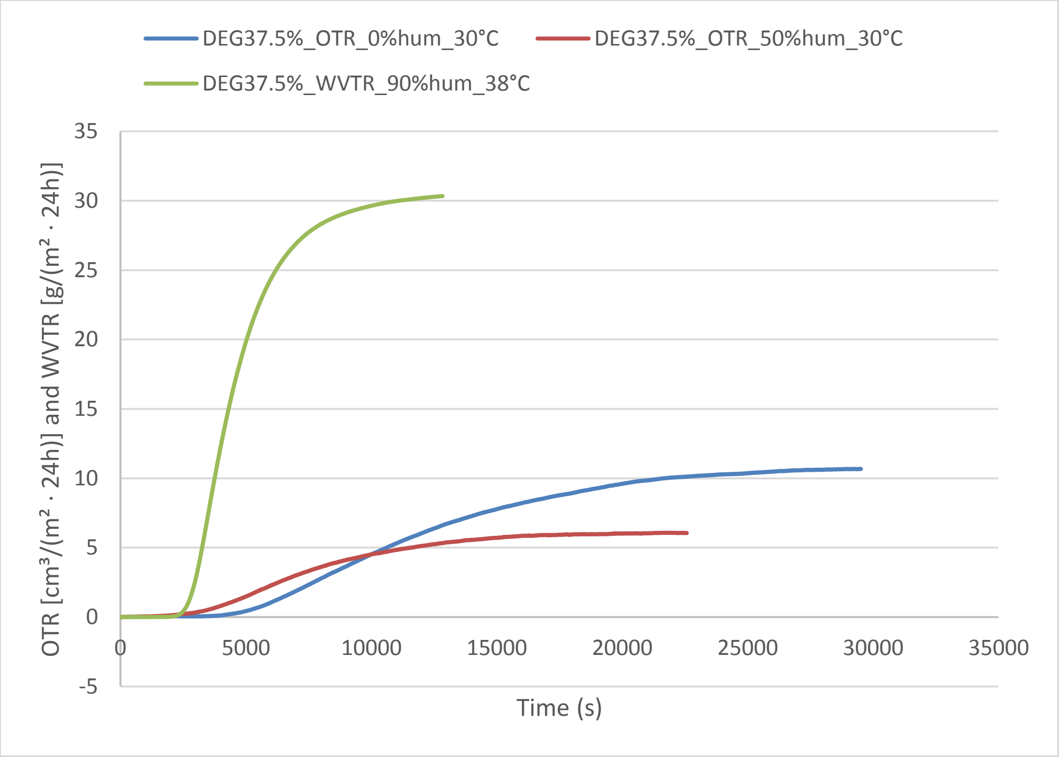

Figure S48. Permeability measurements PISOX DEG 37.5%

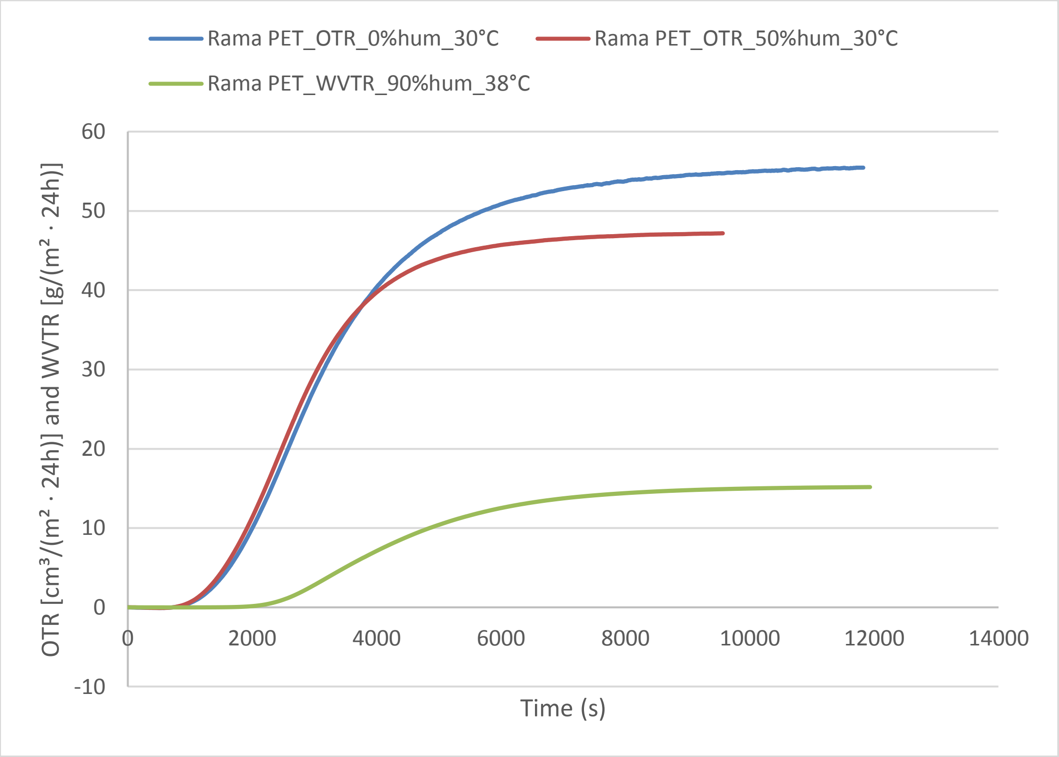

Figure S49. Permeability measurements Rama PET

Tensile testing data

Table S6. All Processed tensile data used for the overview of PISOX copolymer tensile strength.

Experiment	Composition	Young's modulus		Maximum Tensile stress		Tensile stress at Yield (Offset 0.002 mm/mm)		Elongation at Break	Samples
		(MPa)		(MPa)		(MPa)		Max found (%)	(amount)
		Value	Standarddev	Value	Standarddev	Value	Standarddev	Value	Value
Reference	Tritan	1720	50	47.1	0.4	32.5	0.5	182.4	3
Reference	ABS	2618	29	51.0	0.0	43.4	0.5	12.4	2
Reference	PET	2666	194	70.5	0.2	50.1	2.3	107.3	3
SSA013	HDO 25%	3118	44	75.0	4.7	52.6	1.5	186.8	3
KMA67B	PDO 37.5%	3054	56	76.1	2.7	54.3	3.3	219.2	4
KMA69	PDO 25%	3350	267	76.3	3.6	54.4	2.9	183.7	4
KMA75B	NPG 37.5%	3477	222	78.0	1.2	59.2	7.6	182.3	4
KMA71	CHDM 50%	2559	69	62.2	4.0	44.0	0.9	196.2	3
KMA74B	DEG 37.5%	3922	88	86.7	1.2	62.1	0.7	174.7	4
KMA101	PISOX 100%	3592	134	59.8	5.9	45.8	1.1	8.0	4

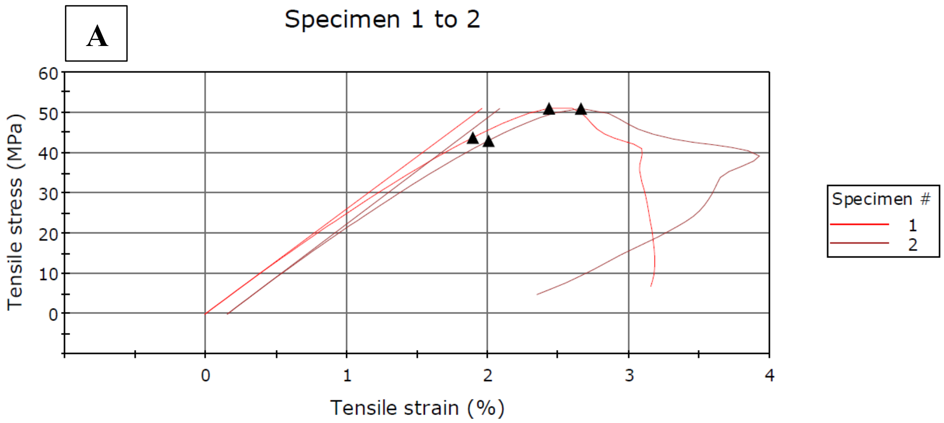

B	Modulus (Automatic Young's)	Load at Maximum Tensile stress	Maximum Tensile stress	Extension at Break (Standard)	snelheid	Tensile strain at Break (Standard)	Time at Break (Standard)	Tensile stress at Yield (Offset 0.002 mm/mm)	Displacement (Strain 1) at Break (Standard)	Axial Gauge Length (Strain Source)
	(MPa)	(N)	(MPa)	(mm)	(mm/min)	(mm/mm)	(sec)	(MPa)	(mm)	(mm)
1	2597	511	51.1	2.24	50	0.03	2.7	43.8	1.0	25
2	2638	510	51.0	2.48	50	0.03	3.0	43.0	1.1	25
Maximum	2638	511	51.1	2.48	50	0.03	3.0	43.8	1.1	25
Mean	2618	510	51.0	2.36	50	0.03	2.8	43.4	1.0	25
Minimum	2597	510	51.0	2.24	50	0.03	2.7	43.0	1.0	25
Standard Deviation	29	1	0.1	0.17	0	0.00	0.2	0.5	0.0	0

Figure S50. ABS reference tensile test: (A) Tensile readout (B) Tensile results

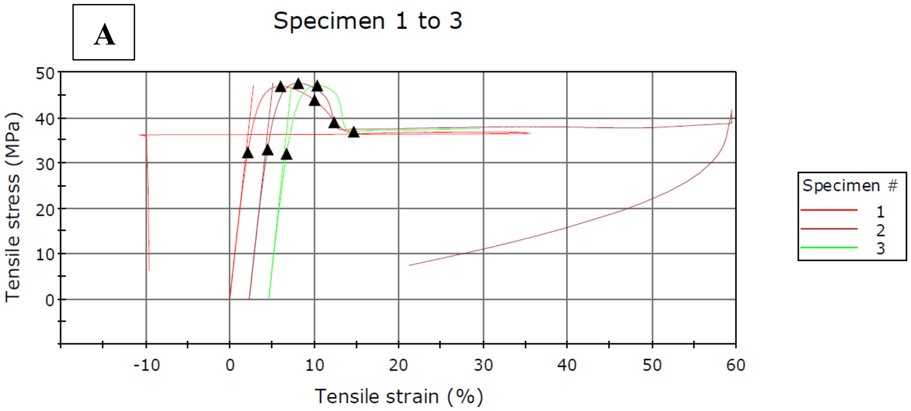

B	Modulus (Automatic Young's)	Load at Maximum Tensile stress	Maximum Tensile stress	Extension at Break (Standard)	snelheid	Tensile strain at Break (Standard)	Time at Break (Standard)	Tensile stress at Yield (Offset 0.002 mm/mm)	Displacement (Strain 1) at Break (Standard)	Axial Gauge Length (Strain Source)
	(MPa)	(N)	(MPa)	(mm)	(mm/min)	(mm/mm)	(sec)	(MPa)	(mm)	(mm)
1	1681	478	46.9	11.02	50	-0.10	13.2	32.4	-2.5	25
2	1704	475	47.5	36.47	50	0.57	43.8	33.0	14.5	25
3	1776	470	47.0	12	50	0.25	14.4	32.0	6.5	25
Maximum	1776	478	47.5	36.47	50	0.57	43.8	33.0	14.5	25
Mean	1720	474	47.1	19.83	50	0.24	23.8	32.5	6.2	25
Minimum	1681	470	46.9	11.02	50	-0.10	13.2	32.0	-2.5	25
Standard Deviation	50	4	0.4	14.42	0	0.33	17.3	0.5	8.5	0

Figure S51. Tritan reference tensile test: (A) Tensile readout (B) Tensile results

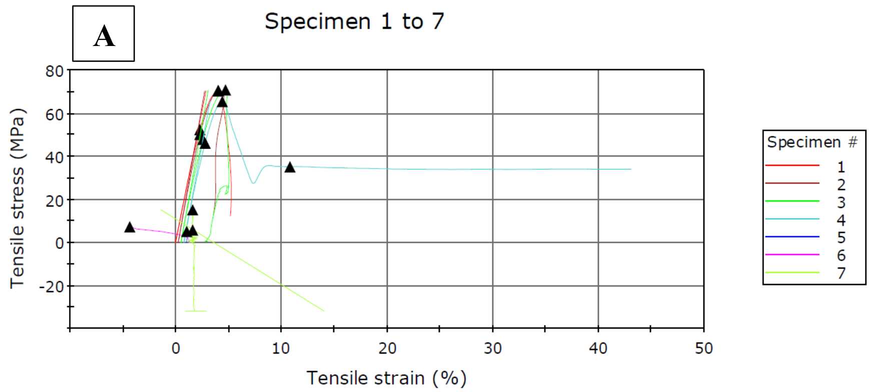

B	Modulus (Automatic Young's)	Load at Maximum Tensile stress	Maximum Tensile stress	Extension at Break (Standard)	snelheid	Tensile strain at Break (Standard)	Time at Break (Standard)	Tensile stress at Yield (Offset 0.002 mm/mm)	Displacement (Strain 1) at Break (Standard)	Axial Gauge Length (Strain Source)
	(MPa)	(N)	(MPa)	(mm)	(mm/min)	(mm/mm)	(sec)	(MPa)	(mm)	(mm)
1	2444	704	70.4	2.4	50	0.04	2.9	52.4	1.3	25
2	2803	703	70.3	2.51	50	0.04	3.0	50.2	0.6	25
3	2750	707	70.7	12.21	50	0.02	14.7	47.8	0.9	25
4	2644	653	65.3	12.01	5	0.42	144.1	46.1	10.6	25
5		53	5.3	12.2	50	0.00	0.2		-0.8	25
6		73	7.3	12.46	50	-0.06	0.3		-1.6	25
7	-305	153	15.3	21.46	50	0.00	10.8	5.8	-1.2	25
Maximum	2803	707	70.7	21.46	50	0.42	144.1	52.4	10.6	25
Mean	2067	435	43.5	10.75	43.57	0.07	25.1	40.5	1.4	25
Minimum	-305	53	5.3	2.4	5	-0.06	0.2	5.8	-1.6	25

Figure S52. PET Reference tensile tests: (A) Tensile readout (B) Tensile results

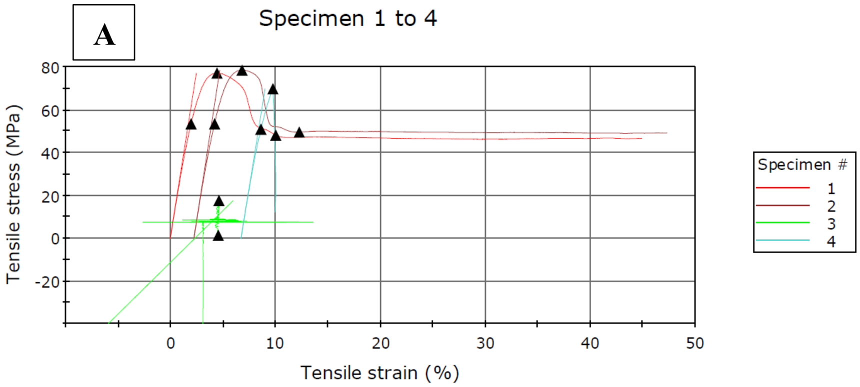

B	Modulus (Automatic Young's)	Load at Maximum Tensile stress	Maximum Tensile stress	Extension at Break (Standard)	snelheid	Tensile strain at Break (Standard)	Time at Break (Standard)	Tensile stress at Yield (Offset 0.002 mm/mm)	Displacement (Strain 1) at Break (Standard)	Axial Gauge Length (Strain Source)
	(MPa)	(N)	(MPa)	(mm)	(mm/min)	(mm/mm)	(sec)	(MPa)	(mm)	(mm)
1	3107	601	77.0	12.02	5	0.45	144.2	53.4	11.0	25
2	3166	611	78.4	12	5	0.45	144.1	53.4	11.3	25
3	483	138	17.6	37.36	5	-0.01	304.3	1.5	-0.9	25
4	3080	544	69.7	1.88	5	0.03	22.5	50.9	0.9	25
Maximum	3166	611	78.4	37.36	5	0.45	304.3	53.4	11.3	25
Mean	2459	473	60.7	15.82	5	0.23	153.8	39.8	5.6	25
Minimum	483	138	17.6	1.88	5	-0.01	22.5	1.5	-0.9	25
Standard Deviation	1318	226	29.0	15.14	0	0.26	115.6	25.6	6.5	0

Figure S53. PISOX HDO 25% tensile tests: (A) Tensile readout (B) Tensile results

A

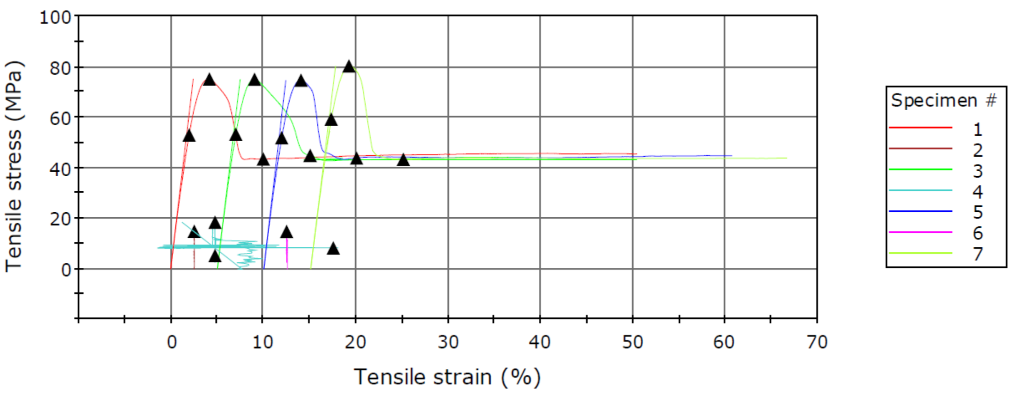

B	Modulus (Automatic Young's)	Load at Maximum Tensile stress	Maximum Tensile stress	Extension at Break (Standard)	snelheid	Tensile strain at Break (Standard)	Time at Break (Standard)	Tensile stress at Yield (Offset 0.002 mm/mm)	Displacement (Strain 1) at Break (Standard)	Axial Gauge Length (Strain Source)
	(MPa)	(N)	(MPa)	(mm)	(mm/min)	(mm/mm)	(sec)	(MPa)	(mm)	(mm)
1	3068	585	75.0	13.24	5	0.50	158.9	52.9	12.8	25
2		116	14.9	13.82	5	0.00	6.9		-1.1	25
3	3032	584	74.9	12.91	5	0.14	154.9	53.3	3.4	25
4	-285	144	18.4	43.64	5	-0.03	368.7	5.2	-1.0	25
5	3122	581	74.5	13.38	5	0.51	160.6	51.8	12.8	25
6		116	14.8	43.83	5	0.00	365.4		-1.0	25
7	2991	625	80.2	13.88	50	0.51	16.7	59.2	12.9	25
8		106	13.6	14.29	5	0.00	4.9	5.3	-1.0	25
9		121	15.5	19.83	50	0.00	6.6	-16.2	-1.0	25
Maximum	3122	625	80.2	43.83	50	0.51	368.7	59.2	12.9	25
Mean	2386	331	42.4	20.98	15	0.18	138.2	30.2	4.1	25
Minimum	-285	106	13.6	12.91	5	-0.03	4.9	-16.2	-1.1	25
Standard Deviation	1494	250	32.1	13.07	19.84	0.25	147.1	31.0	6.7	0

Figure S54. PISOX PDO-37.5% tensile tests: (A) Tensile readout (B) Tensile results

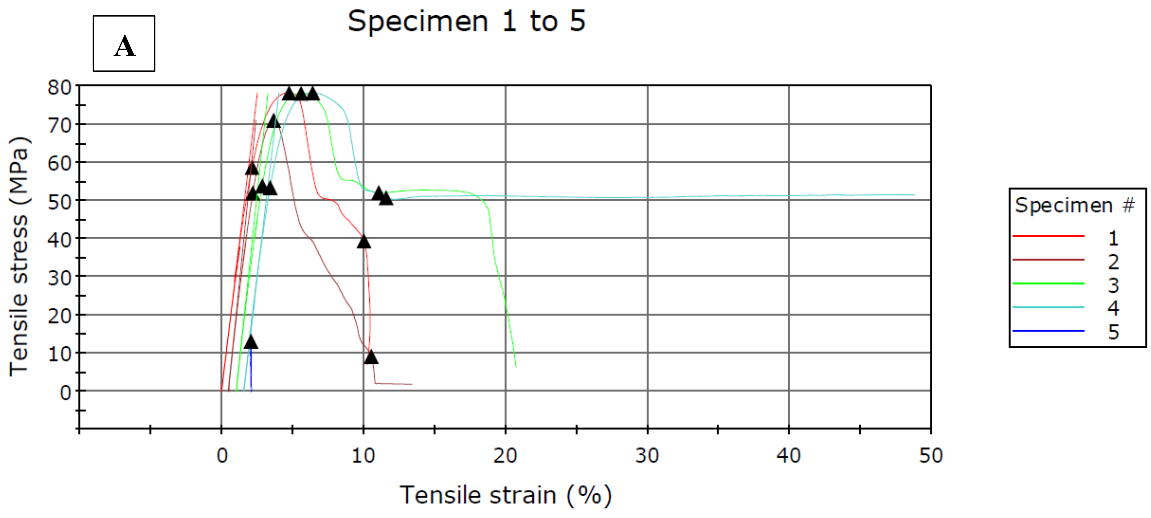

B	Modulus (Automatic Young's)	Load at Maximum Tensile stress	Maximum Tensile stress	Extension at Break (Standard)	snelheid	Tensile strain at Break (Standard)	Time at Break (Standard)	Tensile stress at Yield (Offset 0.002 mm/mm)	Displacement (Strain 1) at Break (Standard)	Axial Gauge Length (Strain Source)
	(MPa)	(N)	(MPa)	(mm)	(mm/min)	(mm/mm)	(sec)	(MPa)	(mm)	(mm)
1	3093	609	78.1	3.13	5	0.10	37.6	58.6	2.8	25
2	3646	553	70.9	3.59	5	0.13	43.1	51.9	0.8	25
3	3502	608	78.0	5.41	5	0.18	64.9	53.8	2.7	25
4	3158	609	78.1	13.2	5	0.47	158.4	53.4	11.5	25
5		103	13.2	36.74	5	0.00	282.4		-1.0	25
Maximum	3646	609	78.1	36.74	5	0.47	282.4	58.6	11.5	25
Mean	3350	497	63.7	12.41	5	0.18	117.3	54.4	3.4	25
Minimum	3093	103	13.2	3.13	5	0.00	37.6	51.9	-1.0	25
Standard Deviation	267	221	28.4	14.19	0	0.18	104.4	2.9	4.8	0

Figure S55. PISOX PDO 25% tensile tests: (A) Tensile readout (B) Tensile results

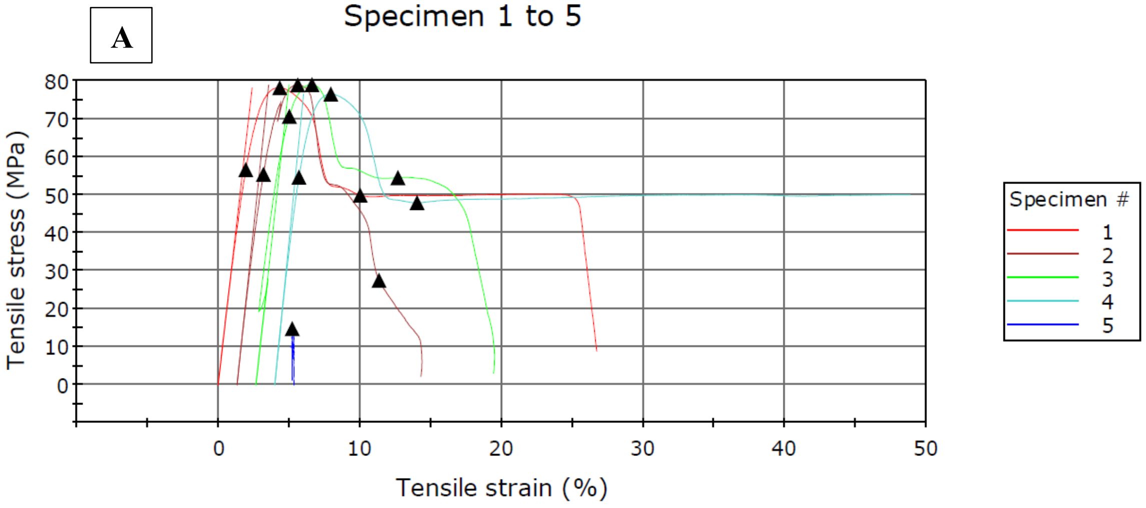

B	Modulus (Automatic Young's)	Load at Maximum Tensile stress	Maximum Tensile stress	Extension at Break (Standard)	snelheid	Tensile strain at Break (Standard)	Time at Break (Standard)	Tensile stress at Yield (Offset 0.002 mm/mm)	Displacement (Strain 1) at Break (Standard)	Axial Gauge Length (Strain Source)
	(MPa)	(N)	(MPa)	(mm)	(mm/min)	(mm/mm)	(sec)	(MPa)	(mm)	(mm)
1	3256	609	78.0	7.01	5	0.25	84.1	56.5	6.6	25
2	3505	614	78.8	3.72	5	0.13	44.6	55.3	3.5	25
3	3374	615	78.9	4.76	5	0.17	57.1	70.5	4.2	25
4	3772	596	76.4	12.68	5	0.45	152.2	54.6	9.2	25
5		115	14.8	36.46	5	0.00	285.4		-1.1	25
Maximum	3772	615	78.9	36.46	5	0.45	285.4	70.5	9.2	25
Mean	3477	510	65.4	12.93	5	0.20	124.7	59.2	4.5	25
Minimum	3256	115	14.8	3.72	5	0.00	44.6	54.6	-1.1	25
Standard Deviation	222	221	28.3	13.61	0	0.17	99.0	7.6	3.8	0

Figure S56. PISOX NPG 37.5% tensile tests: (A) Tensile readout (B) Tensile results

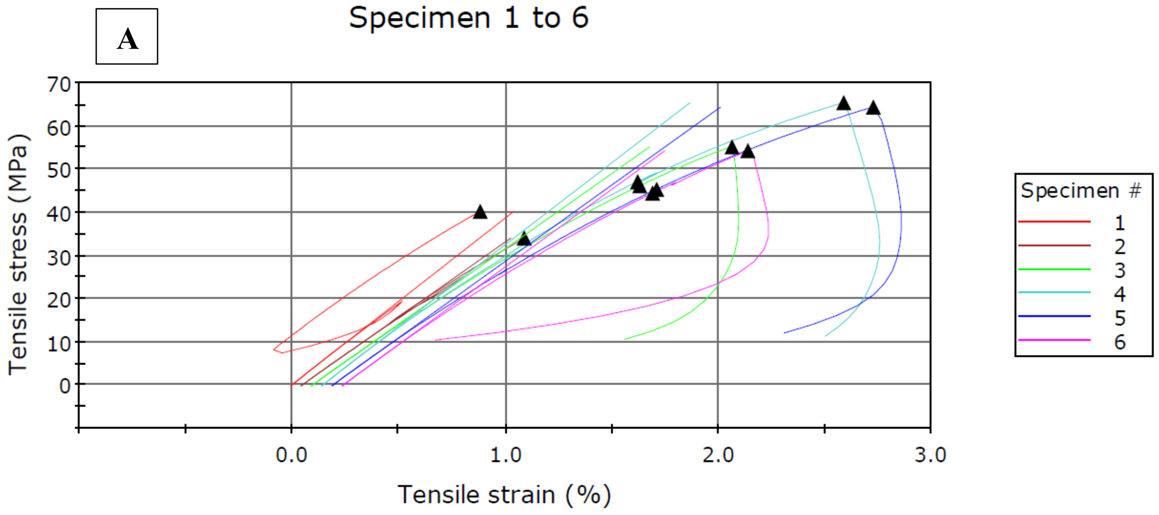

B	Modulus (Automatic Young's)	Load at Maximum Tensile stress	Maximum Tensile stress	Extension at Break (Standard)	snelheid	Tensile strain at Break (Standard)	Time at Break (Standard)	Tensile stress at Yield (Offset 0.002 mm/mm)	Displacement (Strain 1) at Break (Standard)	Axial Gauge Length (Strain Source)
	(MPa)	(N)	(MPa)	(mm)	(mm/min)	(mm/mm)	(sec)	(MPa)	(mm)	(mm)
1	3850	314	40.3	0.95	5	0.01	11.4		-2.0	25
2	3465	266	34.2	0.71	5	0.01	8.6		0.3	25
3	3475	430	55.2	1.04	5	0.02	12.5	46.2	0.3	25
4	3782	510	65.4	1.6	5	0.02	19.1	47.1	-0.7	25
5	3526	502	64.4	1.28	5	0.03	15.3	45.4	1.7	25
6	3583	423	54.3	1.04	5	0.02	12.5	44.5	0.7	25
Maximum	3850	510	65.4	1.6	5	0.03	19.1	47.1	1.7	25
Mean	3614	408	52.3	1.1	5	0.02	13.2	45.8	0.1	25
Minimum	3465	266	34.2	0.71	5	0.01	8.6	44.5	-2.0	25
Standard Deviation	164	99	12.7	0.3	0	0.01	3.6	1.1	1.2	0

Figure S57. PISOX 100% tensile tests: (A) Tensile readout (B) Tensile results

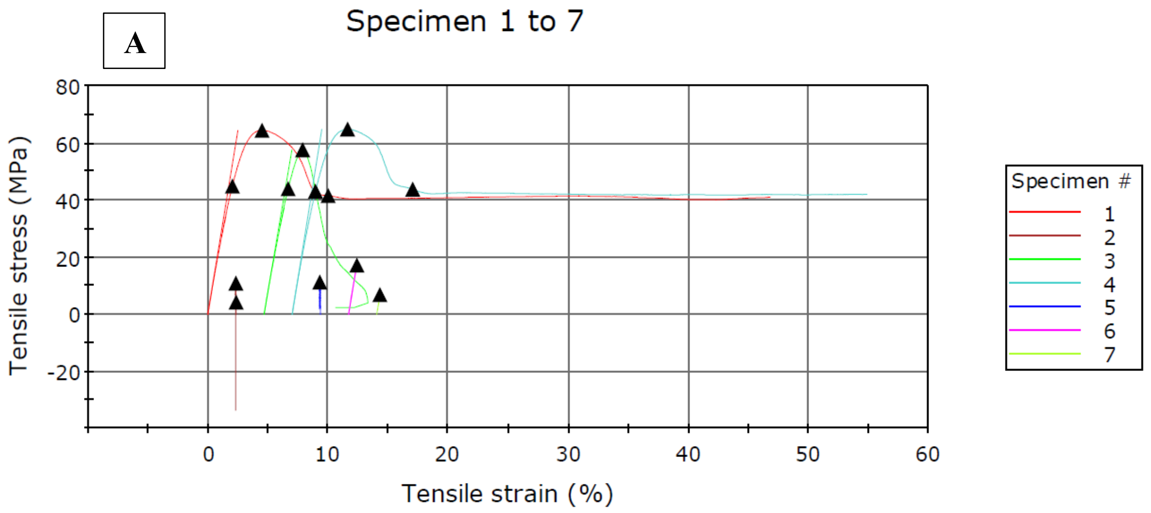

B	Modulus (Automatic Young's)	Load at Maximum Tensile stress	Maximum Tensile stress	Extension at Break (Standard)	snelheid	Tensile strain at Break (Standard)	Time at Break (Standard)	Tensile stress at Yield (Offset 0.002 mm/mm)	Displacement (Strain 1) at Break (Standard)	Axial Gauge Length (Strain Source)
	(MPa)	(N)	(MPa)	(mm)	(mm/min)	(mm/mm)	(sec)	(MPa)	(mm)	(mm)
1	2560	502	64.3	12.27	5	0.47	147.2	44.9	11.9	25
2		85	10.9	28.7	5	0.00	197.2	4.3	-1.1	25
3	2489	449	57.6	2.79	5	0.07	33.4	44.0	2.2	25
4	2627	504	64.7	12.44	5	0.48	149.3	43.1	11.9	25
5		88	11.3	39.24	5	0.00	321.5		-1.1	25
6	2607	135	17.3	2.92	5	0.00	35.0		-0.7	25
7		54	6.9	1.08	5	0.00	12.9		-0.8	25
8	3403	504	64.6	5.84	5	0.04	70.1	43.0	-1.3	25
Maximum	3403	504	64.7	39.24	5	0.48	321.5	44.9	11.9	25
Mean	2737	290	37.2	13.16	5	0.13	120.8	35.8	2.6	25
Minimum	2489	54	6.9	1.08	5	0.00	12.9	4.3	-1.3	25
Standard Deviation	376	215	27.6	13.81	0	0.21	104.8	17.6	5.8	0

Figure S58. PISOX CHDM50% tensile tests: (A) Tensile readout (B) Tensile results

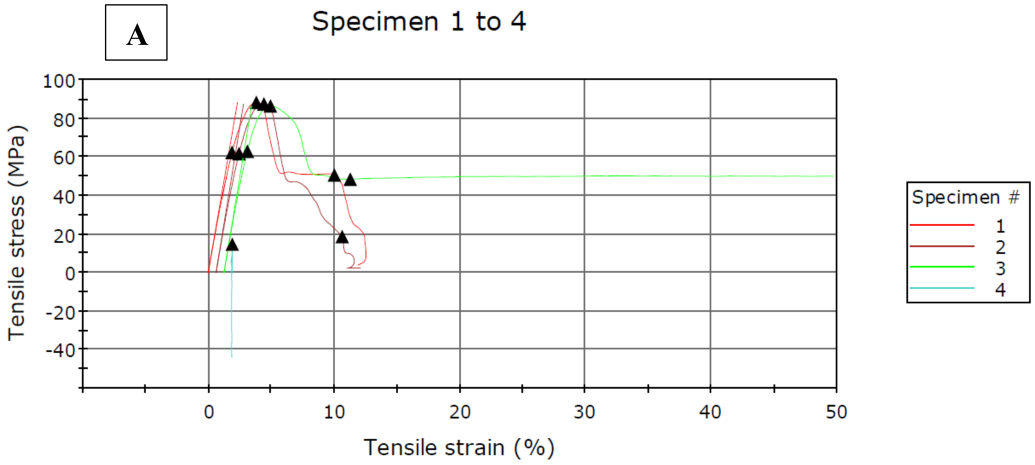

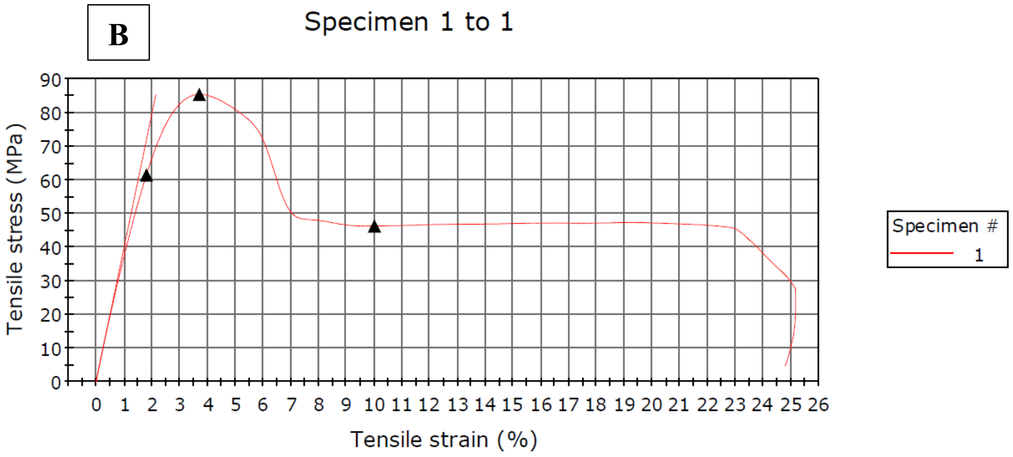

C	Modulus (Automatic Young's)	Load at Maximum Tensile stress	Maximum Tensile stress	Extension at Break (Standard)	snelheid	Tensile strain at Break (Standard)	Time at Break (Standard)	Tensile stress at Yield (Offset 0.002 mm/mm)	Displacement (Strain 1) at Break (Standard)	Axial Gauge Length (Strain Source)
	(MPa)	(N)	(MPa)	(mm)	(mm/min)	(mm/mm)	(sec)	(MPa)	(mm)	(mm)
1	3799	687	88.1	3.59	5	0.12	43.1	62.3	3.4	25
2	4005	681	87.3	3.15	5	0.11	37.8	61.6	2.8	25
3	3936	672	86.2	12.57	5	0.48	150.8	62.9	11.3	25
4		116	14.9	34.93	5	0.00	268.4		-1.1	25
(1)	3947	666	85.3	6.59	5	0.25	79.1	61.4	6.1	25
Maximum	4005	687	88.1	34.93	5	0.48	268.4	62.9	11.3	25
Mean	3913	539	69.1	13.56	5	0.18	125.0	62.3	4.1	25
Minimum	3799	116	14.9	3.15	5	0.00	37.8	61.6	-1.1	25
Standard Deviation	105	282	36.2	14.9	0	0.21	108.9	0.6	5.2	0

Figure S59. PISOX DEG 37.5% tensile tests: (A and B) Tensile readout (C) Tensile results

Young's modulus	2450	MPa
Yield strength	50.7	MPa
Tensile strength	50.7	MPa
Strength at break	35	MPa
Yield strain	2.61	% strain
Strain at tensile strength	2.61	% strain
Strain at break	12	% strain
Work up to tensile strength	0.195	J
Work up to break	1.15	J
Tensile bar geometry	ISO 1BA tensile bar	
Tensile bar thickness (h)	2.02	mm
Tensile bar width (b)	5.05	mm

Figure S60. External measurements of reference tensile bar samples (ABS)

Young's modulus	1680	MPa
Yield strength	46.1	MPa
Tensile strength	46.1	MPa
Strength at break	41.2	MPa
Yield strain	6.05	% strain
Strain at tensile strength	6.05	% strain
Strain at break	71.6	% strain
Work up to tensile strength	0.518	J
Work up to break	7.04	J
Tensile bar geometry	ISO 1BA tensile bar	
Tensile bar thickness (h)	2.02	mm
Tensile bar width (b)	5.07	mm

Figure S61. External measurements of reference tensile bar samples (Tritan)

Injection molding settings and details (Thermo Scientific HAAKE Minijet II)

Table S7 Injection molding processing parameters

Sample	*Cylinder temperature (°C)*	*Mold temperature (°C)*	*Pressure (bar)*	*Time*
HDO 25%	245	50	1000	15sec
CHDM 50%	235	50	1000	15sec
PrDO 25%	210	50	1000	15sec
NPG 37.5%	225	50	1000	15sec
PrDO 37.5%	190	50	1000	15sec
DEG 37.5%	190	50	1000	15sec
PISOX 100%	260	50	1000	15sec

Table S8 Molecular weight before and after injection molding of the samples.

Before				After			% of original		
Compound	Mn	Mw	Mw/Mn	Mn	Mw	Mw/Mn	Mn	Mw	Mw/Mn
25%HDO	4.4E+04	8.2E+04	1.85	3.1E+04	5.3E+04	1.73	70	65	93
50% CHDM	4.9E+04	9.0E+04	1.84	2.8E+04	4.9E+04	1.78	57	55	97
25%PDO	2.8E+04	4.9E+04	1.75	2.1E+04	4.4E+04	2.14	73	89	122
37.5% NPG	6.8E+04	1.3E+05	1.95	3.9E+04	8.3E+04	2.10	57	62	108
37.5% PDO	3.9E+04	7.0E+04	1.81	3.0E+04	5.6E+04	1.84	78	80	102
37.5% DEG	3.0E+04	4.8E+04	1.61	2.5E+04	4.5E+04	1.82	83	94	113

GPC data overview

Table S9 Determined Mn, Mw and Đ of the PISOX copolymers.

		Detector A			Detector B		
		Mn	Mw	Mw/Mn	Mn	Mw	Mw/Mn
HDO 37.5%	KMA67A	5.24E+04	9.15E+04	1.75	5.44E+04	1.05E+05	1.93
PDO 37.5%	KMA 67B	3.17E+04	6.03E+04	1.90	3.86E+04	6.97E+04	1.81
PDO 25%	KMA 69	1.99E+04	4.23E+04	2.13	2.83E+04	4.94E+04	1.75
CHDM 50%	KMA 71	4.24E+04	8.04E+04	1.90	4.88E+04	8.97E+04	1.84
DEG 37.5%	KMA 74B	2.39E+04	4.15E+04	1.74	2.99E+04	4.82E+04	1.61
NPG 37.5%	KMA 75B	5.87E+04	1.18E+05	2.00	6.85E+04	1.33E+05	1.95
PrDO 50%	SSA 003	2.88E+04	4.90E+04	1.70	3.71E+04	5.65E+04	1.52
NPG 50%	SSA004	3.06E+04	5.39E+04	1.76	3.56E+04	6.33E+04	1.78
HDO 25%	SSA013	3.80E+04	7.46E+04	1.96	4.40E+04	8.17E+04	1.85

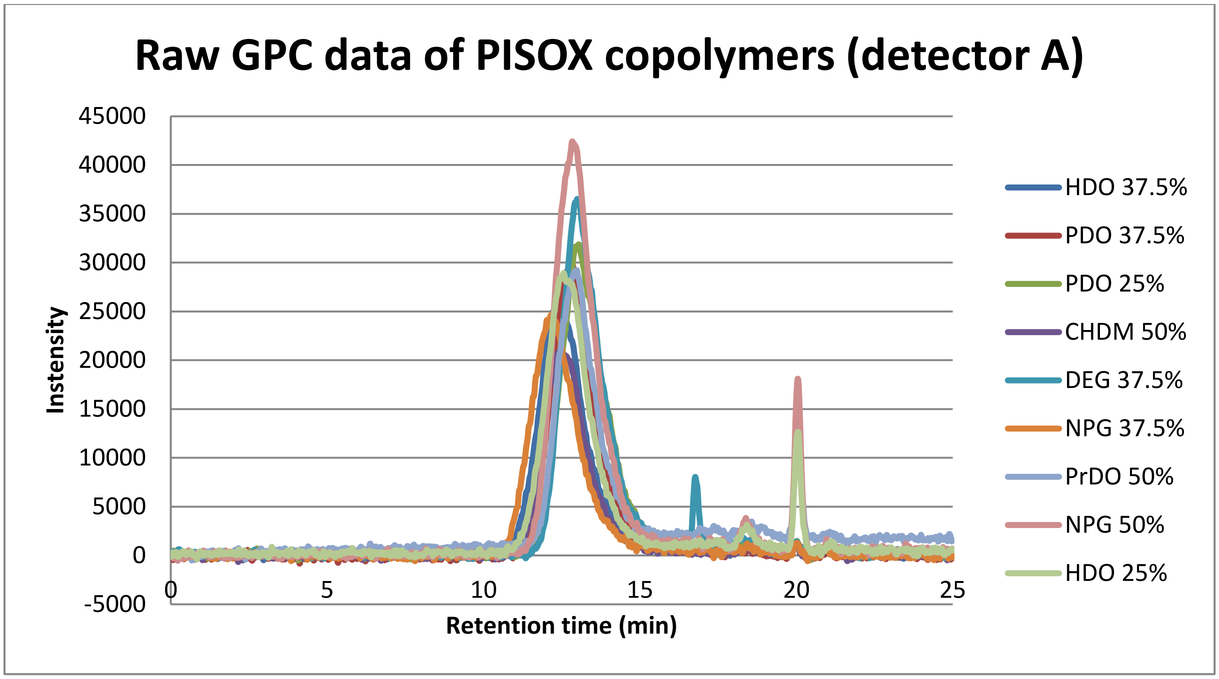

Figure S62. The GPC elution curves of the PISOX copolymers (UV detector)

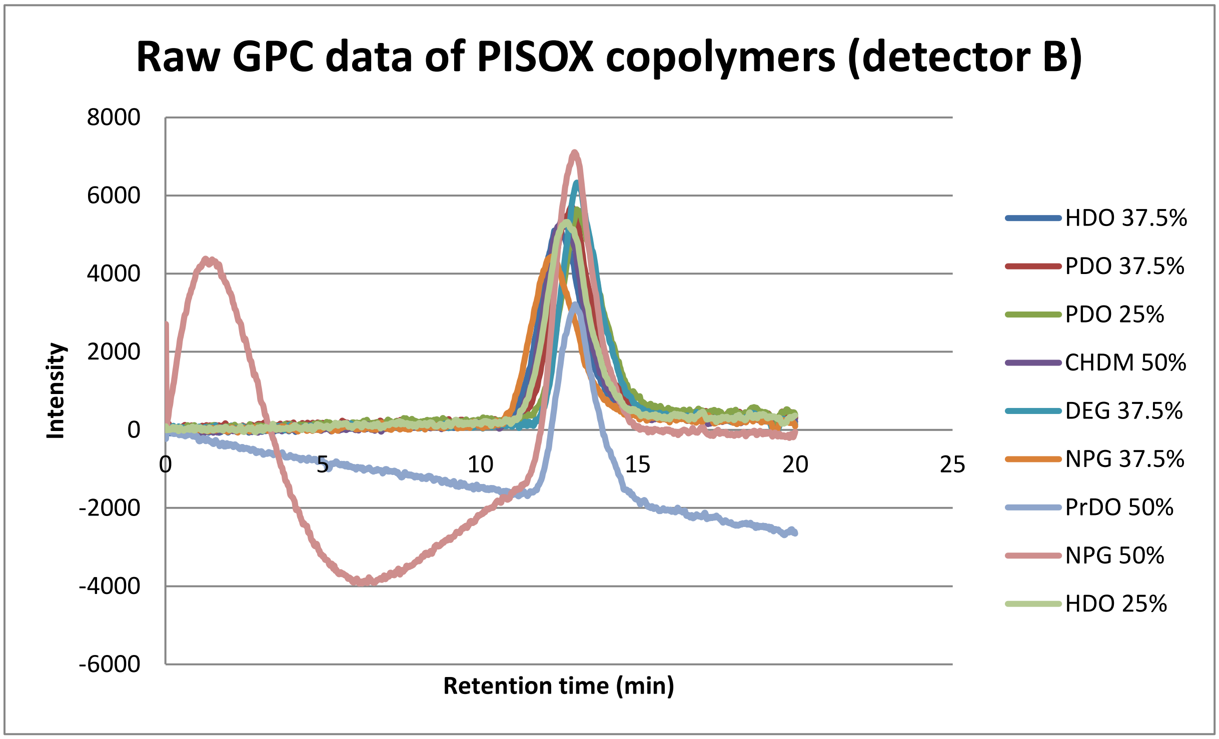

Figure S63. The GPC elution curves of the PISOX copolymers (Refractive detector)

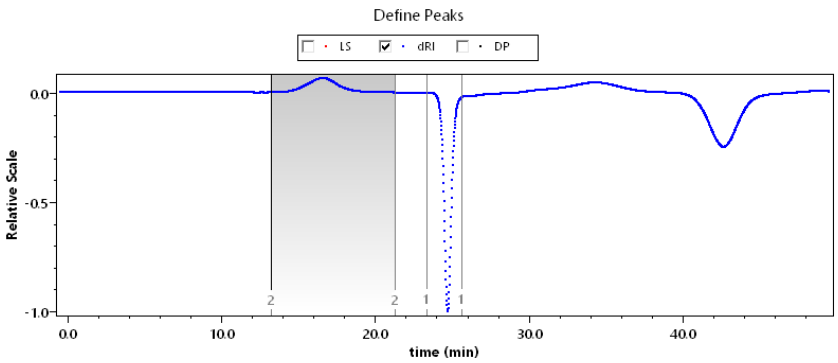

Results

Peak Results

	flow marker	dRI
**Molar mass moments (g/mol)**		
**Mn**	n/a	39639 (±14%)
**Mp**	0	59090 (±14%)
**Mw**	n/a	78296 (±14%)
**Polydispersity**		
**Mw/Mn**	n/a	1.98 (±20.40%)

Figure S64. GPC results of PISOX 100% (HFIP)

References

1. Nishihira, K., Tanaka, S., Nishida, Y. & Fujitsu, S. Process for preparing diaryl esters of oxalic acid. **WO 97/21660** (1997).

2. Yan, R. & Qiu, Z. Isothermal melt crystallization kinetics, melting behavior, and spherulitic morphology of novel biobased poly(hexylene oxalate). *RSC advances* **5**, 1425-14257 (2015), DOI:10.1039/c5ra22486a.

3. Garcia, J. J. & Miller, S. A. Polyoxalates from biorenewable diols via Oxalate Metathesis Polymerization. *Polymer chemistry* **5**, 955-961 (2014), DOI:10.1039/C3PY01185B.

4. Wang, L., Tu, Z., Liang, J. & Wei, Z. Poly (butylene oxalate-co-terephthalate): A PBAT-like but rapid hydrolytic degradation plastic. *J. Hazard. Mater.*, 134349 (2024).
